# Selective Stimulation of Duplicated Atlantic Salmon MHC Pathway Genes by Interferon-Gamma

**DOI:** 10.3389/fimmu.2020.571650

**Published:** 2020-10-06

**Authors:** Unni Grimholt, Johanna H. Fosse, Arvind Y. M. Sundaram

**Affiliations:** ^1^Norwegian Veterinary Institute, Oslo, Norway; ^2^Department of Medical Genetics, Oslo University Hospital, Oslo, Norway

**Keywords:** MHC pathways, whole genome duplications, interferon gamma, teleost, antigen presentation, homeologs, Atlantic salmon

## Abstract

Induction of cellular immune responses rely on Major histocompatibility complex (MHC) molecules presenting pathogenic peptides to T cells. Peptide processing, transport, loading and editing is a constitutive process in most cell types, but is accelerated upon infection. Recently, an unexpected complexity in the number of functional genes involved in MHC class I peptide cleavage, peptide transport, peptide loading and editing was found in teleosts, originating from the second and third whole genome duplication events. Salmonids have expanded upon this with functional duplicates also from a fourth unique salmonid whole genome duplication. However, little is known about how individual gene duplicates respond in the context of stimulation. Here we set out to investigate how interferon gamma (IFNg) regulates the transcription of immune genes in Atlantic salmon with particular focus on gene duplicates and MHC pathways. We identified a range of response patterns in Atlantic salmon gene duplicates, with upregulation of all duplicates for some genes, like interferon regulatory factor 1 (IRF1) and interferon induced protein 44-like (IFI44.L), but only induction of one or a few duplicates of other genes, such as TAPBP and ERAP2. A master regulator turned out to be the IRF1 and not the enhanceosome as seen in mammals. If IRF1 also collaborates with CIITA and possibly NLRC5 in regulating IFNg induction of MHCI and MHCII expression in Atlantic salmon, as in zebrafish, remains to be established. Altogether, our results show the importance of deciphering between gene duplicates, as they often respond very differently to stimulation and may have different biological functions.

## Introduction

Interferon gamma (IFNg) is a key activator of viral immunity and induces signaling through the JAK/ STAT pathway (1). IFNg binds and activates the interferon gamma receptor, thus initiating phosphorylation of Janus kinase 1 (JAK1) and JAK2 that again phosphorylate and activate the signal transducer and activator of transcription proteins (STAT) 1 and possibly STAT2. This activated STAT homo- or hetero-dimer then translocates to the nucleus where it binds to promoter gamma interferon activation sites (GAS) in IFNg inducible genes. One of the first elements induced by this STAT complex is IRF1, which collaborates with STAT in further transcriptional regulation of genes.

Activation of the JAK/STAT pathway induces transcription of a range of genes that are important for the cellular defense against virus infection, such as the MHC genes and other genes involved in antigen processing and presentation. Classical MHC class I (MHCI) genes are responsible for surveilling the intracellular space and present peptides from self- and non-self to CD8 positive T-cells thereby facilitating detection of virus-infected cells by the immune system (2). The MHCI molecule consists of an alpha chain non-covalently linked to beta2-microglobulin (b2m), where the alpha 1 and alpha 2 domain of the alpha chain are responsible for binding of self and non-self peptides. These two domains are also the ones with highest allelic diversity. The term classical refers to peptide binding MHC genes with high allelic diversity and a specific expression pattern such as the human HLA-A, HLA-B, and HLA-C genes with 3794, 4648, and 3503 registered protein alleles, respectively (3).

Expression of classical MHC genes is controlled by both transcriptional and posttranslational mechanisms. The canonical MHC promoter consists of a SXY sequence module that is bound by enhanceosome transcription factors consisting of RFX5, RFXAP, RFXANK, CREB/ATF1, NF-Y's and the MHCI transactivator NLRC5 or the MHCII transactivator CIITA (4, 5). This SXY motif is also present in b2m, TAP and LMP2 promoters, suggesting a similar transcriptional enhanceosome control (6). Additional MHCI promoter elements consist of interferon stimulated response elements (ISRE) and GAS recognized by interferon response factors (primarily IRF1) and STAT1/ STAT2 binding [reviewed in (7)]. Some MHCI promoters also have other motifs that can be activated by NF-kB, upstream stimulatory factors (USF) and SP1 binding (8).

Peptide loading of MHCI molecules is a multi-step process starting with the degradation of peptides in cytosol. Upon IFNg stimulation, an immunoproteasome is induced through replacement of the constitutive proteasome components PSMB5, −6, and −7 with interferon stimulated immune-proteasome components PSMB8, −9 and −10 (9). Peptides produced by the immunoproteasome have increased affinity for both the TAP1/TAP2 channel transporting the peptide into the endoplasmic reticulum (ER), as well as for MHC class I molecules. Two additional interferon-induced subunits called PSME1 and PSME2 provide an added regulatory element to the core immunoproteasome with debated effect on the MHCI peptide repertoire (10).

Peptides produced by the immunoproteasome are transported into the ER by an MHC-peptide specific transporter consisting of the TAP1 and TAP2 heterodimer [reviewed in Neefjes et al. (11)]. An empty MHCI /b2m molecule, stabilized by CALR and ERP57, is linked to the TAP transporter by a tapasin (TAPBP) molecule and this complex is called the peptide-loading complex (11). As the optimal length of MHCI peptides varies between 8 and 12 residues, some peptides need further trimming by ERAP1 and ERAP2 molecules to fit perfectly into the MHCI groove (12). The peptides may also be exchanged for better fit peptides by the tapasin-related molecule (TAPBPR) (13, 14) before the peptide-loaded MHCI molecule is transported to the cell surface for recognition by T-cells.

Whole genome duplication (WGD) is a widespread phenomenon in animals and plants. All vertebrates have experienced two rounds of whole genome duplications called the 2R-WGD. In teleosts, there has been a third WGD (3R-WGD), occurring ~350 million years ago separating the bony fishes from other ray-finned fishes (15). Functional remnants of both the 2R-WGD and the 3R-WGD are still present in teleosts today (16). Salmonids and carp experienced a more recent fourth WGD (4R-WGD), further adding to the gene complexity in these species (17, 18). At least in salmonids, many of the gene duplicates from 2-4R-WGD have been retained as functional copies (17).

We recently identified an unprecedented gene complexity in the antigen processing and presentation machinery in Atlantic salmon (16). For example, calreticulin (CALR), ERP57 and TAPBP all exist in functional gene duplicates originating from the second, third and fourth WGD. The TAPBP clade includes both previously identified TAPBP and TAPBP-related (TAPBPR) genes in addition to a newly identified TAPBP_like (TAPBPL) gene with low sequence identity to both TAPBP as well as TAPBPR gene sequences. Some genes are also present in single copies such as the classical Atlantic salmon MHC class I gene UBA, while its molecular partner, b2m, has expanded to 12 genes (16). Together, this provides an unprecedented peptide loading complexity of unknown functional relevance. Based on the observation that neo-functionalization of gene duplicates in Atlantic salmon was more frequent than sub-functionalization (17), this raises many questions regarding the function of these gene duplicates.

While Atlantic salmon UBA only exists as a single gene, there are currently 48 registered alleles for this locus in the IPD-MHC database (19). Atlantic salmon and rainbow trout UBA genes have promoter regions with regulatory elements consistent with IFNg induction (20, 21). The UBA gene belongs to the MHC class I U lineage, alongside non-classical genes, all assumed to bind peptides originating from the peptide loading machinery (22). One additional MHCI lineage, the Z lineage, is also assumed to bind peptides, while the remaining four MHCI lineage (S-, L-, H-, and P-) bind other, as yet undefined, ligands.

Although we know that teleosts have expressed remnants of the second, third and fourth WGDs, no studies have so far investigated how individual duplicates of given pathways respond to stimulation. Here we use recombinant IFNg stimulation to understand how individual Atlantic salmon gene duplicates respond, with particular focus on genes involved in MHC pathways.

## Materials and Methods

### Recombinant Interferon Gamma Production

DNA, consisting of the mature open reading frame of the Atlantic salmon IFNg sequence NM_001171804.1, inserted into the pET151/D-TOPO vector was purchased from GeneArt (Thermo Fisher Scientific, Oslo, Norway). The DNA clone was transfected into *E.coli* BL21 DE3 (Thermo Fisher Scientific, #C600003). Recombinant protein was isolated and purified following an established protocol (23). In short, 0.2 mM IPTG was used in the induction and the culture was grown at 28°C. The bacteria were harvested and sonicated on ice in lysis buffer [50 mM NaH_2_PO_4_, 300 mM NaCl, 10% glycerol (pH 8.0)], and the recombinant protein was purified on a Ni-NTA resin column (QIAGEN, Oslo, Norway). The purity of the recombinant IFNg (rIFNg) was checked on a 4–12% precast SDS-PAGE gel (Thermo Fisher Scientific) stained with SimplyBlue (Thermo Fisher Scientific). Protein concentration was measured with a Qubit Protein Assay kit (#Q33211, Thermo Fisher Scientific).

### Recombinant Interferon Gamma Stimulation

Salmon head kidney cells (SHK-1) (24) in passage 48 were seeded into 25 cm^2^ flasks using trypsin/EDTA totalling 4 × 10^5^ cells per each of the 18 flasks, and grown at 20°C overnight in Leibovitz L15 medium with L-glutamine (Merck AS, Oslo, Norway, L1518-500ML) supplemented with 5% FBS and gentamicin. The next day RNA was isolated from five flasks and used as negative controls (0 h post stimulation samples; 0-hps), six flasks were stimulated with rIFNg at 1 ng/ml for 2 h (2-hps samples) and six flasks were stimulated with rIFNg at 1 ng/ml for 24 h (24-hps samples).

### Cell Lysates

Lysates from one SHK-1 cell flask per time point were used to verify effect of rIFNg. Culture media was removed and cells were washed twice with ice cold PBS supplemented with 1 mM protease inhibitor AEBSF (Merck AS, #A8456). Cells were then lysed in the flask using 0.5 ml lysis buffer (150 mM NaCl, 20 mM Tris-HCl pH 8.0, 1 mM MgCl2, 2% Igepal (Merck AS, #I3021) and 1 mM AEBSF for 10 min (min) on ice. Lysates were incubated on ice with occasional rigorous shaking for 20 min, centrifuged at 13,000 × g for 30 min and supernatants containing the crude lysates transferred to a new Eppendorf tube prior to freezing at minus 20°C.

### Western Blotting and Immunostaining

Twelve μl of the lysates were added to 4x SDS reducing sample buffer prior to denaturing at 80°C for 5 min. Samples were loaded on a BOLT 4–12% Bis-Tris gel (Thermo-Fisher Scientific #NW04125BOX), run for 20 min in 1xMES buffer (Thermo Fisher Scientific, # NP0002). The gel was blotted onto a 0.45 μm Immobilon-P PVDF membrane (Merck AS, Oslo, Norway #IPVH00010) for 70 min at 100 V in a 12.5 mM Tris-Hcl, 96 mM glycine, 20% ethanol solution. The blot was subsequently stained for 1 h in PBS 0.1% tween with undiluted monoclonal supernatant of primary antibodies against UBA (25) or b2m (26), washed three times in PBS 0.1% tween, incubated with 1:1000 dilution of rabbit anti-mouse IgG secondary antibody (Merck AS, #A9044) for 30 min at room temperature and washed three times in PBS 0.1% tween. Staining was visualized using a 1% carbazole (Merck AS, #A5754) solution supplemented with H_2_O_2_ (Merck AS, #H1009).

### RNA Isolation

Cells from five flasks from each time point were washed twice with ice cold PBS, trypsinized using 1 ml 0.25% EDTA-Trypsin and transferred to Eppendorf tubes. Cells were harvested at 2,500 × g for 5 min at 4°C, and resuspended in 350 μl RTL buffer supplied in the RNeasy Mini kit (QIAGEN, #74104). RNA was isolated following the manufacturer's protocol, including a DNAse I treatment step (QIAGEN, #79254) on spin-column. RNA quantity and quality were analyzed using Nanodrop (Thermo Fisher Scientific), Qubit (Thermo Fisher Scientific), and Bioanalyzer (Agilent, Santa Clara, CA, USA) according to manufacturers' protocols. RNA quantity ranged from 78 to 196 ng/μl with absorbance 260/280 ranging from 2.07 to 2.14 and RIN values of 7.0–10.0.

### RNA-seq Library Preparation and Sequencing

RNA-seq libraries were prepared using TruSeq stranded total RNA prep kit (Illumina Norway AS) targeting the poly-A tail to enrich mRNAs. 15 barcoded libraries (5 control; 5 rIFNg stimulated at 2 h time point; 5 rIFNg stimulated at 24 h time point) were pooled together and sequenced over 2 lanes of HiSeq 3000 (Illumina Norway AS) employing 150 bp paired-end sequencing. Library preparation and sequencing was performed at the Norwegian Sequencing Center, Oslo, Norway.

### Illumina Data Analysis

Raw data from two lanes for each sample were concatenated together before data analysis. Adapters and low quality reads were trimmed/removed using BBduk (BBMap v34.56) (27) using “ktrim = *r k* = 23 mink = 11 hdist = 1 tbo tpe qtrim = *r* trimq = 15 maq = 15 minlen = 36 forcetrimright = 149” as parameters. Cleaned reads were aligned against the NCBI Salmon genome (GCF_000233375.1_ICSASG_v2) using hisat2 v2.1.0 (28). Fragments (read pairs) aligning to the 136077 transcripts were counted using featureCounts v1.4.6-p1 (parameters: “-s 2 -p”) (29). Differential expression analysis was carried out using variance stabilizing transformed data using all cleaned reads in DESeq2 v1.22.1 (30) package in R v3.5.1. Significance cut-off was set at adjusted-*p* value (*p*-value adjusted for false discovery rate) of 0.05. Sequence data has been submitted to NCBI Sequence Read Archive (SRA) under the BioProject accession number PRJNA637094.

### GO Term Analysis

Differentially expressed genes with a fold-change of 2 or more were used to perform a gene ontology (GO) analysis using DAVID bioinformatic resources (31), version 6.8. Gene lists were investigated for enrichment against terms in the following GO term lists: GOTERM_BF, GOTERM_MF and INTERPRO, otherwise using default parameters. Data are shown in Supplementary File 4.

### Fragments per Kilobase of Transcript per Million Mapped Reads (FPKM) Calculations

Cleaned RNA-seq reads were aligned to the Sasa-UBA^*^0201 and Sasa-UBA^*^0301 alpha 1 domain nucleotide sequences and the fragments aligning to these gene sequences were counted as above. FPKM values were calculated using the number of fragments aligned, the total number of successfully aligned fragments to the entire transcriptome and the gene length (261 nucleotides) using the standard formula: FPKM = (fragments_aligned ^*^ 10^∧^9)/(total_number_of_successfully_aligned_fragments ^*^ gene_length).

### Data Mining

Both GenBank nucleotide and protein databases were mined for mammalian and teleost gene orthologs. Gene duplications were then identified through tblastn search of the Atlantic salmon genome. Orthology was tested using clustal alignments (32) and phylogenetic analyses using MEGA (33). Gene homeologs were defined based on regional location (17). We use standardized nomenclature for gene duplicates originating from the fourth salmonid-specific 4R-WGD where one homeolog is given the extension –a and the other homeolog is given the extension –b. Some gene duplicates mostly originating from the 3R-WGD have been given the extension –like or –L, while gene copies of unknown location have consecutive numbers.

## Results and Discussion

### Data Generation and Processing

To verify the effect of the rIFNg and select sampling time points for RNA-seq, we first evaluated the expression of MHC class I UBA and b2m protein levels in rIFNg stimulated cells. Salmon head kidney (SHK-1) cells (24) were stimulated with rIFNg for 2 or 24 h and cell lysates were immunoblotted using available monoclonal antibodies (25, 26). We observed little visual increase at 2 h post stimulation (2-hps), but after 24 h post stimulation (24-hps) both UBA as well as b2m protein levels were visually increased compared to negative controls (Supplementary File 1). Thus, we chose to perform the RNA-seq study using 2-hps to detect early response genes and 24-hps to represent later responses.

**Figure 1:**
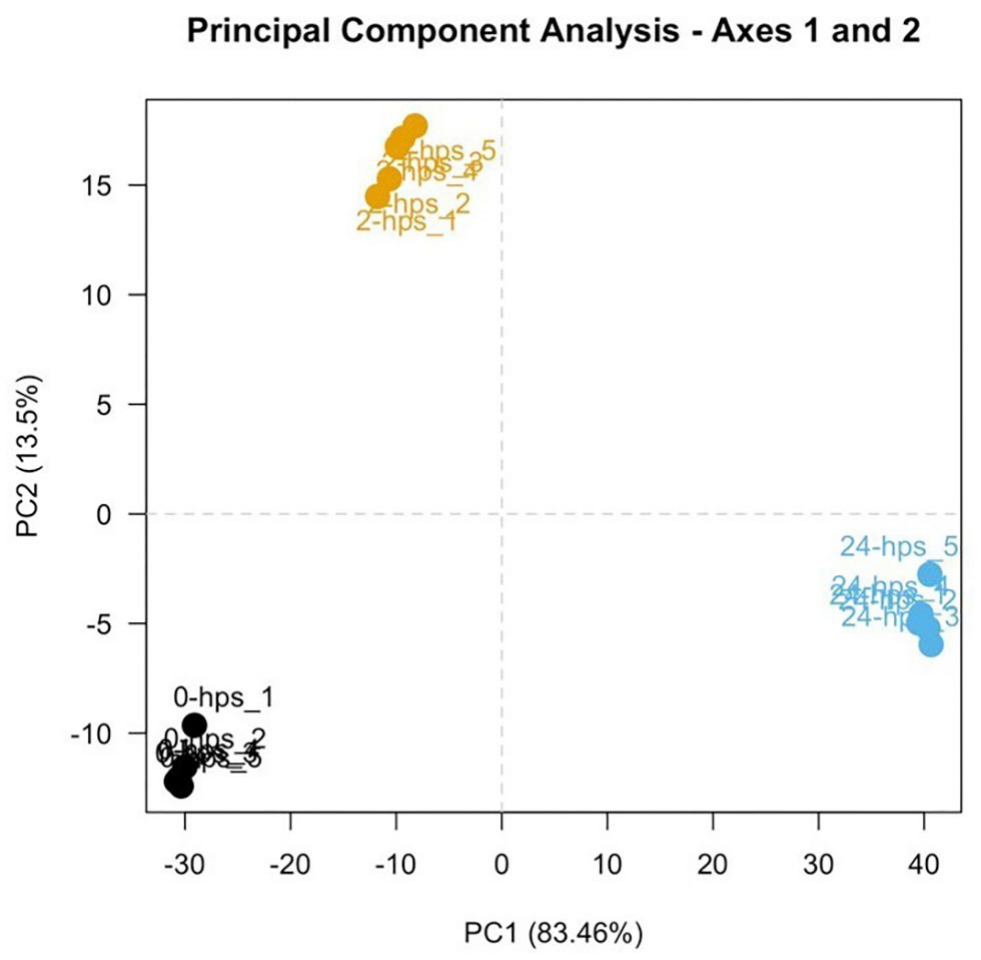
First two components of a Principal Component Analysis, with percentages of variance associated with each axis. Five replicates of 0, 2, and 24-hps are represented in black, yellow, and blue dots, respectively.

RNA-seq generated 66–140 million 150 base pair paired-end reads of which more than 98% (65–139 million) of the reads were retained after trimming and removing low quality bases and reads mapping to the sequencing adapters and PhiX (standard Illumina spike-in used sequencing). 84–92% of the cleaned reads aligned to the Atlantic Salmon genome. Principal Component Analysis (PCA; Figure 1) of the normalized count data (using DESeq2) showed a clear separation between the three groups of samples and grouping within the replicates. This indicates that the biological variability is the main source of variance in the data and the experiment and RNA-seq were performed as expected.

### Differentially Expressed Genes

**Figure 2:**
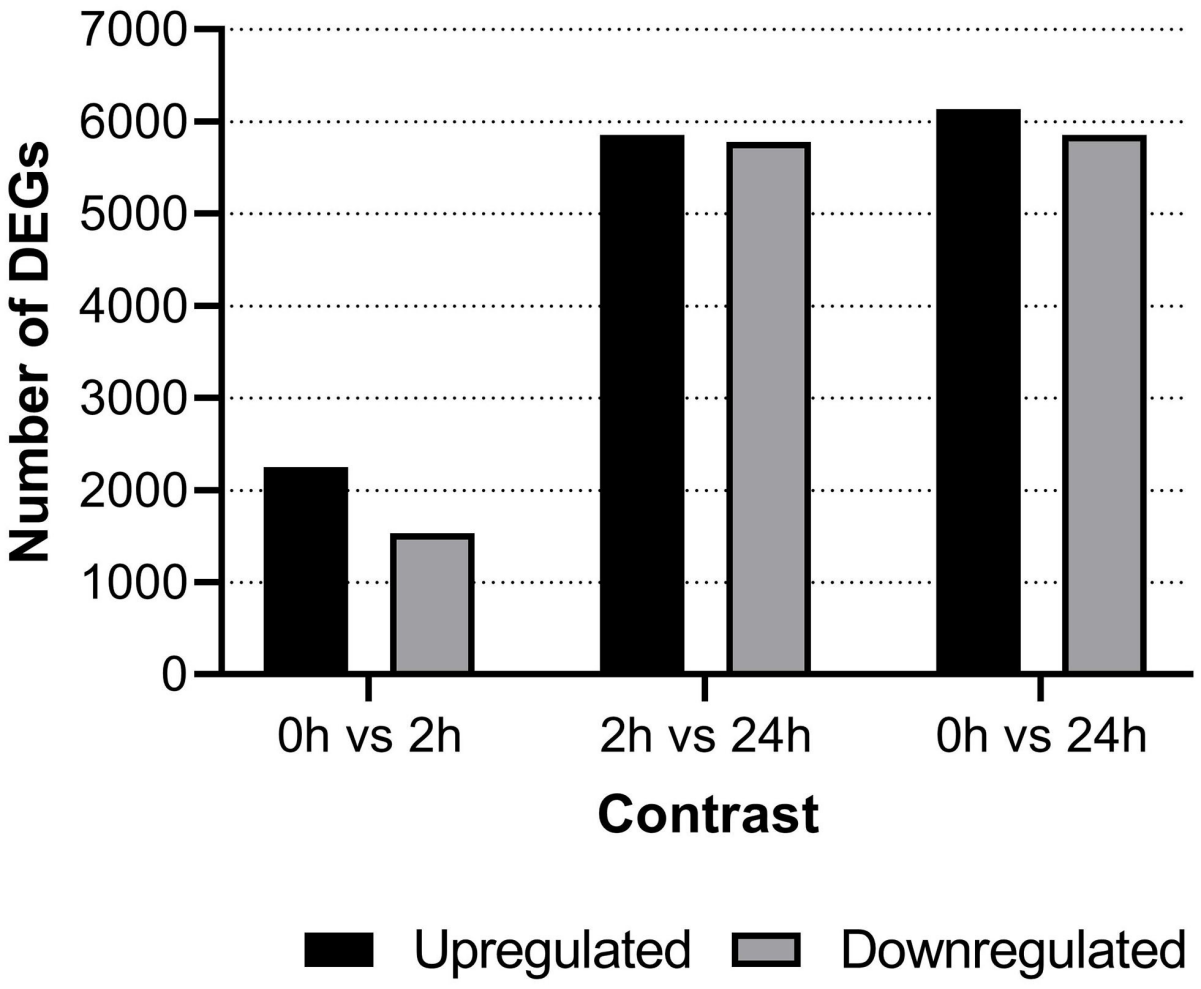
Number of differentially expressed genes shown for each of the three time points i.e., negative control (0-hps) vs. recombinant interferon gamma stimulation for 2 h (2-hps) or 24 h (24-hps). Horizontal line bars show upregulated genes while downregulated genes are shown using diagonal lines.

Differentially expressed genes (DEG) were identified between groups with the cut-off set at a minimum log_2_ fold change difference of one and adjusted *p* < 0.05. At 2-hps, a considerable number of genes were both upregulated as well as downregulated in our material (Figure 2). The major bulk of differential response occurred between 2 and 24-hps with almost 12,000 DEGs. Comprehensive information on all DEGs are compiled in Supplementary File 2 while details of selected genes and gene duplicates are provided in Supplementary File 3.

### Our Cellular Model System

Based on cellular enzyme activity pattern and the ability to internalize inactivated bacteria, SHK-1 cells were originally described as macrophage-like, but with the reservation that their elongated, flattened morphology and lack of bactericidal activity were less consistent with this phenotype (24, 34). Later, a monoclonal antibody raised against lysates of virus-infected SHK-1 cells was found to react stringently with endothelial and red blood cells in Atlantic salmon tissues (35). Thus, we evaluated the expression of endothelial-specific gene transcripts. We found that SHK-1 cells express moderate to high levels of many typical endothelial-enriched genes [CDH5.1, KDRL, DUSP5, PAR1; (36–38); Supplementary File 3], but miniscule amounts of Von Willebrandt factor (VWF).

We here use miniscule expression as below 20 matching reads, low expression as 21–150 matching reads, medium expression as 151–1,000 matching reads and high expression as above 1,000 matching reads. Endothelial cells are a heterogeneous group of cells where expression profiles vary depending on type and tissue origin potentially explaining the lack of VWF (39). Also, extracting ECs from their natural environment and placing them in culture also influence their expression profiles. As shown by the data presented below, the SHK-1 cells do respond as would be expected of endothelial cells upon IFNg induction with strong upregulation of CXCL10, CXCL11 and guanylate-binding protein 1 (GBP1) (40). However, because our current knowledge about transcription profiles of teleost cells is limited, a definite classification of this cell line is currently not possible.

### GO Term Enrichment

A GO analysis confirmed a prototypic response to interferon gamma. The list of genes upregulated 2 hps was enriched for terms associated with transcriptional regulation and interferon-signaling. At 24 hps we also observed enrichement for terms related to the proteasome and cell cycle regulation. Enriched terms for each contrast are detailed in Supplementary File 4.

### Activation of the JAK/ STAT Pathway

An obvious limitation of using the Atlantic salmon genome as a reference for our analysis is the lack of nomenclature discriminating between gene duplicates. Thus, to ensure detection of all duplicated genes, we performed homology search, phylogenies and sequence identity analyses of genes from various Atlantic salmon, other teleost and human gene sequences for each gene of interest (Supplementary Files 5–7). Deduced amino acid sequences of genes of interest are compiled in Supplementary File 5, phylogenetic trees are compiled in Supplementary File 6 and sequence identities are compiled in Supplementary File 7.

We first evaluated the expression and regulation of genes involved in the JAK/STAT pathway. Four JAK1 genes and three JAK2 genes are present in Atlantic salmon (Supplementary Files 5–7). The ssJAK1 and ssJAK1L duplication occurred in an ancestor of pike where other teleosts do not seem to have this gene duplication. The salmonid specific 4R-WGD then duplicated each of these 3R-WGD genes into ssJAK1a and ssJAK1b, and ssJAK1.La and ssJAK1.Lb (Supplementary Files 6, 7). For JAK2, two genes are 4R-WGD homeologs (ssJAK2.1a, ssJAK2.1b) and an additional duplicate was denoted ssJAK2.2. An expected homeolog to the ssJAK2.2 gene seems to have been lost on chromosome 20. In our dataset, the ssJAK1a, ssJAK1.La and ssJAK2.2 genes all have more than 490 matching transcripts at 0-hps, while the remaining four genes are supported by < 120 matching reads (Table 1, Supplementary File 3). Only ssJAK1.La displays a log_2_ fold change above 2 at 24-hps while the other two genes are downregulated at 24-hps. As JAK1 and JAK2 are activated by IFNg through phosphorylation of pre-existing as well as newly synthesized JAK proteins, three of the seven JAK1/2 genes potentially influence downstream signaling in response to rIFNg.

**Table 1:** Log_2_ fold change with adjusted *P*-values for selected transcription factors.

**Gene name**	**2-hps vs. 0-hps**	**24-hps vs. 2-hps**	**24-hps vs. 0-hps**	**Genomic location**
JAK1a	ns	ns	−0.391 (7.63e-10)	NC_027309.1: 41.900.001–41.955.907
JAK1b	0.531 (0.011674)	ns	ns	NC_027322.1:9.336.812–9.396.447
JAK1.La	1.823 (4.30e-69)	0.693 (5.34e-11)	2.516 (1.37e-132)	NC_027313.1:8.762.241–8.786.215
JAK1.Lb	NA	NA	NA	NC_027302.1:9.567.654–9.594.450
JAK2.1a	NA	NA	NA	NC_027300.1:110.012.942–110.189.997
JAK2.1b	ns	0.893 (0.040128)	1.484 (0.000919)	NC_027312.1:96.918.887–97.976.821
JAK2.2	ns	−0.258 (0.000404)	−0.234 (0.001863)	NC_027323.1:26.874.135–26.911.606
STAT1.1a	1.254 (3.90e-05)	ns	ns	NC_027315.1:64.447.884–64.461.742
STAT1.1b	NA	NA	NA	NC_027316.1:7.168.750–7.181.496
STAT1.2a	0.721 (7.62e-27)	2.146 (8.83e-254)	2.867 (0)	NC_027320.1:12.218.693–12.235.684
STAT1.2b	NA	NA	NA	NC_027324.1:10.235.279–10.263.141
STAT2a	0.293 (8.78e-06)	0.366 (1.58e-09)	0.659 (5.39e-29)	NC_027311.1:58.670.995–58.782.875
STAT2b	ns	ns	ns	NC_027321.1:28.174.986–28.181.318
IRF1a	5.392 (0)	−1.197 (3.08e-22)	4.20 (5.53e-233)	NC_027312.1:54.674.198–54.678.695
IRF1b	6.760 (0)	−2.025 (9.28e-91)	4.74 (0)	NC_027303.1:69.623.751–69.628.218
IRF9a	ns	2.721 (3.59e-07)	2.379 (5.40e-06)	NC_027318.1:68.799.550–68.807.766
IRF9b	NA	NA	NA	NC_027328.1:25.025.478–25.031.127
NLRC5a	ns	1.95 (0.000314)	2.58 (1.91e-05)	NC_027309.1:79.263.529–79.290.760
NLRC5b	1.494 (3.07e-28)	2.86 (9.72e-203)	4.36 (1.39e-278)	NC_027315.1:31.288.575–31.311.835
CIITA	ns	2.91 (2.92e-49)	2.32 (4.51e-36)	NW_012347531.1:5.494–24.308
RFXANK	ns	1.56 (3.30e-40)	1.84 (1.57e-50)	NC_027315.1:38.347.885–38.350.459
RFXANK.La	NA	NA	NA	NC_027300:141.219.549–141.228.949
RFXANK.Lb	−0.20 (0.236395)	−0.18 (0.204482)	−0.37 (0.002066)	NC_027310.1:51.185.525–51.190.841
RFXAPa	ns	ns	ns	NC_027303.1:80.495.836–80.499.233
RFXAPb	NA	NA	NA	NC_027310.1:87.363.502–87.371.927
RFX5a	NA	NA	NA	NC_027313.1:60.828.512–60.843.569
RFX5b	NA	NA	NA	NC_027326.1:11.825.491–11.840.434

*Log_2_ fold change for each selected gene is shown with adjusted P-values in parenthesis and chromosomal location. Details including accession numbers for each gene, average number of matching transcripts per time point and chromosome number are gathered in Supplementary File 3, while selected phylogenies and sequence identities are gathered in Supplementary Files 6, 7. NA, not applicable due to lacking data; and ns, not significant*.

Four duplicate STAT1 genes have been reported in rainbow trout (41). Only three of these (STAT1.1a, STAT1.2a and STAT1.2b) are annotated in the Atlantic salmon genome, where the Atlantic salmon chromosome 17 ortholog to the trout STAT1a2 gene may or may not be a pseudogene (Supplementary Files 5–7). Three previously reported Atlantic salmon ssSTAT2 sequences all cluster with our ssSTAT2a sequence suggesting sequence polymorphism in this gene [AHN82335.1, AHN82336.1, ACM43809.1 (42)]. The annotated genome ssSTAT1.2a and ssSTAT2a sequences have truncated 3' regions, but that may or may not be a genome artifact. Moreover, for the ssSTAT1.2a gene, the genome contains more exons than included in the annotated sequence. If the ssSTAT2b gene is one the verge of pseudogenization, thus lacking the dimerization and phosphorylation region, it would match the findings of Dehler and co-workers that salmonids only have one STAT2 gene (41), and also explain the low number of reads found for this gene in our study.

Only ssSTAT1.2a and ssSTAT2a display moderate to high expression levels at all time points, while the remaining four STAT genes only have < 61 matching transcripts (Table 1, Supplementary File 3). ssSTAT1.2a is more affected by rIFNg than the ssSTAT2a gene where ssSTAT1.2a reaches a 2.8 log_2_ fold increase at 24-hps as compared to 0.7 for ssSTAT2a. Although ssSTAT2a has a six times higher 0-hps level than ssSTAT1.2a, it reaches approximately the same number of matching transcripts as ssSTAT1.2a at 24-hps. Thus, one of four ssSTAT1 genes and one of two ssSTAT2 genes respond to rIFNg stimulation with potential effect on downstream JAK/STAT pathway genes. In a chinook salmon cell line, STAT2 knockout did not affect downstream signaling following IFNg stimulation, suggesting that STAT1 operated as a homodimer (41). Instead, STAT2 was essential for the type I interferon pathway.

Following translocation to the nucleus, these phosphorylated STATs induce transcription of a range of genes, amongst them the IRFs (8). Indeed, IRF1 is one of the highest induced genes in our material. 0-hps transcription levels of the 4R-WGD ssIRF1a and ssIRF1b homeologs are limited to a few transcripts, but reach a 5-6 log_2_ fold increase after 2-hps (Table 1, Supplementary Files 3, 5–7). The expression levels of both genes decrease at 24-hps indicating efficient negative regulation. ssIFR1 is thus the major factor affecting downstream responses as duplicate ssIRF9 genes are barely expressed (Supplementary File 3).

### Interferon Stimulated First Line of Defense

The stimulatory effect of interferon gamma needs to be transient and is therefore heavily regulated by suppressor of cytokine signaling 1 (SOCS1), which turns off the JAK phosphorylation in turn reducing the phosphorylation of STATs (43). SOCS1 is also an inhibitor of the IFN signaling pathways in teleosts (44). There are five SOCS1 duplicates in the Atlantic salmon genome, all residing on unplaced scaffolds (Supplementary Files 3, 5). Three of these duplicates are heavily induced in our dataset (SOCS1.1-1.3), while the remaining two duplicates show no expression (SOCS1.5 and SOCS1.6) (Table 2, Supplementary Files 3, 5–7). As expected, expression of SOCS1 genes peak early after IFNg stimulation, with a 5-6 log_2_ fold increase at 2-hps with a decline to log_2_ fold increase of 4 at 24-hps.

**Table 2:** Log_2_ fold change with adjusted *P*-values for selected first line of defense genes.

**Gene name**	**2-hps vs. 0-hps**	**24-hps vs. 2-hps**	**24-hps vs. 0-hps**	**Genomic location**
SOCS1.1	5.77 (5.89e-102)	−1.85 (1.03e-18)	3.94 (1.55e-45)	NW_012416168.1:784–1.683
SOCS1.2	5.78 (2.88e-149)	−1.87 (1.24e-37)	3.92 (2.85e-65)	NW_012358293.1:6.016–7.112
SOCS1.3	6.32 (0)	−2.11 (2.27e-81)	4.22 (1.20e-201)	NW_012347411.1:23.097–27.026
SOCS1.4	ns	ns	4.50 (0.0011674)	NW_012426344.1:13–1.027
SOCS1.5	NA	NA	NA	NW_012349686.1:7.664-9.765
CK10a	ns	4.56(5.20e-05)	4.95 (1.29e-05)	NC_027314.1:3.252.135–3.256.106
CK10b	0.87 (1.68e-14)	5.531 (0)	6.40 (0)	NC_027323.1:41.009.563–41.020.152
CK13a	7.13 (2.46e-91)	1.47 (8.94e-29)	8.60 (5.05e-134)	NC_027310.1:71.597.209–71.598.608
CK13b	6.18 (0)	−0.70 (2.18e-07)	5.48 (0)	NC_027300.1:122.209.795–122.211.056
CXCL11L1.1	7.15 (2.70e-103)	−1.05 (1.83e-08)	6.11 (2.50e-75)	NC_027314.1:3.502.962–3.504.253
CXCL11L1.2	6.78 (0)	−0.46 (0.013694)	6.31 (2.73e-286)	NC_027314.1:3.514.954–3.517.032
CXCL11.L1.3	7.28 (0)	−0.89 (9.47e-11)	6.39 (1.54e-236)	NC_027314.1:3.545.565–3.547.816
MX1	0.69 (1.15e-06)	1.29 (3.07e-23)	1.98 (5.19e-52)	NC_027311.1:66.798.392–66.803.823
MX2	ns	3.50 (0.008916)	ns	NC_027311.1:66.776.321–66.785.731
MX3	0.21 (0.000505)	1.18 (1.63e-84)	1.39 (7.68e-116)	NC_027311.1:66.816.619–66.828.660
MX4	3.09 (4.26e-54)	1.77 (9.76e-20)	4.86 (7.22e-130)	NC_027324.1:47.104.856–47.121.653
MX5	ns	4.59 (3.07e-23)	6.77 (8.06e-14)	NC_027324.1:47.217.828–47.228.438
MX6	ns	3.45 (1.22e-07)	4.28 (1.44e-07)	NC_027324.1:47.139.133–47.161.993
MX7	ns	4.11 (4.19e-07)	3.91 (1.39e-06)	NC_027324.1:47.175.786–47.193.273
MX8	3.13 (1.78e-67)	4.45 (0)	7.57 (0)	NC_027324.1:47.243.603–47.262.617
MX9	ns	ns	ns	NC_027308.1:117.838.751–117.853.817
MX10	NA	NA	NA	NC_027314.1:5.292.440–5.299.092
IFI44.1	0.43 (0.000524)	2.69 (1.40e-98)	3.00 (3.57e-132)	NC_027313.1:62.942.467–62.979.179
IFI44.2	0.27 (0.108220)	2.85 (1.04e-139)	3.12 (1.38e-165)	NC_027313.1:63.251.718–63.254.829
IFI44.3	ns	ns	ns	NC_027316.1:9.105.5499.110.942
IFI44.4	NA	NA	NA	NC_027315.1:43.549.791–43.558.484
IFI44.5	ns	ns	ns	NC_027315.1:43.616.730–43.627.930
IFI44.6	0.27 (0.000428)	0.74 (5.70e-22)	1.01 (1.07e-38)	NC_027315.1:43.668.157–43.675.309
IFI44.L1a	7.77 (1.36e-31)	8.19 (0)	15.96 (9.54e-126)	NC_027311.1:44.185.618–44.193.898
IFI44.L1b1	7.12 (1.14e-14)	ns	6.94 (6.76e-13)	NC_027321.1:42.904.844-42.926.820
IFI44.L1b2	5.24 (1.02e-40)	5.62 (4.61e-222)	10.86 (1.88e-169)	NC_027321.1:42.975.136–42.984.875
IFI44.L1b3	3.26 (1.51e-43)	4.03 (2.06e-115)	7.29 (7.02e-216)	NC_027321.1:43.039.358–43.066.852
IFI44.L1b4	1.99 (1.51e-40)	2.52 (4.64e-72)	4.51 (5.72e-203)	NC_027321.1:43.104.110–43.110.856
IFI44.L2	NA	NA	NA	NC_027305.1:19.840.801–19.856.156

*Log_2_ fold change for each selected gene is shown with adjusted P-values in parenthesis and chromosomal location. Details including accession numbers for each gene, average number of matching transcripts per time point and chromosome number is gathered in Supplementary File 3, while selected phylogenies and sequence identities are gathered in Supplementary Files 6, 7. NA, not applicable due to lacking data; and ns, not significant*.

IFNg is produced by activated T cells, NKT cells and epithelial cells, amongst other in response to viral infection, and activates anti-viral genes through the JAK/ STAT pathway discussed above. One such viral resistance gene is the Myxovirus resistance (MX) gene, of which Atlantic salmon has 10 gene copies as opposed to the two MX genes in the human genome (45) (Supplementary Files 5–7). Eight duplicates are induced by interferon gamma in our material, ranging from 1.4 to 7.6 log_2_ fold increase with ssMX8 being the most affected gene (Table 2, Supplementary File 3). This is in line with results from a previous study (45), where ssMX8 also was strongest regulated by rIFNG stimulation as opposed to MX1-3 genes that displayed a stronger response to type I IFN. Also two rainbow trout sequences clustering with this ssMX8 sequence (onmy-MX5 and onmy-MX6, Supplementary File 6), are more stimulated by IFNg than IFNa (Supplementary File 6) (46).

IFNg stimulation also stimulates the production of chemokines that attract immune cells to infected sites. Three Atlantic salmon chemokines all denoted CCL19-like in the genome, belonging to the teleost CCL19/21/25 clade clustering with human CCL19, CCL21 and CCL25 sequences (47, 48), are highly induced in our dataset (Table 2, Supplementary Files 3, 5–7). The CC chemokine we named ssCK13b peaked with a log_2_ fold induction of 6.2 at 2-hps. Its 4R-WGD homeolog ssCK13a does not peak at 2-hps, but continues to a log_2_ fold increase of 8 at 24-hps. The third CC chemokine, called ssCK10 (47), is a single copy gene and reaches a log_2_ fold induction of 6.4 at 24-hps. Which cell types the CK13 chemokines attract remains unknown, but a teleost CK10 ortholog (CCL19-like; AVH76855.1) was shown to attract lymphocytes and monocytes/macrophages, with no significant effect on neutrophil migration (48).

In humans, the IFNg induced CXC chemokines CXCL9-11 mediate recruitment of T cells, natural killer cells and monocytes/macrophages to the infection site (49). A trout CXCL11_L1 gene (alias γIP-10) has previously been shown to be interferon gamma inducible with ISRE and GAS elements in its promoter (50–52). In zebrafish, CXCL11 was also shown to attract macrophages in zebrafish embryos through CXCR3 signaling (53). In our model system, the three regionally duplicated CXC chemokines ssCXCL11_L1.1, ssCXCL11_L1.2, and ssCXCL11_L1.3, all peaked with log_2_ fold increased transcription of 7 at 2-hps while induction levels decreased at 24-hps (Table 2, Supplementary Files 3, 5–7).

Induction of MX, chemokines and SOCS are all expected following IFNg stimulation and validate a biological function for our rIFNg. A gene that has not been studied extensively in relation to IFNg stimulation is interferon induced protein 44 (IFI44). In humans, IFI44 has been reported to be induced by interferon I but not interferon gamma (54). Its exact function remains unresolved, but in mammals it has both been reported to support as well as suppress viral replication (55, 56). In teleosts, IFI44 has diversified to 20 IFI44 and IFI44-like (IFI44.L) genes in zebrafish (57) and 12 IFI44 and IFI44.L genes in Atlantic salmon (Supplementary Files 5–7). The two duplicated human IFI44 and IFI44.L genes are orthologs of the teleost IFI44 gene, and not the IFI44.L genes. Spotted gar, a species that only experienced two WGDs, also has an expansion with at least 11 IFI44 genes, but no detectable IFI44.L genes (data not shown). The IFI44.L genes thus seem to have emerged in a basal teleost resulting from the teleost specific 3R-WGD.

IFI44.L genes showed the highest log_2_ fold change of all genes in our study (Table 2, Supplementary File 3). The ssIFI44.L1a gene on chromosome 12 has only a few transcripts at time zero, but reaches a log_2_ fold increase of 16 at 24-hps. The four regionally duplicated ssIFI44.L1 genes on chromosome 22 (IFI44.L1b1-b4) are also highly upregulated with log_2_ fold increase of 4.5-10.9 at 24-hps. In comparison, three of the six ssIFI44 genes display moderate log_2_ fold increase of 1–3 at 24-hps. In zebrafish, unfortunately only one of the ten IFI44.L genes (ENSDARG00000010729) were included on the microarray used, displaying a log_2_ fold increase of 3 upon virus infection (57). The extreme induction of IFI44.L in Atlantic salmon calls for further studies on the biological role of these IFI44.L genes in teleosts.

Other typical IFNg inducible genes are also affected by rIFNg in our material, including the fourteen interferon-induced guanylate-binding protein 1 (GBP1) genes, where the highest induced gene duplicate reached a log_2_ fold increased transcription of 5.3 at 24-hps (Supplementary File 3).

### MHC Class I Pathway Genes

The induced protein levels of the classical MHCI gene UBA at 24-hps used to verify the biological function of our recombinant IFNg, was reflected in the transcript levels. UBA is highly transcribed at 0-hps, but still has a log_2_ fold increase of 2.3 at 24-hps (Table 3, Supplementary File 3). A few non-classical MHCI genes also have log_2_ fold values above 2, including the U lineage gene ULA and the L lineage genes LGA and LIA, although both their 0-hps and also 24-hps transcript levels are much lower than the UBA locus.

**Table 3:** Log_2_ fold change with adjusted *P*-values for selected MHCI pathway genes.

**Gene name**	**2-hps vs. 0-hps**	**24-hps vs. 2-hps**	**24-hps vs. 0-hps**	**Genomic location**
TAP2a	1.98 (1.21e-162)	1.58 (2.41e-113)	3.56 (0)	NC_027326.1:10.176.035–10.180.872
TAP2b	1.63 (1.41e-13)	3.57 (1.61e-140)	5.20 (1.92e-161)	NC_027313.1:50.968.347–50.981.043
TAP2c	ns	2.25 (1.98e-45)	2.64 (6.20e-52)	NC_027318.1:51.232.532–51.213.314
TAP1	0.56 (5.71e-10)	2.01 (2.02e-112)	2.57 (1.60e-180)	NC_027304.1: 55.587.009–55.516.067
TAPBPa	2.36 (7.10e-85)	1.60 (6.20e-44)	3.95 (7.30e-243)	NC_027326.1:10.023.358–10.039.557
TAPBPb	ns	ns	ns	NC_027313.1:59.041.175–59.053.861
TAPBPc	ns	ns	ns	NC_027313.1:50.968.346-50.981.042
TAPBPL1a	0.38 (0.033319)	1.29 (1.02e-22)	1.67 (1.10e-36)	NC_027308.1:102.783.830–102.789.947
TAPBPL1b	0.22 (0.003439)	1.31 (1.38e-113)	1.53 (1.81e-149)	NC_027319.1:46.797.073–46.803.184
TAPBPL2	0.89 (3.91e-35)	2.15 (9.43e-240)	3.04 (0)	NC_027306.1:12.926.171–12.930.490
PSMB8a	ns	3.00 (1.43e-08)	2.73 (1.74e-07)	NC_027326.1:10.153.896–10.157.742
PSMB8b	0.50 (8.94e-07)	2.51 (2.26e-204)	3.01 (1.17e-267)	NC_027313.1:59.095.252–59.098.375
PSMB9a	ns	1.15 (9.04e-07)	1.78 (1.58e-12)	NC_027326.1:10.171.104–10.175.148
PSMB9b	0.24 (0.01314)	2.21 (9.86e-226)	2.45 (3.44e-261)	NC_027313.1:59.112.113–59.116.449
PSMB10a	ns	1.76 (8.81e-67)	2.00 (2.38e-85)	NC_027326.1:10.580.777–10.585.553
PSMB10b	ns	1.80 (7.91e-122)	2.01 (1.56e-148)	NC_027313.1: 59.678.647–59.684.589
PSMB12a	0.39 (0.00013)	1.36 (9.22e-57)	1.75 (1.26e-91)	NC_027326.1:10.163.427–10.168.543
PSMB12b	ns	1.98 (8.70e-96)	1.90 (8.52e-88)	NC_027313.1:59.103.305–59.111.118
PSMB13a	1.19 (1.46e-22)	1.08 (1.85e-24)	2.26 (2.49e-86)	NC_027326.1:10.158.350–10.163.366
PSMB13b	0.49 (5.57e-11)	2.35 (0)	2.84 (0)	NC_027313.1:59.099.410–59.103.327
PSME1a	ns	1.15 (1.02e-12)	1.29 (8.84e-16)	NC_027328.1: 20.102.332–20.105.959
PSME1b	ns	1.60 (2.75e-31)	1.83 (2.50e-40)	NC_027318.1: 74.398.814–74.401.718
PSME2a	ns	1.24 (3.30e-25)	1.41 (1.31e-32)	NC_027328.1: 17.457.855–17.464.336
PSME2b	ns	1.82 (7.14e-62)	2.03 (6.38e-76)	NC_027318.1: 77.929.964–77.936.657
ERAP1	ns	2.29 (2.39e-165)	2.35 (9.85e-174)	NC_027314.1:2.523.971–2.545.383
ERAP2a	ns	1.93 (8.10e-56)	2.01 (1.62e-60)	NC_027323.1:15.056.362–15.064.968
ERAP2b	ns	ns	ns	NC_027319.1:28.632.876–28.646.665
b2m1	ns	1.39 (1.07e-21)	1.66 (8.37e-31)	NC_027306.1:57.825.509–57.828.543
b2m2	ns	1.50 (9.16e-11)	1.41 (1.75e-09)	NC_027306.1:58.301.914-58.303.127
b2m3	ns	2.29 (0.039573)	2.43 (0.037624)	NW_012366138.1:15–1.215
b2m4	ns	ns	2.05 (0.000570)	NW_012360375.1:3.156–4.398
b2m5	ns	1.39 (7.24e-14)	1.54 (2.88e-16)	NW_012376632.1:2.522–3.764
b2m6	ns	0.41 (2.70e-07)	0.45 (1.22e-08)	NW_012349147.1:6.171–7.264
b2m7	ns	1.45 (5.98e-53)	1.59 (2.50e-62)	NW_012349147.1:9.889–11.950
b2m8	ns	0.94 (1.67e-38)	1.02 (5.36e-45)	NC_027316.1:57.628.552–57.632.032
b2m9	ns	0.50 (1.30e-14)	0.39 (3.15e-09)	NW_012347820.1:16.383–20.477
b2m10	ns	1.02 (2.39e-05)	1.20 (6.06e-07)	NW_012353146.1:474–1.303
b2m11	ns	1.00 (4.70e-05)	1.27 (4.73e-07)	NW_012394841.1:962–1.728
b2m12	ns	0.87 (0.013832)	ns	NW_012394112.1:459–1.277
UBA*0301	0.46 (4.53e-05)	1.83 (1.14e-77)	2.28 (2.44e-121)	NC_027326:10.122.009–10.149.394

*Log_2_ fold change for each gene is shown with adjusted P-values in parenthesis and chromosomal location. Details including accession numbers for each gene, average number of matching transcripts per time point and chromosome number is gathered in Supplementary File 3, while selected phylogenies and sequence identities are gathered in Supplementary Files 6, 7. NA, not applicable due to lacking data; and ns, not significant*.

The canonical W/S, X2 and Y/Enhancer B promoter motifs known to bind the mammalian MHCI and b2m enhanceosome (4), are also found in many teleost MHC class I promoter sequences (20, 21). In our study, only a few of these Atlantic salmon transcription factor duplicates, i.e., ssRFXAPa and ssRFXANK display a moderate rIFNg upregulation at 24-hps (Table 1, Supplementary File 3). In humans, the IFNg stimulated master regulator NLRC5 controls the SXY induced transcription of many antigen presentation pathway genes including MHC class I and b2m (4, 58). The Atlantic salmon ssNLRC5b displays a log_2_ fold increase of 4.4 at 24-hps, while its 4R-WGD homeolog is barely transcribed. We find no transcripts for the duplicate ssRFX5 homeologs and only low transcript levels for one of the nine ssNFYA/B/C subunit genes (ssNF-YB.1a, Supplementary File 3), suggesting the enhanceosome does not affect IFNg induced transcription of antigen presenting genes in this model system. This is opposed to the situation in rats, where lack of RFX5 severely impairs MHCI expression (6). Various mammalian MHCI genes can use other transcription factors such as STATs, IRFs, SP1, USFs, AP1 and NF-kβ (59, 60). In our model system, we find no matching reads for the single SP1 gene and only low transcript levels for one of the seven USF1 and USF2 genes (USF2.2a) (Supplementary File 3). For the NF- kβ family, five of eight genes display moderate to high expression levels (NF- kβ_p65a, NF- kβ_p65b2 and NF- kβ_p105b; Supplementary File 3).

Without a functional enhanceosome, ssSTAT1 and ssIRF1 transcription factors seem likely candidates for the UBA induction (Table 1, Supplementary File 3). Multiple sites for STAT/IRF1 transcription factor binding are present in Atlantic salmon and rainbow trout UBA promoters with both GAS and ISRE elements (20, 21). A similar IFNg induction of MHCI by STAT1 and IRF1 has been reported in humans (8, 61).

The SHK-1 cells are heterozygous for the Sasa-UBA^*^0201 and Sasa-UBA^*^0301 alleles (21). However, alignment to the completely homozygous Atlantic salmon genome will only list reads matching the Sasa-UBA^*^0301 allele. The two SHK-1 UBA alleles have 65 percent sequence identity in their alpha 1 domain, but 100 percent sequence identity in the alpha 2 and downstream regions. Thus, the majority of matching reads would not discriminate between alleles. To identify expression levels for each allele we calculated the FPKM values using the alpha 1 domain sequences only. As seen in the genome based DEG analysis, the transcript levels of both alleles were substantial even at 0-hps with FPKM values of 536 and 1,057 for the UBA^*^0301 and UBA^*^0201 alleles, respectively. Both alleles were equally induced at 24-hps reaching high average FPKM values of 2,191 and 4,440 for the UBA^*^0301 and UBA^*^0201 alleles, respectively. Similarly, high basal levels of MHCI transcripts in unstimulated cells were also found in mouse lymphatic endothelial cells (62).

Following transcription and translation, newly synthesized UBA molecules need assistance by CALR and protein disulfide isomerases (ERP57 alias PDIA3) to achieve proper folding. This gene is present in six copies in Atlantic salmon originating from the 2R-, 3R-, and 4R-WGD [Supplementary File 6 (16)], but none are largely affected by rIFNg. ssERP57 and ssERP57L genes originate from the 2R-WGD, ssERP57L1 and ssEP57L2 are remnants of the 3R-WGD while the salmonid specific 4R-WGD duplicated the ssERP57 and ssERP57L2 genes (16). ERP57a and ERP57b are highly expressed in normal tissues as well as in untreated SHK-1 cells (Supplementary File 3). None of these five genes display a log_2_ fold change above 1.3 (Supplementary File 3).

Atlantic salmon b2m sequences originating from 12 different loci, segregate into two distinct clades here defined as the BA1 (ssb2m1-5) and the BA6 clades (ssb2m6-12) (Supplementary Files 5–7). Due to high b2m sequence identity between individual gene sequences, it is problematic to match reads to individual transcripts. Instead, we compared transcription levels of the two clades. The BA6 clade has four times more transcripts at time zero, but only reaches a log_2_ fold increase of 0.65 at 24-hps as compared to the 1.66 log_2_ fold induction of sequences from the BA1 clade (Table 3, Supplementary File 3).

We assume that most MHCI lineage genes are non-covalently associated with b2m, but we do not know if the two b2m clades represent molecules with functional preference for different MHCI molecules. Looking at the b2m promoters, the dual b2m clustering is also apparent in the promoter regions (Supplementary File 5). ssb2m1-5 and ssb2m7 have 930 or more completely identical nucleotide sequences upstream of the start codon and should thus respond equally to IFNg stimulation. b2m8, b2m11, and b2m12 also share 500 bp of identical promoter nucleotide sequence upstream of the start codon and should also mount similar responses to stimulation. Nevertheless, distant transcription factor elements and enhancers may also influence transcription, possibly explaining the differences in IFNg induction observed in our data set.

In mammals, IFNg stimulates replacement of the constitutive proteasome subunits PSMB5-PSMB7 by the subunits PSMB8-10 representing the immunoproteasome (9). This shift in proteasome components produces peptides preferably with hydrophobic or positively charged residues at the C terminus, which are optimally suited for binding to the antigen transporter TAP and to MHC Ia molecules. In addition to the PSMB8 subunit, teleosts have an additional unique PSMB9 subunit denoted PSMB12 as well as an additional PSMB10 subunit denoted PSMB13 (63). In Atlantic salmon, the PSMB8-13 subunit genes all reside as copies in both duplicate MHCI regions on chromosome 14 and 27 (16) (Supplementary File 8). All these duplicate Atlantic salmon PSMB subunits display a log_2_ fold increased expression level of 1.8–3.0 at 24-hps with ssPSMB8b and ssPSMB13b being most affected (Table 3, Supplementary File 3). Overall, slightly higher transcript levels are seen for ssPSMB subunits from the Ib region than those linked to the UBA gene on chromosome 27. Sequence identity between –a and –b duplicates is 92–100% (Supplementary File 7), increasing the risk of individual reads being matched to the wrong gene duplicate. In contrast, the sequence identity between the ssPSMB9 vs. ssPSMB12 and ssPSMB10 vs. ssPSMB13 is 48–58%, thus there is minimal risk of errors in matching reads. As opposed to zebrafish and chicken (63, 64), no functional MHCI haplotypes exist in Atlantic salmon, displaying no allelic variation in the regionally linked genes (16).

The functional roles of the dual PSMB9 vs. PSMB12 and PSMB10 vs. PSMB13 molecules in teleosts are unclear, and the Atlantic salmon duplicated -a and –b versions of these four molecules adds another layer of complexity. In mammals, there are intermediates between the standard proteasome complex and the immunoproteasome with varying number of IFNg induced subunits (65), suggesting this could also be the case for teleosts. Assuming the different ssPSMB9 vs. ssPSMB12 and ssPSMB10 vs. ssPSMB13 subunits are inter-replaceable, Atlantic salmon has 32 alternative (immune)proteasomes. Evaluating the transcription and induction of these molecules, only ssPSMB8a and ssPSMB9a genes display low expression levels even at 24-hps (Table 3, Supplementary File 3), potentially reducing the number of different (immune)proteasome combinations to 12.

Miniscule number of reads in our data sets match the single ssPSMB7 gene (Supplementary File 3), which is peculiar, as the protein encoded by this gene is a component of the constitutive proteasome One possibility is that the interferon-inducible duplicate ssPSMB10 replaces the ssPSMB7 subunit also in the constitutive proteasome in Atlantic salmon. The substrate binding pockets of PSMB7 and PSMB10 in humans and zebrafish are essentially identical- showing the same trypsin-like activity (66, 67). However, ESTs matching the ssPSMB7 subunit are reported in GenBank (e.g., EG833924.1), so low transcription may be unique to SHK-1 cells.

Examining the other proteasome subunits, the majority have a log_2_ fold increase below 1.2 with the exception of ssPSMA6.2a gene, with a log_2_ fold value of 2.9 at 24-hps (Supplementary File 3). The functional relevance of such an induction cannot be determined based on transcriptional analysis alone. Three other transcribed ssPSMA6 genes exist, displaying a high number of matching transcripts at all measured time points. Thus, if and how the ssPSMA6.2a subunit induction affects the immunoproteasome is unclear. One of the ssPSMA6 subunits has been reported to be downregulated in SHK-1 cells following rIFNg treatment for 24 h, but the authors used a proteome approach that does not discriminate between duplicate genes (68). Additional regulatory mechanisms could result in only some of these ssPSMB transcripts being translated into functional proteins available for the immunoproteasome, however, this hypothesis would need to be validated by further experimentation.

Also the mammalian regulatory proteasome subunits PSME1 and PSME2 are upregulated by IFNg (69). In Atlantic salmon, there are 4R-WGD homeolog genes for the ssPSME1, ssPSME2 and ssPSME3 subunits with an additional third copy of the ssPSME3 gene (Supplementary File 5). ssPSME1b and ssPSME2b respond in a similar manner as their mammalian counterparts with log_2_ fold increase of 1.8 and 2.0 at 24-hps, respectively (Table 3, Supplementary File 3). The remaining PSME subunits display a log_2_ fold increase of 0.4–1.4.

Once peptides are generated by the (immuno)-proteasome, they are transported into the ER by the TAP1/TAP2 channel where both subunits originate from IFNg inducible genes in humans (70). There are three ssTAP2 genes and one ssTAP1 gene in Atlantic salmon. ssTAP2a and ssTAP2b reside closely linked to the U-lineage genes on chromosome 27 and 14 (Supplementary Files 3, 7, 8), while the gene denoted ssTAP2c resides 9 Mb upstream on chromosome 14 and the ssTAP1 genes resides on chromosome 5. The ssTAP1 gene displays a log_2_ fold induction of 2.6 at 24-hps (Table 3, Supplementary File 3).

Just looking at fold increase, the ssTAP2a gene linked to UBA on chromosome 27 has a log_2_ fold increase of 3.6 at 24-hps while the ssTAP2b gene on chromosome 14 has a log_2_ fold increase of 5.2 (Table 3). However, we find 18 times more transcripts for ssTAP2a than for ssTAP2b (Supplementary File 3). Assuming that all these transcripts are translated into proteins with similar efficiency, our data suggest that ssTAP2a may have a more prominent role in transporting peptides than ssTAP2b. Although upregulated, the ssTAP2c gene has fewer matching transcripts, but with a 50 % sequence identity to the other two TAP2s (Supplementary File 7), its function remains unknown. A previous study demonstrated no polymorphism in Atlantic salmon ssTAP2s (16) as opposed to what is found in zebrafish and chicken (63, 71). One could speculate that presence of duplicate homeolog genes has eliminated the need for such allelic diversity.

A study of IFNg responsive elements in rainbow trout TAP2 and PSMB9a promoters showed that the PSMB9a gene required both ISRE as well as GAS elements to respond to stimulation (51). The rainbow trout TAP2 gene on the other hand only required the ISRE element to respond. As IRF1 binds to ISRE elements and STATs bind to GAS elements (8), STAT1 and IRF1 seem to be master regulators of antigen processing and transport in salmonids.

The MHCI molecule, awaiting peptides in the ER lumen, is linked to the TAP1/TAP2 channel by TAPBPs. We previously reported six ssTAPBP and ssTAPBP-like (TAPBPL) genes in addition to one ssTAPBP-related (TAPBPR) gene in Atlantic salmon (16) (Supplementary File 7). We find that the UBA linked ssTAPBPa on chromosome 27 has a log_2_ fold increase of 4 at 24-hps as compared to the duplicate ssTAPBPb gene on chromosome 14 that has miniscule number of matching transcripts (Table 3, Supplementary File 3). This is similar to what is found in normal tissues where the ssTAPBPa gene is expressed ~10 times more than the ssTAPBPb gene (16). An additional ssTAPBPc duplicate on chromosome 14, located 9 Mb upstream closely linked to a non-classical MHCI locus, shows no transcripts. As opposed to that found in chicken (64), Atlantic salmon ssTAPBP genes are not polymorphic (16).

The recently discovered TAPBPL sequences are also present in marsupials and birds, but lost en route to humans (16). The 4R-WGD duplicates ssTAPBPL1a and ssTAPBPL1b show a log_2_ fold increase of 1.5-1.7 at 24-hps while the ssTAPBPL2 gene has a log_2_ fold increase of 3 (Table 3, Supplementary File 3). As in normal tissues, ssTAPBPL1b displayed the overall highest expression levels in unstimulated cells, but is outnumbered by TAPBPL2 at 24-hps (16). The TAPBPL genes have unknown functions, but share low sequence similarity with both ssTAPBP and ssTAPBPR sequences (Supplementary File 7). As there are no ssTAPBPR transcripts in our study material, the ssTAPBPL sequences may hold a similar function in our model system. In normal tissues, the ssTAPBPR ssTAPBPL1b gene have similar transcript levels (16). Future studies are needed to clarify the function of TAPBPL molecules in teleosts as well as in other vertebrates.

Following peptide loading by the TAP/TAPBP complex, MHCI peptides are further trimmed by the ERAP1 and ERAP2 molecules, both IFNg inducible genes (72). In mammals, the ERAP1 and ERAP2 molecules have different specificities, where a heterodimer creates a complex with superior peptide-trimming efficiency (73). The ssERAP1 and ssERAP2a genes both respond with log_2_ fold increase of 2.0-2.4 with four times more ssERAP1 transcripts than ssERAP2a (Table 3, Supplementary File 3). The additional ssERAP2b gene displayed no transcription in our material, but is expressed in normal Atlantic salmon tissues (16).

### MHC Class II Pathway Genes

Despite not generally being regarded as antigen presenting cells, some endothelial cells also have IFNg inducible MHC class II (74, 75) although at a much lower level than MHC class I at least in lymphatic endothelial cells (62). In SHK-1 cells, the single classical MHC class II alpha (DAA) and MHCII beta (DAB) genes (76) were induced by rIFNg, but their transcript levels were 100 times lower than that seen for the classical MHCI gene UBA (Table 4, Supplementary File 3). One of the duplicate MHCII chaperone Invariant chain (Ii) genes displayed a similar transcript and induction level.

**Table 4:** Log_2_ fold change with adjusted *P*-values for selected MHCII pathway genes.

**Gene name**	**2-hps vs. 0-hps**	**24-hps vs. 2-hps**	**24-hps vs. 0-hps**	**Genomic location**
DAB^*^0101	ns	1.74 (1.94e-51)	1.84 (4.94e-54)	NC_027311.1:61.693.946–61.699.456
DAA^*^1201	ns	1.83 (1.72e-35)	1.87 (3.98e-36)	NC_027311.1:61.701.374–61.703.966
Ii.a	NA	NA	NA	NC_027304.1: 37.021.326–37.026.612
Ii.b	ns	1.87 (2.02e-33)	1.54 (2.52e-23)	NC_027308.1:68.289.623–68.299.513
CTSKa	ns	0.94 (6.20e-10)	0.71 (5.07e-06)	NC_027301.1:23.783.794–23.795.107
CTSKb	−0.38 (0.048150)	ns	−0.66 (1.57e-05)	NC_027304.1:56.864.904–56.871.943
CTSL1a1	ns	ns	ns	NC_027300.1:113.265.513–113.267.831
CTSL1a2	ns	ns	ns	NC_027300.1:113.270.838–113.273.816
CTSL1a3	ns	ns	ns	NC_027300.1:113.282.847–113.288.939)
CTSL1a4	ns	ns	ns	NC_027300.1:113.340.522–113.345.645
CTSL1a5	ns	ns	ns	NC_027300.1:113.367.197–113.372.320
CTSL1a6	NA	NA	NA	NC_027300.1:113.380.498–113.388.445
CTSL1b	−0.12 (0.209656)	−0.45 (8.04e-13)	−0.57 (4.98e-20)	NC_027312.1:93.533.493–93.537.645
CTSL2a	ns	0.42 (0.034477)	0.42 (0.035154)	NC_027300.1:10.068.398–10.074.289
CTSL2b	ns	ns	ns	NC_027308.1:7.731.484–7.781.352
CTSL3	NA	NA	NA	NC_027311.1:28.445.519-28.463.099
CTSL4	NA	NA	NA	NC_027322.1:28.904.769–28.907.274
CTSS1a	ns	1.65 (1.30e-84)	1.62 (3.98e-81)	NC_027301.1:23.775.018–23.782.931
CTSS1b	ns	3.45 (7.31e-78)	3.20 (9.01e-69)	NC_027304.1:56.872.975–56.879.886

*Log_2_ fold change for each gene is shown with adjusted P-values in parenthesis and chromosomal location. Details including accession numbers for each gene, average number of matching transcripts per time point and chromosome number is gathered in Supplementary File 3 while phylogenies and sequence identities for cathepsins can be found in Supplementary Files 6, 7. NA, not applicable due to lacking data; and ns, not significant*.

In mammals, MHCII transcription is also dependent upon the CREB-RFX-NFY enhanceosome with CIITA as the master regulator (77). Although the single ssCIITA gene has a log_2_ fold increase of 2.3 at 24-hps, the complete lack of RFX5, NFYA and NFYC transcripts also here point to STAT1 and IRF1 as responsible transcription factors for MHCII transcription (Table 1, Supplementary File 3). Surprisingly, in zebrafish, IFNg induced IRF1 and subsequently CIITA, where IRF1 either alone or in combination with CIITA was sufficient to induced MHCII transcription through binding to ISRE elements in the MHCII promoter (78). The enhanceosome was thus not involved in IFNg induction of MHCII expression. Our findings suggest that this may also be the case for MHCII induction in Atlantic salmon and raises the possibility that instead of CIITA, NLRC5 may collaborate with IRF1 to induce transcription of MHCI. If and how STATs also make a contribution remains to be established.

Cathepsin S and L are essential for MHCII maturation through trimming of Ii, but also contribute to the peptide repertoire available for MHCII (79). In Atlantic salmon there are 10 cathepsin L genes (Supplementary Files 5–7). Transcripts are highly abundant from one gene only and none of the gene duplicates are affected by rIFNg (Table 4, Supplementary File 3). For cathepsin S, the two 4R-WGD homeolog Atlantic salmon genes ssCTSS1a and ssCTSS1b are both induced by rIFNg (Supplementary Files 3, 5–7). They reach log_2_ fold values of 1.6 and 3.2 at 24-hps where ssCTSS1a is by far the most transcribed gene. In mammals, IFNg induced cathepsin S transcription is mediated by IRF1 (80), again pointing to a master regulatory role for ssIRF1 in rIFNg mediated induction of Atlantic salmon MHC pathway genes.

### Selective Stimulation of Duplicate Genes

Lien et al. (17) found that overall 55 percent of Atlantic salmon 4-WGD duplicates were retained as two functional gene copies where neo-functionalization was more predominant than sub-functionalization. In our material, 41 of 71 homeolog 4-WGD gene pairs analyzed in detail displayed expression of both duplicates where 13 of these had 4 times or higher expression levels for one of the homeologs (Supplementary File 3). Eight gene pairs were not expressed in our material while 22 gene pairs showed expression of one homeolog only. Perhaps not unexpectedly as our model system was a cell line, only one homeolog being expressed was more common for transcription factors (15/33) than for MHCI pathway genes where both duplicates were mostly expressed (19/31). Transcription factors may be differentially regulated in different cell lines, while there may be a functional advantage of having duplicate expressed MHC pathway genes.

Comparing log2 fold change for all gene duplicates displays a varied pattern (Table 5). For JAK and STAT genes, rIFNg has a major impact on only one of the duplicates. For IRF1 and the many of the first line of defense genes, the alternative approach is operational. Both the IRF1 homeologs are highly induced by rIFNg and peak at 2-hps.

**Table 5:** Log_2_-fold change for selected genes and their gene duplicates.

**Gene**	**Individual gene copies**											
	**1**	**2**	**3**	**4**	**5**	**6**	**7**	**8**	**9**	**10**	**11**	**12**
JAK1	1a_-0.4	1b_0.5^*^	1.La_2.5	1.Lb_nd								
JAK2	1a_nd	1b_1.5	2.2_-0.2									
STAT1	1a_1.3^*^	1b_nd	2a_2.9	2b_nd								
STAT2	a_0.7	b_ns										
IRF1	a_5.4^*^	b_6.8^*^										
NLRC5	a_2.6	b_4.36										
CIITA	2.3											
SOCS1	5.8^*^	5.8^*^	6.3^*^	ns	nd							
MX	2.0	ns	1.4	4.9	6.8	4.3	3.9	7.6	nd	nd		
IFI44	3.0	3.1	ns	nd	ns	1.0						
IFI44.L	1a_16	1b1_7.1^*^	1b2_10.9	1b3_7.3	1b4_4.5	2_nd						
CK10	6.4											
CK13	a_8.6	b_6.1^*^										
CXCL11.L1	7.2^*^	6.8^*^	7.3^*^									
UBA	2.3											
TAP1	2.6											
TAP2	a_3.6	b_5.2	c_2.6									
TAPBP	a_4.0	b_ns	c_ns									
TAPBPL	1a_1.7	1b_1.5	2_3.0									
PSMA6	1a_ns	1b_0.5	2a_2.9	2b_ns	3_0.3							
PSMA7	a_1.5	b_nd										
PSMB8	a_2.7	b_3.0										
PSMB13	a_2.3	b_2.8										
PSMB12	a_1.8	b_1.9										
PSMB9	a_1.8	b_2.5										
PSMB10	a_2.0	b_2.0										
PSME1	a_1.3	b_1.8										
PSME2	a_1.4	b_2.0										
ERAP1	2.4											
ERAP2	a_2.0	b_nd										
b2m	1.7	1.4	2.4	2.1	1.5	0.5	1.6	1.0	0.4	1.2	1.3	0.7
CTSS	a_1.6	b_3.2										

log_2_ fold change at 24-hps compared against time zero for most genes while log_2_ fold change for those that peaked at 2-hps compared against time zero are shown with ^*^. Expression levels are color coded as follows: blue shading is down-regulated; white with black font is not expressed or not significantly upregulated; between 0 and 1 is 12% orange shading with black font; between 1 and 2 is 25% orange shading with black font; between 2 and 4 is 50% orange shading with black font; while log_2_ fold change above 4 is 75% orange shading with white font. Gene duplicate names are either shown inside cells or conform with row numbers show on top of the table. Log_2_ fold change values are given with one decimal only. nd is not detected, ns is not significant. Background data can be found in Supplementary Files 2–4.

Also for the CC and CXC chemokines CK10, CK13, and CXCL11.L genes, all gene duplicates are heavily upregulated where some genes peak at 2-hps and other continue to increase until 24-hps and potentially even longer. This suggest that there is a functional advantage of having several genes responding to stimulation potentially with slightly different responses as seen for CK13a and -b. SOCS1, a negative regulator of the JAK/STAT pathway, has three highly induced genes that peak at 2-hps, thus providing an early regulation of the rIFNg induction.

Also five of the six IRF44.L genes are heavily induced by rIFNg with one peaking at 2-.hps while the remaining displayed increased expression until 24-hps. Only two of the six IFI44 genes have log2 fold values above 2.9. Future studies of IFI44L may explain why these genes are so heavily stimulation.

For the MHC pathway genes, the rIFNg stimulation is less pronounced with log2 fold values below 5.2. All the duplicate MHC linked proteasome genes display similar induction levels, although the ssPSMB8a and ssPSMB9a genes only display low transcription levels. Also the ssPSME1 and 2 genes are expressed in duplicate, but there the expression and induction levels are similar for both genes.

All ssTAP2 genes are expressed, but although the ssTAP2b gene linked to the non-classical genes display the highest log_2_ fold change, the UBA-linked ssTAP2a gene has an 18 times higher transcript level. The UBA-linked ssTAPBPa gene is also unique in responding to rIFNg while the –b duplicate has miniscule transcript levels. The three un-linked ssTABPL genes are all induced by rIFNg, where the ssTAPBPL2 gene is most induced. How they affect MHCI peptide loading remains to be defined.

## Conclusion

Recombinant interferon gamma strongly upregulated a panel of expected early response genes that peaked at 2-hps. The observed transcriptional response could be compatible with several cell types, but suggests that SHK-I cells are not professional antigen-presenting cells, but have a more endothelial-like phenotype. MHC pathway genes had a slower response, with highest induction levels at 24-hps and potentially still rising. Atlantic salmon gene duplicates showed a range of rIFNg response patterns. While rIFNg upregulated all duplicates for some genes such as ssIRF1 and ssIFI44.L, others such as ssTAPBP and ssERAP2 only responded with one or a few of the duplicates.

Lacking expression of both ssRFX5 and ssNFYA/B components, it is unlikely that the enhanceosome was involved in regulation of MHC pathway genes in our material. Instead, ssIRF1 alone or in combination with ssSTAT1 seems to be the master regulator(s) of the IFNg response in Atlantic salmon. The enhanceosome may have a more predominant role in other cell types. If IRF1 also collaborates with CIITA and possibly NLRC5 in regulating IFNg induction of MHCI and MHCII expression in Atlantic salmon as in zebrafish remains to be established. Overall, our results show the importance of deciphering between gene duplicates as they often respond very differently to various stimulations and most likely have very different functions.

## Data Availability Statement

The datasets generated for this study can be found in online repositories. The names of the repository/repositories and accession number(s) can be found at: https://www.ncbi.nlm.nih.gov/, PRJNA637094.

## Author Contributions

UG was responsible for conceptualization, methodology, investigation, data visualization, formal analysis, project administration, funding acquisition, and prepared the original manuscript. AS contributed with software, formal analysis, data curation, and visualization. JF contributed with formal analysis and data visualization. All authors contributed to manuscript review and editing.

## Conflict of Interest

The authors declare that the research was conducted in the absence of any commercial or financial relationships that could be construed as a potential conflict of interest.

## References

[1] Boehm U Klamp T Groot M Howard JC. Cellular responses to interferon-gamma. Annu Rev Immunol. (1997) 15:749–95. 10.1146/annurev.immunol.15.1.749 9143706

[2] Klein J The Natural History of the Major Histocompatibility complex. New York, NY: John Wiley & Sons (1986).

[3] Robinson J Barker DJ Georgiou X Cooper MA Flicek P Marsh SGE. IPD-IMGT/HLA database. Nucleic Acids Res. (2020) 48:D948–55. 10.1093/nar/gkz950 31667505 PMC7145640

[4] Meissner TB Liu YJ Lee KH Li A Biswas A van Eggermond MC. NLRC5 cooperates with the RFX transcription factor complex to induce MHC class I gene expression. J Immunol. (2012) 188:4951–8. 10.4049/jimmunol.1103160 22490869 PMC3345046

[5] Sachini N Papamatheakis J. NF-Y and the immune response: dissecting the complex regulation of MHC genes. Biochim Biophys Acta Gene Regul Mech. (2017) 1860:537–42. 10.1016/j.bbagrm.2016.10.013 27989934

[6] Ludigs K Seguin-Estevez Q Lemeille S Ferrero I Rota G Chelbi S. NLRC5 exclusively transactivates MHC class I and related genes through a distinctive SXY module. PLoS Genet. (2015) 11:e1005088. 10.1371/journal.pgen.1005088 25811463 PMC4374748

[7] Jongsma MLM Guarda G Spaapen RM. The regulatory network behind MHC class I expression. Mol Immunol. (2019) 113:16–21. 10.1016/j.molimm.2017.12.005 29224918

[8] Gobin SJ van Zutphen M Woltman AM van den Elsen PJ. Transactivation of classical and nonclassical HLA class I genes through the IFN-stimulated response element. J Immunol. (1999) 163:1428–34. 10415043

[9] Basler M Kirk CJ Groettrup M. The immunoproteasome in antigen processing and other immunological functions. Curr Opin Immunol. (2013) 25:74–80. 10.1016/j.coi.2012.11.004 23219269

[10] Cascio P. PA28alphabeta: the enigmatic magic ring of the proteasome? Biomolecules. (2014) 4:566–84. 10.3390/biom4020566 24970231 PMC4101498

[11] Neefjes J Jongsma ML Paul P Bakke O. Towards a systems understanding of MHC class I and MHC class II antigen presentation. Nat Rev Immunol. (2011) 11:823–36. 10.1038/nri3084 22076556

[12] Chen H Li L Weimershaus M Evnouchidou I van Endert P Bouvier M. ERAP1-ERAP2 dimers trim MHC I-bound precursor peptides; implications for understanding peptide editing. Sci Rep. (2016) 6:28902. 10.1038/srep28902 27514473 PMC4981824

[13] Boyle LH Hermann C Boname JM Porter KM Patel PA Burr ML. Tapasin-related protein TAPBPR is an additional component of the MHC class I presentation pathway. Proc Natl Acad Sci USA. (2013) 110:3465–70. 10.1073/pnas.1222342110 23401559 PMC3587277

[14] Hermann C van Hateren A Trautwein N Neerincx A Duriez PJ Stevanovic S. TAPBPR alters MHC class I peptide presentation by functioning as a peptide exchange catalyst. Elife. (2015) 4:e09617. 10.7554/eLife.09617 26439010 PMC4718805

[15] Jaillon O Aury JM Brunet F Petit JL Stange-Thomann N Mauceli E. Genome duplication in the teleost fish *Tetraodon nigroviridis* reveals the early vertebrate proto-karyotype. Nature. (2004) 431:946–57. 10.1038/nature03025 15496914

[16] Grimholt U. Whole genome duplications have provided teleosts with many roads to peptide loaded MHC class I molecules. BMC Evol Biol. (2018) 18:25. 10.1186/s12862-018-1138-9 29471808 PMC5824609

[17] Lien S Koop BF Sandve SR Miller JR Kent MP Nome T. The Atlantic salmon genome provides insights into rediploidization. Nature. (2016) 533:200–5. 10.1038/nature17164 27088604 PMC8127823

[18] Chen Z Omori Y Koren S Shirokiya T Kuroda T Miyamoto A. *De novo* assembly of the goldfish (*Carassius auratus*) genome and the evolution of genes after whole-genome duplication. Sci Adv. (2019) 5:eaav0547. 10.1126/sciadv.aav0547 31249862 PMC6594761

[19] Maccari G Robinson J Ballingall K Guethlein LA Grimholt U Kaufman J. IPD-MHC 2.0: an improved inter-species database for the study of the major histocompatibility complex. Nucleic Acids Res. (2017) 45:D860–4. 10.1093/nar/gkw1050 27899604 PMC5210539

[20] Dijkstra JM Yoshiura Y Kiryu I Aoyagi K Kollner B Fischer U. The promoter of the classical MHC class I locus in rainbow trout (*Oncorhynchus mykiss*). Fish Shellfish Immunol. (2003) 14:177–85. 10.1006/fsim.2002.0431 12526881

[21] Jorgensen SM Lyng-Syvertsen B Lukacs M Grimholt U Gjoen T. Expression of MHC class I pathway genes in response to infectious salmon anaemia virus in Atlantic salmon (*Salmo salar* L.) cells. Fish.Shellfish Immunol. (2006) 21:548–60. 10.1016/j.fsi.2006.03.004 16772112

[22] Grimholt U Tsukamoto K Azuma T Leong J Koop BF Dijkstra JM. A comprehensive analysis of teleost MHC class I sequences. BMC Evol Biol. (2015) 15:352. 10.1186/s12862-015-0309-1 25888517 PMC4364491

[23] Sun B Skjaeveland I Svingerud T Zou J Jorgensen J Robertsen B. Antiviral activity of salmonid gamma interferon against infectious pancreatic necrosis virus and salmonid alphavirus and its dependency on type I interferon. J Virol. (2011) 85:9188–98. 10.1128/JVI.00319-11 21697489 PMC3165780

[24] Dannevig BH Falk K Press CM. Propagation of infectious salmon anaemia (ISA) virus in cell culture. Vet Res. (1995) 26:438–42. 8581019

[25] Hetland DL Jorgensen SM Skjodt K Dale OB Falk K Xu C. In situ localisation of major histocompatibility complex class I and class II and CD8 positive cells in infectious salmon anaemia virus (ISAV)-infected Atlantic salmon. Fish Shellfish Immunol. (2010) 28:30–9. 10.1016/j.fsi.2009.09.011 19766193

[26] Zhao H Stet RJ Skjodt K Savelkoul HF. Expression and characterization of recombinant single-chain salmon class I MHC fused with beta2-microglobulin with biological activity. Fish Shellfish Immunol. (2008) 24:459–66. 10.1016/j.fsi.2008.01.003 18280180

[27] Bushnell B BBMap: A Fast, Accurate, Splice-Aware Aligner. Berkeley, CA: Lawrence Berkeley National Lab (2014).

[28] Kim D Langmead B Salzberg SL. HISAT: a fast spliced aligner with low memory requirements. Nat Methods. (2015) 12:357–60. 10.1038/nmeth.3317 25751142 PMC4655817

[29] Liao Y Smyth GK Shi W. featureCounts: an efficient general purpose program for assigning sequence reads to genomic features. Bioinformatics. (2014) 30:923–30. 10.1093/bioinformatics/btt656 24227677

[30] Love MI Huber W Anders S. Moderated estimation of fold change and dispersion for RNA-seq data with DESeq2. Genome Biol. (2014) 15:550. 10.1186/s13059-014-0550-8 25516281 PMC4302049

[31] Huang da W Sherman BT Lempicki RA. Systematic and integrative analysis of large gene lists using DAVID bioinformatics resources. Nat Protoc. (2009) 4:44–57. 10.1038/nprot.2008.211 19131956

[32] Larkin MA Blackshields G Brown NP Chenna R McGettigan PA McWilliam H. Clustal W and Clustal X version 2.0. Bioinformatics. (2007) 23:2947–8. 10.1093/bioinformatics/btm404 17846036

[33] Kumar S Stecher G Tamura K. MEGA7: molecular evolutionary genetics analysis version 7.0 for bigger datasets. Mol Biol Evol. (2016) 33:1870–4. 10.1093/molbev/msw054 27004904 PMC8210823

[34] Dannevig BH Brudeseth BE Gjøen T Rode M Wregeland HI Evensen Ø. Characterisation of a long-term cell line (SHK-1) developed from the head kidney of Atlantic salmon (*Salmo sala*r L.). Fish Shellfish Imunol. (1997) 7:213–26. 7782764

[35] Aamelfot M Weli SC Dale OB Koppang EO Falk K. Characterisation of a monoclonal antibody detecting Atlantic salmon endothelial and red blood cells, and its association with the infectious salmon anaemia virus cell receptor. J Anat. (2013) 222:547–57. 10.1111/joa.12033 23439106 PMC3633344

[36] Sumanas S Jorniak T Lin S. Identification of novel vascular endothelial-specific genes by the microarray analysis of the zebrafish cloche mutants. Blood. (2005) 106:534–41. 10.1182/blood-2004-12-4653 15802528 PMC1895181

[37] Sumanas S Lin S. Ets1-related protein is a key regulator of vasculogenesis in zebrafish. PLoS Biol. (2006) 4:e10. 10.1371/journal.pbio.0040010 16336046 PMC1310653

[38] Ellertsdottir E Berthold PR Bouzaffour M Dufourcq P Trayer V Gauron C. Developmental role of zebrafish protease-activated receptor 1 (PAR1) in the cardio-vascular system. PLoS ONE. (2012) 7:e42131. 10.1371/journal.pone.0042131 22860064 PMC3408399

[39] Aird WC Endothelial cell heterogeneity. Cold Spring Harb Perspect Med. (2012) 2:a006429 10.1101/cshperspect.a006429 22315715 PMC3253027

[40] Indraccolo S Pfeffer U Minuzzo S Esposito G Roni V Mandruzzato S. Identification of genes selectively regulated by IFNs in endothelial cells. J Immunol. (2007) 178:1122–35. 10.4049/jimmunol.178.2.1122 17202376

[41] Dehler CE Lester K Della Pelle G Jouneau L Houel A Collins C. Viral resistance and IFN signaling in STAT2 knockout fish cells. J Immunol. (2019) 203:465–75. 10.4049/jimmunol.1801376 31142600 PMC6612602

[42] Sobhkhez M Skjesol A Thomassen E Tollersrud LG Iliev DB Sun B. Structural and functional characterization of salmon STAT1, STAT2 and IRF9 homologs sheds light on interferon signaling in teleosts. FEBS Open Bio. (2014) 4:858–71. 10.1016/j.fob.2014.09.007 25379383 PMC4215117

[43] Liau NPD Laktyushin A Lucet IS Murphy JM Yao S Whitlock E. The molecular basis of JAK/STAT inhibition by SOCS1. Nat Commun. (2018) 9:1558. 10.1038/s41467-018-04013-1 29674694 PMC5908791

[44] Nie L Xiong R Zhang YS Zhu LY Shao JZ Xiang LX. Conserved inhibitory role of teleost SOCS-1s in IFN signaling pathways. Dev Comp Immunol. (2014) 43:23–9. 10.1016/j.dci.2013.10.007 24183820

[45] Robertsen B Greiner-Tollersrud L Jorgensen LG. Analysis of the Atlantic salmon genome reveals a cluster of Mx genes that respond more strongly to IFN gamma than to type I IFN. Dev Comp Immunol. (2019) 90:80–9. 10.1016/j.dci.2018.09.004 30195710

[46] Wang T Liu F Tian G Secombes CJ Wang T. Lineage/species-specific expansion of the Mx gene family in teleosts: Differential expression and modulation of nine Mx genes in rainbow trout *Oncorhynchus mykiss*. Fish Shellfish Immunol. (2019) 90:413–30. 10.1016/j.fsi.2019.04.303 31063803

[47] Laing KJ Secombes CJ. Trout CC chemokines: comparison of their sequences and expression patterns. Mol Immunol. (2004) 41:793–808. 10.1016/j.molimm.2004.03.038 15234559

[48] Chen F Lu XJ Nie L Ning YJ Chen J. Molecular characterization of a CC motif chemokine 19-like gene in ayu (*Plecoglossus altivelis*) and its role in leukocyte trafficking. Fish Shellfish Immunol. (2018) 72:301–8. 10.1016/j.fsi.2017.11.012 29128493

[49] Lacy P. Editorial: secretion of cytokines and chemokines by innate immune cells. Front Immunol. (2015) 6:190. 10.3389/fimmu.2015.00190 25954279 PMC4406090

[50] Zou J Carrington A Collet B Dijkstra JM Yoshiura Y Bols N. Identification and bioactivities of IFN-gamma in rainbow trout *Oncorhynchus mykiss*: the first Th1-type cytokine characterized functionally in fish. J Immunol. (2005) 175:2484–94. 10.4049/jimmunol.175.4.2484 16081820

[51] Castro R Martin SA Bird S Lamas J Secombes CJ. Characterisation of gamma-interferon responsive promoters in fish. Mol Immunol. (2008) 45:3454–62. 10.1016/j.molimm.2008.03.015 18457879

[52] Chen J Xu Q Wang T Collet B Corripio-Miyar Y Bird S. Phylogenetic analysis of vertebrate CXC chemokines reveals novel lineage specific groups in teleost fish. Dev Comp Immunol. (2013) 41:137–52. 10.1016/j.dci.2013.05.006 23701879

[53] Torraca V Cui C Boland R Bebelman JP van der Sar AM Smit MJ. The CXCR3-CXCL11 signaling axis mediates macrophage recruitment and dissemination of mycobacterial infection. Dis Model Mech. (2015) 8:253–69. 10.1242/dmm.017756 25573892 PMC4348563

[54] Kitamura A Takahashi K Okajima A Kitamura N. Induction of the human gene for p44, a hepatitis-C-associated microtubular aggregate protein, by interferon-alpha/beta. Eur J Biochem. (1994) 224:877–83. 10.1111/j.1432-1033.1994.00877.x 7925411

[55] Power D Santoso N Dieringer M Yu J Huang H Simpson S. IFI44 suppresses HIV-1 LTR promoter activity and facilitates its latency. Virology. (2015) 481:142–50. 10.1016/j.virol.2015.02.046 25776761 PMC4437885

[56] DeDiego ML Nogales A Martinez-Sobrido L Topham DJ Interferon-induced protein 44 interacts with cellular FK506-binding protein 5, negatively regulates host antiviral responses, and supports virus replication. mBio. (2019) 10:e01839-19. 10.1128/mBio.01839-19 31455651 PMC6712396

[57] Briolat V Jouneau L Carvalho R Palha N Langevin C Herbomel P. Contrasted innate responses to two viruses in zebrafish: insights into the ancestral repertoire of vertebrate IFN-stimulated genes. J Immunol. (2014) 192:4328–41. 10.4049/jimmunol.1302611 24683187

[58] Meissner TB Li A Biswas A Lee KH Liu YJ Bayir E. NLR family member NLRC5 is a transcriptional regulator of MHC class I genes. Proc Natl Acad Sci USA. (2010) 107:13794–9. 10.1073/pnas.1008684107 20639463 PMC2922274

[59] van den Elsen PJ. Expression regulation of major histocompatibility complex class I and class II encoding genes. Front Immunol. (2011) 2:48. 10.3389/fimmu.2011.00048 22566838 PMC3342053

[60] Rene C Lozano C Eliaou JF. Expression of classical HLA class I molecules: regulation and clinical impacts: julia bodmer award review 2015. HLA. (2016) 87:338–49. 10.1111/tan.12787 27060357

[61] Min W Pober JS Johnson DR. Kinetically coordinated induction of TAP1 and HLA class I by IFN-gamma: the rapid induction of TAP1 by IFN-gamma is mediated by Stat1 alpha. J Immunol. (1996) 156:3174–83. 8617938

[62] Santambrogio L Berendam SJ Engelhard VH. The antigen processing and presentation machinery in lymphatic endothelial cells. Front Immunol. (2019) 10:1033. 10.3389/fimmu.2019.01033 31134089 PMC6513971

[63] McConnell SC Hernandez KM Wcisel DJ Kettleborough RN Stemple DL Yoder JA. Alternative haplotypes of antigen processing genes in zebrafish diverged early in vertebrate evolution. Proc Natl Acad Sci USA. (2016) 113:E5014–23. 10.1073/pnas.1607602113 27493218 PMC5003237

[64] van Hateren A Carter R Bailey A Kontouli N Williams AP Kaufman J. A mechanistic basis for the co-evolution of chicken tapasin and major histocompatibility complex class I (MHC I) proteins. J Biol Chem. (2013) 288:32797–808. 10.1074/jbc.M113.474031 24078633 PMC3820913

[65] Guillaume B Chapiro J Stroobant V Colau D Van Holle B Parvizi G. Two abundant proteasome subtypes that uniquely process some antigens presented by HLA class I molecules. Proc Natl Acad Sci USA. (2010) 107:18599–604. 10.1073/pnas.1009778107 20937868 PMC2972972

[66] McConnell SC Restaino AC de Jong JL. Multiple divergent haplotypes express completely distinct sets of class I MHC genes in zebrafish. Immunogenetics. (2014) 66:199–213. 10.1007/s00251-013-0749-y 24291825 PMC3965299

[67] Murata S Takahama Y Kasahara M Tanaka K. The immunoproteasome and thymoproteasome: functions, evolution and human disease. Nat Immunol. (2018) 19:923–31. 10.1038/s41590-018-0186-z 30104634

[68] Martin SA Mohanty BP Cash P Houlihan DF Secombes CJ. Proteome analysis of the Atlantic salmon (Salmo salar) cell line SHK-1 following recombinant IFN-gamma stimulation. Proteomics. (2007) 7:2275–86. 10.1002/pmic.200700020 17549796

[69] Kohda K Ishibashi T Shimbara N Tanaka K Matsuda Y Kasahara M. Characterization of the mouse PA28 activator complex gene family: complete organizations of the three member genes and a physical map of the approximately 150-kb region containing the alpha- and beta-subunit genes. J Immunol. (1998) 160:4923–35. 9590240

[70] Ma W Lehner PJ Cresswell P Pober JS Johnson DR. Interferon-gamma rapidly increases peptide transporter (TAP) subunit expression and peptide transport capacity in endothelial cells. J Biol Chem. (1997) 272:16585–90. 10.1074/jbc.272.26.16585 9195970

[71] Walker BA Hunt LG Sowa AK Skjodt K Gobel TW Lehner PJ. The dominantly expressed class I molecule of the chicken MHC is explained by coevolution with the polymorphic peptide transporter (TAP) genes. Proc Natl Acad Sci USA. (2011) 108:8396–401. 10.1073/pnas.1019496108 21536896 PMC3100931

[72] Chang SC Momburg F Bhutani N Goldberg AL. The ER aminopeptidase, ERAP1, trims precursors to lengths of MHC class I peptides by a “molecular ruler” mechanism. Proc Natl Acad Sci USA. (2005) 102:17107–12. 10.1073/pnas.0500721102 16286653 PMC1287962

[73] Evnouchidou I Weimershaus M Saveanu L van Endert P. ERAP1-ERAP2 dimerization increases peptide-trimming efficiency. J Immunol. (2014) 193:901–8. 10.4049/jimmunol.1302855 24928998

[74] Collins T Korman AJ Wake CT Boss JM Kappes DJ Fiers W. Immune interferon activates multiple class II major histocompatibility complex genes and the associated invariant chain gene in human endothelial cells and dermal fibroblasts. Proc Natl Acad Sci USA. (1984) 81:4917–21. 10.1073/pnas.81.15.4917 6431411 PMC391603

[75] Pober JS Merola J Liu R Manes TD. Antigen presentation by vascular cells. Front Immunol. (2017) 8:1907. 10.3389/fimmu.2017.01907 29312357 PMC5744398

[76] Grimholt U. MHC and Evolution in Teleosts. Biology. (2016) 5:6. 10.3390/biology5010006 26797646 PMC4810163

[77] Masternak K Muhlethaler-Mottet A Villard J Zufferey M Steimle V Reith W. CIITA is a transcriptional coactivator that is recruited to MHC class II promoters by multiple synergistic interactions with an enhanceosome complex. Genes Dev. (2000) 14:1156–66. 10.1101/gad.14.9.1156 10809673 PMC316580

[78] Hou J Chen SN Gan Z Li N Huang L Huo HJ. In primitive zebrafish, mhc class II expression is regulated by IFN-gamma, IRF1, and two forms of CIITA. J Immunol. (2020) 204:2401–15. 10.4049/jimmunol.1801480 32188757

[79] Hsieh CS deRoos P Honey K Beers C Rudensky AY. A role for cathepsin L and cathepsin S in peptide generation for MHC class II presentation. J Immunol. (2002) 168:2618–25. 10.4049/jimmunol.168.6.2618 11884425

[80] Storm van's Gravesande K Layne MD Ye Q Le L Baron RM Perrella MA. IFN regulatory factor-1 regulates IFN-gamma-dependent cathepsin S expression. J Immunol. (2002) 168:4488–94. 10.4049/jimmunol.168.9.4488 11970993

