# Supplementary material for: Selective Stimulation of Duplicated Atlantic Salmon MHC Pathway Genes by Interferon-Gamma
Source: Front Immunol. 2020 Oct 6;11:571650. doi: 10.3389/fimmu.2020.571650 (PMC7573153; doi:10.3389/fimmu.2020.571650)
Supplement: Supplementary file 1 [file Data_Sheet_1.pdf]

Supplementary File 1 (SF1). Western blots of SHK1 lysates

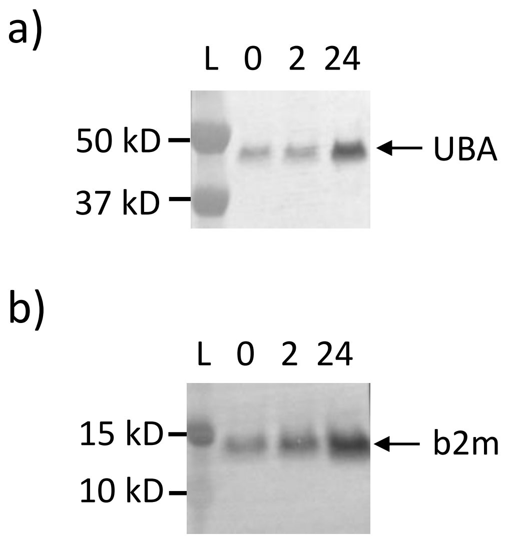


Western blots of SHK1 lysates of unstimulated cells (Lane 0; 0-hps), rIFNg stimulated for 2 hours (Lane 2; 2-hps) or stimulated for 24 hours (Lane 24; 24-hps) using UBA (a) and b2m (b) spesific antibodies [main text references Zhao et al.2008 and Hetland et al.2010)] . Kilodalton (kD) protein ladder bands (L) are shown on the left hand side of each figure. One of four repeat experiments is shown. Negative controls are not shown.
