# Supplementary material for: Selective Stimulation of Duplicated Atlantic Salmon MHC Pathway Genes by Interferon-Gamma
Source: Front Immunol. 2020 Oct 6;11:571650. doi: 10.3389/fimmu.2020.571650 (PMC7573153; doi:10.3389/fimmu.2020.571650)
Supplement: Supplementary file 4 [file Data_Sheet_4.pdf]

Supplementary file 4 (SF4). Functional annotation of DEGs

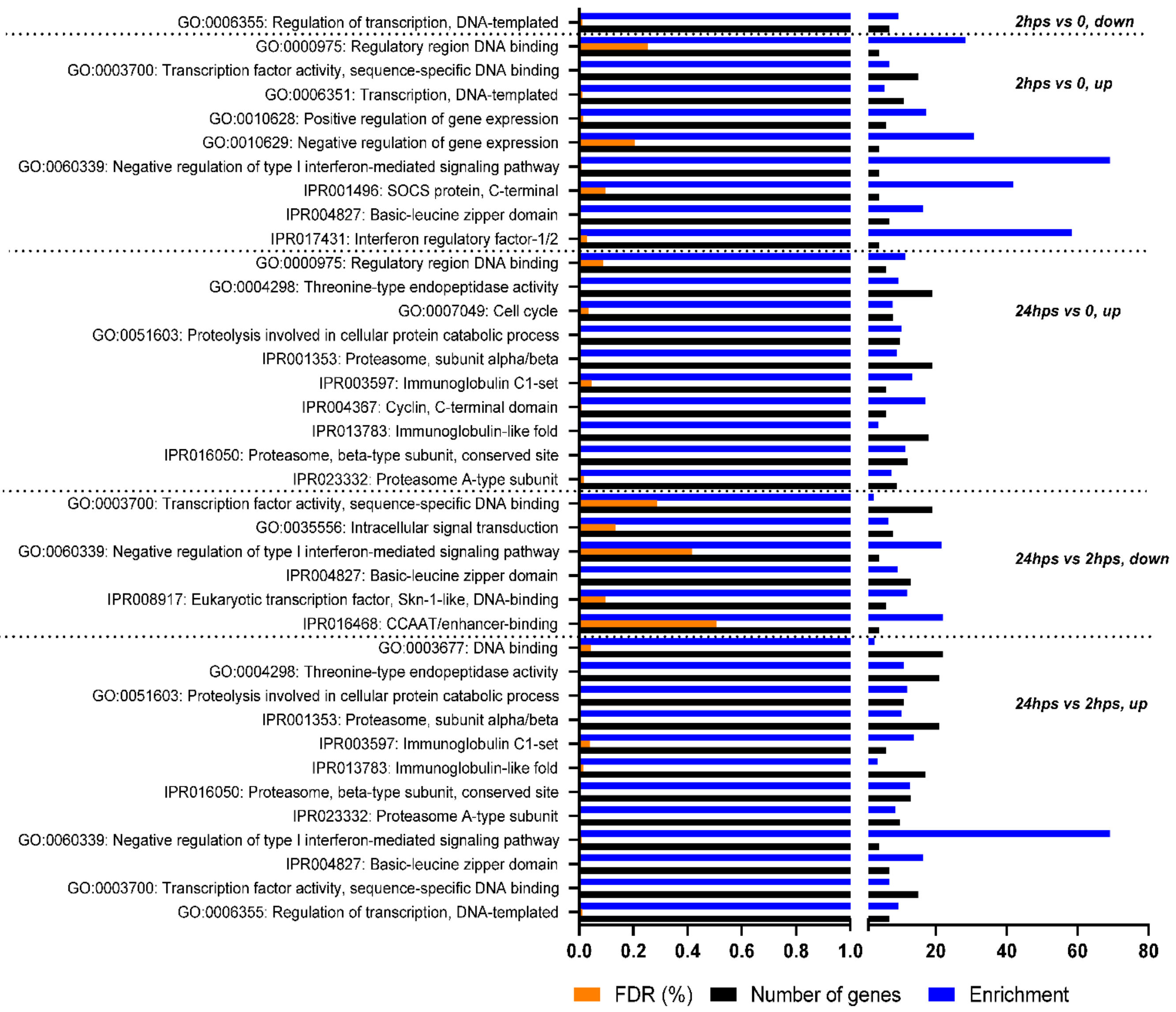

Supplementary file 4: Functional annotation of DEGs. A gene ontology analysis of DEGs regulated 2-fold or more was performed using DAVID, as described in the materials and methods section. Blue bars show enrichment, orange bars show % false discovery rate (FDR) and black bars show the number of genes that match each GO term. The figure shows enrichment identified in lists of DEGs from all contrasts, given in italics to the right. No enrichment was identified in the contrast 24h vs 0, down. The number of genes included in each list (recognized by/submitted to DAVID) were: 2hps vs 0 down (197/200), 2hps vs 0 up (897/939), 24hps vs 0 down (2418/2456), 24hps vs 0 up (2326/2398), 24hps vs 2hps down (2357/2392), and 24hps vs 2hps up (1708/1703). Only terms based on 3 genes or more and with a threshold of FDR<1/ bonferroni-adjusted p<0,05 are shown. The following GO terms were omitted due to redundancy: IPR023333: Proteasome B-type subunit, IPR000243: Peptidase T1A, proteasome beta-subunit, IPR000426: Proteasome, alpha-subunit, N-terminal domain, and IPR007110:Immunoglobulin-like domain.
