# Supplementary material for: Selective Stimulation of Duplicated Atlantic Salmon MHC Pathway Genes by Interferon-Gamma
Source: Front Immunol. 2020 Oct 6;11:571650. doi: 10.3389/fimmu.2020.571650 (PMC7573153; doi:10.3389/fimmu.2020.571650)
Supplement: Supplementary file 5 [file Data_Sheet_5.pdf]

Supplementary File 5 (SF5). Deduced amino acid sequences of selected genes

SF5	Gene	Page
SF5.1	JAK1 and JAK2 sequences	1
SF5.2	STAT1 and STAT2 sequences	4
SF5.3	Other transcription factors (NF-Ys, RFXs, NLRC5, NF-kB, CIITA)	7
SF5.4	IRF1 and IRF9	11
SF5.5	MX sequences	11
SF5.6	CC and CXC chemokines	13
SF5.7	SOCS1 sequences	13
SF5.8	IFI44 sequences	14
SF5.9	Beta2-microglobulin (b2m) sequences	15
SF5.10	Peptide loading complex genes (TAP, TAPBP, CALR, CANX, ERP57, PDIA, ERAP)	19
SF5.11	MHC class I sequences	23
SF5.12	Proteasome subunits (PSMA, PSMB, PSME) sequences	26
SF5.13	MHC class II pathway sequences	32

SF5.1 JAK1 and JAK2 tyrosine-protein kinase sequences

```
>JAK1a XM_014123419.1 XP_013978894.1 Ssa10 NC_027309.1:41.900.001-41.955.907 tyrosine-
protein kinase JAK1 [Salmo salar]
MPSLGVMELGRQLCGKMRRPRRAEGPVTVPMRGLEIHFYLPDIHPLLFLKDSFTAEELCV
EAAKRCYISPLCCNLFGLYDENQQLWYAPNHVIKVTDETCIKLHYRMRFYFTNWHGTSER
ESPVWRHCISKLRGGLGPQRAPEGIPLLDAASLDYLFAQGQNDFQKGLAVVRCPQSEAEQ
HEIENECLGMAVLAITHYAMTREIPLSTASNEISYKRFIPEKLNRQIKQRNILTRLRINN
VFKNFLNEFNSRTVQDSNITLYDLKIKYLSTLESLTRGLGREMLEPRALVVTQEVETNGG
LSDGPESSLAMEVQVTGTTGISYRRKPPNNTLILKEKNKSKKNKFEGKQTKPKKKDTSDG
WVTFCDFHEITHIVIKESTVTIFRQDNKKMEVQLEFKGEALAFAALVDGYFRLTVDAHHY
LCTEVAPSSVVQNLQNCCHGPICTEYANQKLRQEGNEEGTYVLRWSCTDYHYIIMTVVCS
ELDLCESRQVRQYKNFQIEVGSEGFRLYGTETYRSSLRELLDHLSGQNLRTDNLRFQLSR
CCPPQPREVSNLLVVTKDRVLVPQTTLQESQLSFHRILKEEIMQGEHLGLGTRTNIYAGV
LKVKSEEDAGYYPSQELKVVLKVLGSGHRDISLAFFETASMMRQVSHKHIALLYGVCVRH
LENIMVEEFVQLGPLDVFMRRQRSPLSTPWKFQVAKQLASALSYLEDKKLVHGFVCTKNI
LLARDGVDSEGGPFIKLSDPGIPITVLTREECVDRIPWIAPECVKDSANLSVAADKWGFG
TTLWEICYDGEVPLKDKKLTEKERFYAAECQLATPDCKELAELMTHCMTFDPRKRPFFRA
IVRDIDTLEEQNPSIKPVPTLEVDPTVFEKRFLKKIRDLGEGHFGKVELCRYDPRGDRTG
ELVAVKSLKPENREEQSSNLWCEIGILKELYHHNIVKYKGICQEEGGRAIKLIMEYLPAG
SLKEYLPRNKSQTSLKTLLSYAVQICQGMDYLGSRNYIHRDLAARNVLVENESTVKIGDF
GLTKSIKDNEGYYTVKDDLDSPVFWYAPECLVHCKFYLASDVWSFGVTMYELLTYCDSAI
SPMTVFLKMIGPTQGQMTVTRLVKALEEGKRLPRPESCPGTVYELMRKCWESTPDRRITF
KSLIDEFTRMLANNSL
>JAK1b XM_014168828.1 XP_014024302.1 Ssa23 NC_027322.1:9.336.812-9.396.447
MQRKNYLTSLQRRVWMPSPGVMELGRQLCGKMRSRRAEGPVSVPMRGLEIHFYLPNMPQL
GFLKDSFTAEELCVEAAKKCYISPLCHNLFGLYDESRTLWYAPNHLFKITDETCIKLHYR
MRFYFTNWHGTSESESPVWRHCISKLRGGLSPQKVPEGTPLLDSASLDYLFFQGQNDFQK
GLAVVRSQQSEAEQHEIENECLGMAVLAITHYAMTKEIPLSTTSNEISYKRFIPESLNRS
IKQRNILTRLRINNVFKNFLNEFNSRTMLESNITLYDLKIKYLSTLEGLTRGLGREVLEP
RALVLTQEGETNGGLSQGPEPFLAVEVQVTGTTGISYRRKPPNNTLMLKEKTKSKKNKHE
GKQKKDKKNDASDDWVTFCDFHEITHIVIKESSVTIFRQDNKKMEVKLEFRGEALAFAAL
VDGYFRLTVDAHHYLCTEVAPSSVVQNLQNCCHGPICTEYAIQKLRQEGNEEGTYVLRWS
CTDYHNIIMTVVCKEFDLCESRQVHLYKNFQIEVGSEGFRLYGTETYRSSLRELLDHLAR
QNLRTDNLHFQLKRCCPPQPREVSNLLVVTKERVLVPQTPLQESQLSFHRILKEEIVQGE
HLGLGTRTNIYAGVLKVKSAEDEDAGYSSSQDVKVVLKVLGSGHRDISLAFFETASMMRQ
VSHKHIALLYGVCVRHLENIMVEEFVQFGPLDLLMRRQHSPLGTPWKFQVAKQLASALSY
LEDKKLVHGFVCTKNILLARDGVDSEGGPFIKLSDPGIPITVLTREECVDRIPWIAPECL
KDSTNLSVAADKWGFGTTLWEICYDGEVPLKDKKLTEKERFYAAECQLATPDCKELAELM
THCMTYDPHKRPFFRAIVRDIDTLEEQNPSIKPAPTQEVDPTLFQKRFLKRIRDLGEGHF
GKVELCRYDPRGDRTGELVAVKSLKPENREEQSNNLWSEIGILKELYHENIVKYKGICQE
EGGRAIKLIMEYLPAGSLKEYLPRNRGQTSLKTLLSYAVQICQGMDYLGSRNYIHRDLAA
RNVLVENESTVKIGDFGLTKSIKGNEGYYTVKDDLDSPVYWYAPECLVHCKFYLASDVWS
FGVTMYELLTYCENAISPMAMFLKIIGPTQGQMTVTRLVKVLEEGKRLPRPDSCPGTVYE
LMRKCWENTPARRITFKSLIDEFTRMLTNSSL
>JAK1.La XM_014139300.1 XP_013994775.1 ssa14 NC_027313.1:8.762.241-8.786.215
MPRNKAMDMGRQLLVKMQRTRKGKFTPIPVLPQGLEVHFYLRDSHQLEFLRGCYTAEELC
TEAAKKCGISPLCFNLFALYDESMNIWYPPNHTFKIEESTCLKLHYRMRFYFTNWHGAND
NVPRVCRHALKRKNGNGPKTESGGTALLEAASLKYLFAQGQHDFLKGRAAVRNSQNEEEA
HCIENECLGVAVLSITHNALEKNISNPYLGGQISYKKYIPESVNQIIKQRNFLTRVRISR
VFQHFLNEFNNKTVKNNKVNTHDIKVKYLATLETLTRGFGCEMYKPEVLRVTDSEGEIEG
TPTSCNQGQPTQYQVLVSGNTGIKWRRKQQNNAWTAKEKKKSKKYKTDINWKNKPAQDLS
NDWKTFSDFYEITHINIKGSTVTVHKQDNKKMELSLGFHAEALSFATLIDGYFRLTVDAH
HFLCTDVAPPSVVQNLQEGCHGPISMDYTSHKLRQEGNEEGMYVLRWSCIDYDHIILTVK
CNEVDLTDSHPYRSFKIEVGPEGYGLSGTALRQPSLRELMEQLRGQRLSTDGVLFQLRKA
CPPQPREMSNLLLVTKRNAELTYPVQNHVIFHKILNEDIVQEEHLGCGTRTNIYAGKFKI
KSEEEKDVWGSQTYHEVKVVLKVLGSQHRDISMTFFETVSMMRQVSHQHITLLHGVCVRS
HDNIIVEEHVTLGPLDVFMRGHRLELSTSWKYQVAKQLTGALSYLEDKKLVHGYVCAKNV
LVERDGLEGETGPFIKLSSPGVSISALNIQECVERIPWIAPECVRNSQALSVAVDKWGFG
TTLWEICYEGEAPLKDKKLIEKEMFYSAQCSLVTPDCPQLAELITKCMNYDPKRRPFFRA
IVRDLTGVAEQNPVLPPGRVPIQEVDPTVFDTRFLRKIDDLGEGHFGKVELCLYDPRGDG
RGELVAVKSLKPNSKGQLACHLRKEIDTMRELYHHNIVKYKGVCSEEGGRTTKLIMEYLP
AGSLKDYLPKRKHQTDLKRLLHYALQICQGMDYLGSQRFIHRDLAARNVLVENESTVKIG
```

DFGLTKSMKEDKSYYTVKEETDSPVFWYAPECLMDCKFYPASDVWSFGVTLYELMTYCET
SSSPTTVFSEMLHPTKGLMTLTRLVEVLIAGRRLPCPPRCPDTVYSLMRRCWEYDPANRI
QFKGLITELETLLDERHRGDRLAV
>JAK1.Lb XM_014189482.1 XP_014044959.1 ssa03 NC_027302.1:9.567.654-9.594.450
MEVGRQLLVKMRMKRKGEFTSVPVIVQGLKVHFYLEDSPQLEFLHGCYTAEELCTNAAKK
CGISPLCCNLFALYDEVRKIWFPPNHTFKESSHLKLHYRMRFYFTNWHGVNESVPRVCRH
ALKRKNANGPKTEPEGNPLLDTASLKYLFAQGQHDFVKGWATLRNPQNEEEVHYIENECL
GMAVLSISHHALDNNIVIPGVAGQISYKKYIPDRVNQIIKQRNFLTRLRISRVFQDFLND
FNNKTVQSDNVSTHDIKVKYLATLETLTCGFGCEEYEPKVLRVTDSEGEIQGTPTSCNQG
QPTQYQVLVSGNTGIKWRRKQQNNAWTAKEKNKSPKHKTNINWTNKPPQGVSNDWKTFSD
FHEITHINIKGSTVTVHKQDNKKMELSLGFHAEALSFAALIDGYFRLTVDAHHFLCTDVA
PPSVVRNLQEGCHGPIGMDYTSHKLRQEGEEEGMYVLRWSCINYDHILLTVTGNEVDLTN
SRLYRSFKIEVGPEGYSLNGTDLRQPSLRELMEQLRGQTLSTDRVTFQLRKACPPQPREI
SNLLFVTKREAEPTHPIQSQLIFHRILKEDILQEEHLGCGTRTNIYAGKLKIKCEEEKDV
WGSQTHHQVKVVLKVLWSQHRDISMAFFETVSMIHQLSHQHIALLHGVCVRNQDNIIVEE
HVKLGPLDVFMKGCRLQLSTSWKFQVAKQLASVLSYLEDKKLVHGYVSAKNILVERDGLE
GETGPFIKLSSPGVPISALNRQECVERIPWIAPECVRDNQVLSVAVDKWGFGTTLWEICY
DGEAPLKDKKLIEKEMFYSAQCSLVTPDCPQLGELITKCMTYDPKRRPFFRAIVRDLTGV
AEQNPALPPGRVPIQEVDPTVFETRFLRKIKDLGEGHFGKVELCQYDPRGDGRGQLVAVK
SLKPESRGQLWREIDTMRELYHHNIVKYRGVCSEEGGRTTKLIMEYLPAGSLKDYLPWRK
HQTDLKRLLHYALQICQGMDYLGSQRFIHRDLAARNVLVENESTVKIGDFGLTKSMKEDK
SYYTVKEETDSPVFWYAPECLVDCKFYPASDVWSFGVTLYELLTYCETSSSPTTVFLEML
RLTQGQMIITRLVEVLMAGRRLPCPPHCPDAVYSLMRRCWESSPENRIQFKDLITELKLL
LDERHGGDGRAV
>JAK2.1a XM_014125501.1 XP_013980976.1 ssa01 NC_027300.1:1110.012.942.-110.189.997 [Salmo salar]
MVLTSATVITVIRKSYFPVATTFRTDNVQVDVSAEPHMDMECPVCPSSNQNGCVLDPLHV
SEPKQVVAPPCLRVHLYHLGRGRANSPDLTYPSGNYVAEELCIDAAMACGLSPMYCSLFG
LMRESDRMWFPPNHILKLDESANEMLLFRVRYYFPGWYNSVSCAHRYGVNKGLESPVLDD
TVMSYLFAQWRSDFVDGWVSIPVNHEAQEECLGMAVLDMMRMAKESSQAPVDIFNDTSYK
SFLPKDIRARIQEYHFVTRKRIRHRFKKFIQQFCQCNATARDLKLKYLANLEMLLPALYS
ECFRVTEPSAGEVTIVVTGNNGIQWSQGKDIEIEKGFQTYCDFPEVIDISIKQANKGGAI
ESRIVTINRQDHQTLELEFHSLSEALSFVSLVDGYYRLIADAHHYLCKETAPPKLLEAIQ
SYCHGPVSMEFAISKLRRSGNHKGLYILRSSPKDYNKYFLTFVVGSDSQVEYKHGQIVKT
DQGEYILSGAKKSFSSLRELLHCYQKEALRSDGHIFKFTKCCPPKAKDKSNLLVCRGNQG
AEVPPSPSLHRHISQMMFHKIRKEDLVIKDSLGQGTFTKIFCGVRKEVGDYGEIHQMDVI
VKILDKAHRNYSESFFEAASMMSQLSHKHMLLNYGVCVCGEENMMVQEYVKFGSLDTYLK
KNKSSVNITWKLEVAKQLAWAMHYLEDKNLIHGNVCAKNVLLIREEDKKTGNPPFIKLSD
PGISITVLPKDVLVERIPWVPPECIEVPRHLTLATDKWAFGTTLWEICSGGDKPLSTLDC
SKKRLFYEDRHQLPAPKWTELANLINSCMDYEPSNRPSFRAVIRDLNSLFTPDYELLVES
DMVPNRTRGFGFPGTFENQEPALFEERHLIFLKQLGKGNFGSVEMCRYDPLLDNTGEVVA
VKKLQHSTAEHLRDFEREIEILKSLQHDNIVKYKGVCYSAGRRNLHLIMEYLPHGSLRDY
LVKNKDRFDYKKLLLYASQICKGMDYLATKRYIHRDLATRNILVESELRVKIGDFGLTKV
LPQDKDYYTVREPGESPIFWYAPESLTESKFSVGSDVWSFGVVLYELFTHSDKNCSPPAV
FMDKMGRDKQGQMIVYHLIDLLKRGYRLPAPQGCPEEIHNMMTECWNGDPGLRPTFKKLA
QSVDTFRDTVKRAEEPFLEGSAEGDLA
>JAK2.1b XM_014138772.1 XP_013994247.1 Ssa13 NC_027312.1:96.918.887-97.976.821
MDMESALCPSSNQNGCILNPLPVSETKQEQEVAPPPCLRVHLYHLGQGGANSQDLTYPAG
DYVAEELCIDAAKACGLSPLYCSLFGLMRESDRMWFPPNHILKLHQSANYSLLFRVRYYF
PGWYGGSCSFRYGVNKGMDSPVFDDTVMSYLFAQWRSDFVHSWVIIPVNHEAQEECLGMA
VLDMMRMAKESSQSPVDIYNDTSYKSFLPKNMRAHIQEYHFVTRKRIRHRFRKFIQQFSQ
CKATARDLKLKYLVNLETLHPAFYSECFRVTESLEGEVTIVVTGNNGIQWSREEDKEADK
GFQTYCDFPEVIDISIKQANKDGSIESRIVTINRQDSQTLELEFRSLSEALSFVSLVDGY
YRLIADAHHFLCKEVAPPRLLEAIQSYCHGPVSMDFAISKLRRSGNHKGLYILRSSPKDY
NKYFLSFVVGCDSLVEYKHCQIVKTDQGDYILSGAKKSFGSLRELLHRYQKEALRSDGHV
FQFTKCCPPKAKDKSNLLVCRSNQGAEVPLSPSLHRHNISQMVFHKIRKEDLVMKDSLGQ
GTFTKIFCGVRKELGDYGEIHQMDVLVKILDKAHRKYSESFFEAASMMSQLSHKHLLLTY
GVCVCGEENMMVQEYVKFGSLDTYLKKNKSSVNITWKLEVAKQLAWAMHYLEDKNLVHGN
VCAKNVLLIREEDRKTGNPPFIKLSDPGISITVLPKDVLVERIPWVPPECFEEDRHLTLA
TDKWAFGTTLWEICSGGDRPLGTLDCSKKQLFYEDHHQLPAPKWTELANLINSCMDYEPS
NRPSFRAVIRDLNSLFTPEGVDCVLSPAPDYELLVESDMVPNRARGFGFPGAFENQNPAQ
FEERHLIFLKQLGKGNFGSVEMCRYDPLQDNTGEVVAVKKLQHSTAEHLRDFEREIEILK
SLQHDNIVKYKGVCYSAGRRNLRLIMEYLPYGSLRDYLIKNKDRFDYKKLLHYASQICKG
MDYLATKRYIHRDLATRNILVESELRVKIGDFGLTKVLPQDKDYYTVREPGESPIFWYAP

```
ESLTESRFSVGSDVWSFGVVLYELFSDKNCSPPAAFMDKMGSDKQGQMIVYHLIDLLKRG
YRLPAPKGCPEEIQHMMTECWNIDPALRPTFKKLAQTVDMFRDSKEG
>JAK2.2 XM_014171549.1 XP_014027024.1 ssa24 Sequence ID: NC_027323.1:26.874.135-26.911.606
MAGVSVLDMEMPSCPPVPVHQNGTAHREPRGSRPAAAPVLRVHLYHSSLAGVDSTPLSYP
PGDYVVEQLCVNAAKECSVSPLYCSLFGLFRERDGMWFSPNHVFQLDEYANEDMVFRIRY
YFPGWYSSGATRAYRYGVTKGSESPVLDDFVMAYLFAQWRSDFVNGWVKIANTHETQEEC
LGMAVLDMMRIAKERQMSPLDIYNSISYKSFLPKDMRAHIQDCNFVTRKRIRYRFKKFIQ
QFSQCRATARDLKLKYLINMESLEQAFYTERFQVRESSAGQVTIIVTADHGIQWCRDGHK
DSEQDLQTYCDFPDVTDISIKQANKEGSMESRVVTINKQDGKTLELEFNCLSEALSFISL
IDGYYRLTTDAHHYLCKEVAPPALVEAITSHCHGPISMDFAINRLQKSGNKRGLYILRCS
PKDFNKYFLTFTVGVYDGIEYKHCQITKAANGEFNLSGTKRNFSNLQELLSCYQKETVRS
DGIIFQFSKCCPPKSKDKSSLLVSRSNKGSDVPLSPSLQRRNINQMVFHKIRKEDLEYTE
SQGQGTFTKIFKGVRKELGDYGEMHQTDVIMKVLDKAHRNYSESFFEAASMMSQLSHKHL
VLNYGVCVCGEENIMVQEYVKFGSLDTYLKKNKNSVNILWKLEVAKQLAWAMHFLEEKNL
AHGNVCAKNVLLIREEDRKTGNPPFIKLSDPGVSITVLPKDILVERIPWVPPECIEDSTN
LSLAADKWSFGTTLWEICSGGEKPLVTLDNSKKNLFYEDRHQLPAPKWTELANLITSCMD
YEPTFRPSFRVIIRDLNSLFTPEYELIVESDLLPNRTSASPWVTGAFENQEPVQFEERHL
IFLQQLGKGNFGSVEMCRYDPLQDNTGEVVAVKKLQHSTEEHMRDFEREIEILKSLQHEN
IVKYKGVCYSAGRRNLRLIMEYLPYGSLRDYLIKNKPRIDHMKLVHYTAQICKGMEYLAT
KRYIHRDLATRNILVESELRVKIGDFGLSKVLPQDKEYYMVKEPGESPIFWYAPESLTES
KFSVASDIWSFGVVLYELFTHSDKNCSPPAMFMGMMGNDKKGQLIVYHLIELLKSGSRLP
QPVGCPMEMHEIMQECWDKDPCLRPSFKELALRVDLFRDSKET
```

SF5.2 STAT1 and STAT2 sequences

```
>STAT1.1 XM_014149897.1 XP_014005371.1 LG16 NC_027315.1:64.447.884-64.461.742
MAQWCQLQLLESKYLEQVDQLYDDSFPMDIRQYLSKWIESIDWENVAVQDSLATVRFHDL
LAQLDDQHSRFALENNFLLQHNIRKIKRNLQDHFQEDPVHMAMIISRNLKEEQKILAAAK
SIETDRENTQTSMVLEKQKLDNKVKDMKNKVQEADQNVKSLEYLQDEHDFKENTLKNREH
EMNGLTQKQLEHEKLQIAEMCFKLKFKRGEVVGQLAEVLNIAEAVQSDLISEELAEWKKR
QQISCIGGPPNACLDQLQNWFTAVAESLQQVRQQLKELQELEQKYTYDNDPIKQQKGFLE
GRALSLFRNLLEHSLVVERQPCMPTHPQRPLVLKTQVQFTVKLRFLVKLQEFNYQLKVKA
LFDKDVTEKKGFRKFNILGTNTKVMNMEESNGSLAAEFRHLQLKEQKVAGNRTNEGPLIV
TEELHCICFESELNQSGLELKLETISLPIVVISNVSQLPSGWASILWYNMLTSEPKNLKF
FLSPPAASWGQLSEVLSWQFSSVTKRGLNEEQLGMLADKLLGQKAQRNPEGLVPWTKFCK
SLSEKSFPFWLWIEAILDLIKRHLLSLWNDGCILGFVSKEREKALLTGKCPGTFLLRFSE
SSRDGAITFTWVEHDQYDKPVFHAVEPYTKKELSAVSLPDIIRTYKVMAAENIPENPLRF
LYPDIPKDKSFGKYYARASEASEPMDVESSSSTGYMKTELISVSEVHPSRLQDNMMPMSP
DVFGELKRSVAPVFQCWTGPKEIDAVASDLGEFLAEEFQMFENTPDLDRN
>STAT1.2 NM_001141285.1 NP_001134757.1 ssa21 NC_027320.1:12.218.693-12.235.684 ved STAT4
MAQWFQLQQLDSKYLEQVDQLYDDTFPMEIRQYLSAWIESHDWDMVATSVSLAAVRFHDL
LAQLDIQYSRFALENNFLLQHNIRKIKRNLQDHFQEYPLQMAMIICNCLKEEKKILASII
KKEDNVGSTPNNMVLDKQKELDNNVKDLRNRVQDAEHEIKSLEDLQDEHDFKKKTLQSRV
EQEVNGMAQLQAVRKEIREEEIVIRKMFIKLNITRGVVVNQISDILCLAEQIQFNLVTVE
VPEWKHRQQIACIGGPPNACLDQLQIWFTAVAEGLQQVRQQLKKLQELEQKYTYENDPIT
QGKSALEERALTLFKYLIVK
>STAT1.3 XM_014173037.1 XP_014028512.1 ssa25 NC_027324.1:10.235.279-10.263.141
MAQWFQLQKLDSKYLEQVDQLYDDTFPMEIRQYLSAWIESHDWDTVAASVSLATVRFHDL
LGQLDDQYSRFSLENNFLLQHNVRKIKRKLQDHFQEDPLHMAMTICKCLKEEKKILAALK
KEENVWSTQINVVLEKQKELDNKVKDLRNQVQDAEQKMKSLEDLQDDHDFKKKALQSRVE
QEVNGMTPVVQKEIREEEMVIRAMFIKLNITREVVVHQITDILNLAEQIQCTLVMEEIPE
WKHRQQIACIGGPPNACLDQLQTWFTAVAEGLQQVRQQLKKLQELEQKYTYENDPITQGK
STLEERALALFKTLIVNSIVVERQPCMPTHPQRPLVLKTGVQFTVKIRLLVKLQEFNYQF
KVKALFDKDVTERNTVRGFRKFNIFGTNSKVMNMEESNGSLAAEFRHLQLKEQKVTGNRT
NDGPLIVTEELHSISFETQLTHPGLQVNLETTSLPIVVISNISQLPRAWASVMWYNMLTS
ESKNLSFFLSPPQATWSLLSKVLSWQFSSVTKRGLNDEQLSMLGDKLLGREAVGNPNGLI
PWTRFCKNVNEKSFPFWLWIDGILDLIKRHLLCLWNDGCIMGFVSKEREKALLKDKEPGT
FLLRFSESSQEGAVTFTWVEHRNDGVHFHTVEPYTKKELAAVSFADILHNFKVMAAENIP
ENPLVYLYPRIPKDSALARYYSYPTEAAEPMELESESDAVYVKRELISVSELCAKLSQTN
LPSSGSAP
>STAT2.1 NM_001145424.1 NP_001138896.1 ssa12 NC_027311.1:58.670.995-58.782.875
MAQWEKLRQLDSVYLTQVDELYDGDAFPMDVRHYLAHWIEGQDWDRAARDHDVAMVLFQV
```

LLENLDIQHSRFVQGGESFLLQHNIRRFKHSFQRYQEEPYNLANIIQWFLEKEKEILQNA
ELAEQVQLLQVQQNPMETDSQRNMSADGRRRISQCMTHSECLRHKMKVLHYTDSYMRDHH
KRETEQMSAAGSTQQTDRFRKSMLSGMSVLLTLRDLLCVLVRGELVQWQRRQQKACIGAP
DSTCLDQLEKWFTTEAECLFQVRKFLKKLEELMGKVTYEHDPVKKQKPALQKRVDSLLTS
LLKSAFVVETQPSMPQGKGPLVLRTNVQFSVKTRFLVKFPELNHAMKVNVSMDREAPLVK
GYRRFNVLGTNSKALNMAESMSGGMVADFRHLTLKEQKSGGGGKGIHDLSLSVTEELHII
NFNTEFLLHDMSVSLETSSLPVVIISNSSQQQSAWASILWFNMLCLEPKNLLFFANCPAA
TWPQLGEMLSWQFLSSTTRGLEANQLDMIAHKLFGKQQSYDACKISWTKFSKENVPNTNF
TFCVWFDGILFMVKTHLENLWKDGSIMGFVSKGKEKTLLKKKRRGTFLLRFSESIRDGGI
TFSWVDYSPTGEPDVRSVQPFTKVDLCQIPFHEIIRNFQILEEENVPENPLLFLYPDTPK
VEAFALTEKSGADSQFFKYIRTKFVFVSKGNTLEAKSLMYSDTVEGEGSGPMNPRYGLGG
EAVEPSTFLPLPPSPDPMLPDAMVNPGEDLNPTFQEFLDDQSIYDCGLRILLCVTERHQL
HPTPGWYGGTHFNALLRRAGED
>STAT2.2 XM_014167157.1 XP_014022632.1 ssa22 NC_027321.1:28.174.986-28.181.318
MYACYYCSRAYKKPGMAQWEKLMQLESVYLKQVGELYDGDSFPMDVRHYLAHWIEGQDWE
HAAQDYDIAMVLFQVLLENLDIQYSRFVQQGEPFLLQHNICRFKHNFQRYQEEPYTLASI
ILWFLRKEEEIVHSAELAEQVQLLQVQQNPMETDSQWNIECKMTDLKKKVQSMLSGVGLL
LDDAEEVLFVLVREELVQWQRRHQKACIGAPDSTCLDQLEKWFTTEAESLFQVCKFLKLE
ELMGKVTYEHDPLKTQKPALQKRVDSLLNCLLKSFRPDHQQ
>STAT3.1 XM_014192884.1 XP_014048359.1 ssa03 NC_027302.1:60.568.918-60.598.070
MAQWNQLQQLETRYLEQLYHLYSDSFPMELRQFLAPWIESQDWAYAANKESHATLVFHNL
LGEIDQQYSRFLQENNVLYQHNLRRIKQHLQSKYLEKPMEIARIVARCLWEEQRLLQSAT
TAAQDGTASHPSGTVVTEKQQILEHNLQDIRKRVQDMEQKMKMLENLQDDFDFNYKTLKS
QGELSQDMNGNSQAAATRQKMSQLEQMLSALDQLRRQIVTEMAGLLSAMDFVQKNLTDDE
LADWKRRQQIACIGGPPNICLDRLETWITSLAESQLQIRQQIKKLEELQQKVSYKGDPII
QHRPALEEKIVDLFRNLMKSAFVVERQPCMPMHPDRPLVIKTGVQFTNKVRLLVKFPELN
YQLKIKVIIDKESGDVAAIRGSRKFNILGTNTKVMNMEESNNGSLSAEFKHLTLREQRCG
NGGRTNSDASLIVTEELHLITFETEVYHQGLKIDLETHSLPVVVISNICQMPNAWASILW
YNMLTNHPKNVNFFTKPPVGTWDQVAEVLSWQFSSTTKRGLTIEQLTTLAEKLLGPCVNY
SGCQITWAKFCKENMAGKGFSFWVWLDNIIDLVKKYILALWNEGYILGFISKERERAILS
PKPPGTFLLRFSESSKEGGITFTWVEKDISGKTQIQSVEPYTKQQLNSMSFAEIIMGYKI
MDATNILVSPLVYLFPEIPKEDAFGKYCRPEAAPEAELGDPCSTIQPYLKTKFICVTPTN
SGNTSDLFPMSPRTLDSLMHNETEANPGPMDSLTLDMELSSDVASPM
>STAT3.2 XM_014204116.1 XP_014059591.1 ssa06 NC_027305.1:37.543.543-37.577.365
MAQWNQLQQLETRYLEQLYHLYSDSFPMELRQFLAPWIESQDWAYAANKESHATLVFHNL
LGEIDQQYSRFLQENNVLYQHNLRRIKQHLQSKYLEKPMEIARIVARCLWEEQRLLQTAT
TAAQDGTASHPSGTVVTEKQQILEHNLQDIRKRVQDMEQKMKMLENLQDDFDFNYKTLKS
QSDNPPNSSPICSELSQDMNGNSQAAATRQKMSQLEQMLSALDQLRRQIVTEMAGLLSAM
DFVQKNLTDDELADWKRRQQIACIGGPPNICLDRLETWITSLAESQLQIRQQIKKLEELQ
QKVSYKGDPIIQHRPALEEKIVDLFRNLMKSAFVVERQPCMPMHPDRPLVIKTGVQFTNK
VRLLVKFPELNYQLKIKVIIDKESGDVAAIRGSRKFNILGTNTKVMNMEESNNGSLSAEF
KHLTLREQRCGNGGRTNSDASLIVTEELHLITFETEVYHQGLKIDLETHSLPVVVISNIC
QMPNAWASILWYNMLTNHPKNVNFFTKPPVGTWDQVAEVLSWQFSSTTKRGLTIEQLTTL
AEKLLGPCVNYSGCQITWAKFCKENMVGKGFSFWVWLDNIIDLVKKYILALWNEGYILGF
ISKERERAILSPKPPGTFLLRFSESSKEGGITFTWVEKDISGKTQIQSVEPYTKQQLNSM
SFAEIIMGYKIMDATNILVSPLVYLFPEIPKEDAFGKYCRPEAAPEAELGDPCSTIQPYL
KTKFICVTPCPSVFMDFPDSELLGNGIFPGTNSGNTSDLFPMSPRTLDSLMHNEAEANPG
PLALLLSVFPDSLTLDMELSSDVASPM
>STAT4.1 XM_014163961.1 XP_014019436.1 ssa21 NC_027320.1:12.263.661-12.303.660
MSQWKQIQQLQIKYQEQVGYLYDDNFPMDIRQVLSNYIESQDWDTAANHESMATVLLSNL
LSQLDRQCSQEQNFLQRHNLKFIYQQLQTKCKASPLLMAGVISSCLREERRIISTASIQE
QGPLEKSMQNSVAFERQKNMDNRVAVIRSSVQMMDQAVKYLEDMQDDFDFRYKTLQSRDP
VDRNTAVMKQEVTRLQEMLNNLDFKRKENLSKMSDVIKEIDALIVSQLNPELMEWKRRQQ
INCIGGPLLTGLDQLQNWFTLTAQSLFQMKRQLDKLGELILKVTYESDPIPLQRPQMEEQ
VKYLIYHLIKSSFVVEKQPCMPTHPQKPLIIKTGVQFTTKVRLLVKLPEVDYQLKVKTIF
DKDLPPGRVNRQFFILTTTTKVMDVEESSNGCLSVEFRHLLKEKKYVNGTKVNEGPVSVT
EELHSLSFEAQFNIQGINIDLETCSFPLVVISNVSQLPGGWASVMWYNLLTDEPRNLGFF
ASPQRASWGQLSEVLSWQFSTFAGRGLNREQLCMLGEKLLGQQVSQNECQVSWSKFCKEN
IHGKNFSFWMWLDSILELIKKHLLPIWNDNCIMGFVSKETERARLKDKEPGTFLLRFSES
HLGGITFTWVEQSDSGEAKFISVEPYTKSRLSALPFADIIRDYKVITDGEVPENPLKFLY
PDIPKDEAFGRLYNSQQNKVFPYIPSMLIPISTLQTTPPPCSSPEPPMSPGMFDMLTQHL
SPLEIETAMRSPYSE
>STAT4.2 XM_014173049.1 XP_014028524.1 ssa25 NC_027324.1:10.267.950-10.297.493
MSQWKQIQQLEFKYLEQVDYLYDDNFPMGIRQVLSDWIESQDWDMAANHESMATVLLSNL

LSQLDRQCSQEQNFLQRHNLKNINKQLQIKYKAYPLLMAAVISTSLREERHIIATASIQD
QGPLEKCLQNSVAFERQKNMDNRVAVIRSNVQLLDQAVKCLEDMQDDFDFRFKTIQSRDP
ADRNTELMKPEVTRLQEILNNLDFKRKENLSKMSDVIKEIDDLIASQLNPELMEWKRRQQ
INCIGGPLLTGLDQLQNWFTLTAQCLFQMKRQLDKLGELILKVTYASDPISLQRPQLEEQ
VKYLIGHLIKSSFVVEKQPLMPTHPQKPLIIKTGVQFTTKVRLLVKLPEVDYQLKVKTIF
DKDLPPGRVHRQFFILTSTTKVMDVEESSNGCLSVEFRHLLKEKKYVNGTKVNEGPLSVT
EELHSLSFEAQFNIQGINIDLETCSFPLVVISNVSQLPGGWASIMWYNMLTDEPRNLDFF
ASPLRASWGQLSEVLSWQFSTFTGRGLNREQLCMLGEKLMGQQASHNECQVSWSTFCKEN
IPGKPFSFWTWLDFILELIKKHLLPIWNDNCIMGFVSKETERACLKDKEPGTFLLRFSES
HLGGITFTWVKQSDSGEAKLVSVEPYTKSRLSALPFANIIRDYKVVADGEVPENPLKFLY
PDIPKDQSFRRHYNSQQSKVFPYISSTWIPISTLQ
>STAT5 XM_014158743.1 XP_014014218.1 ssa19 NC_027318.1:56.786.484-56.828.335
MAVWIQAQQLQGDALHQMQSLYGQHFPIEVRHYLAQWIEGQLWDSVELENPQDELKAKRL
LDSLVQELQRKAEHQVGEDGFLLKIKLGHYAGQLKSTYDRCPLELVRCIKHILHSEQRLV
QEATNAVGSSGGGGTMDSLSQRHQQINQTFEELRVSTQETENELRKLQHNQEYFIIQYQE
NLRIQAQLSSLSAVPAEERTQRETSLQAKRATVEAWLTREANTLQKYRLDLAEKHQKTLG
QLRKQQTLILDEELIQWKRRQQLAGNGGPTEGGLDVLQSWCEKLADLIWQNRQQIRRAEH
LTQQLPLPGPMEELLTKLNSDITDIISALVTSTFIIEKQPPQVLKTQTKFAATVRLLVGG
KLNVHMNPPQVKAVIVSEQQAKALLKNESTRNDSSGEILNNNCVMEYHRTTGTLSAHFRN
MSLKRIKRSDRRGAESVTEEKFTVMFESQFSVGGNELVFHVKTLSLPVVVIVHGSQDNNA
TATVLWDNAFAEPGRVPFIVPDKVLWPQLCEALDMKYKAEMHSARGLCDDNLIFLAQKAF
SSASINPEDYRNMTMSWAQFNRESLPGRNFTFWQWFDGVMELMKKHLKPHWNDGAILGFV
NKQQAQDMLMSKPNGTFLLRFSDSEIGGITIAWVAENPNKAGERLVWNLMPYTTKDFSIR
SLADRISDLNHLMFLYPDRPKDEVFSKYYTPPLSKAVDGYVKPQIKQVVPEFTTPNPEPS
SGNPTYMDQAPSPSVAHPINFAIFPPIRSDPMMDADGEFDLEDTMDVARHVEELLRRPMA
NQWDGQQS
>STAT5_L1 XM_014204113.1 XP_014059588.1 ssa06 NC_027305.1:37.582.155-37.674.290
MAVWIQAQQLQGDALHQMQSLYGQHFPIEVRHYLSQWLEGQLWDVIDLENPQEEFKAKRL
LDSLIQELQNKAEHQVGEDGFLLKIKLGHYASQLKSTYDRCPLELVRCIKHILYTEQRLV
REASNSSSPVGGMMDSMSQKYQQINQAFEELRLLTQDTENDLRKLQHNQEYFIIQYQESL
RIQAQLTSLATLPPADRQLREPTLLSKRATVEAWLTREANTLQKYRLDLAEKHQKSLQLL
RKQQTIILDDELIHWKRRQQLAGNGGPPEGGLDILQSWCEKLAETIWQNRQQIRRAEHLR
QQLPIPGPIEELLNDLNSTITDIISALVTSTFIIEKQPPQVLKTQTKFAATVRLLVGGKL
NVHMNPPQVKATIISEQQAKALLKNENTRNDSSGEILNNNCVMEYHQATGTLSANFRNMS
LKRIKRSDRRGAESVTEEKFTILFESQFSVGGNELVFQVKTLSLPVVVIVHGSQDNNATA
TVLWDNAFAEPGRVPFIVPDKVLWPQLCEALNMKYKAEVQSNRGLSEENLVFLAQKAFSS
SSINPDDYRGMTMTWSQFNRESLPGRNFTFWQWFDGVMELTKKHLKPHWNDGAILGFVNK
QQAQDMLMSKPNGTFLLRFSDSEIGGITIAWVAENPNKPGERMVWNLMPYTTKDFSIRSL
ADRISDLNHLLFLYPDRPKDEVFSKYYTPPLSKAVDGYVKPQIKQVVPEFTTTNPDPAAN
PTYMDHGASPVVSHPPNYGIYQPMSDSMLDADGDFDLDDTMDVARHVEELLRRPMENQWR
GQQS
>STAT5_L2 XM_014204115.1 XP_014059588.1 ssa06 NC_027305.1:37.600.971-37.605.524
MAVWIQAQQLQGDALHQMQSLYGQHFPIEVRHYLSQWLEGQLWDVIDLENPQEEFKAKRL
LDSLIQELQNKAEHQVGEDGFLLKIKLGHYASQLKSTYDRCPLELVRCIKHILYTEQRLV
REASNSSSPVGGMMDSMSQKYQQINQAFEELRLLTQDTENDLRKLQHNQEYFIIQYQESL
RIQAQLTSLATLPPADRQLREPTLLSKRATVEAWLTREANTLQKYRLDLAEKHQKSLQLL
RKQQTIILDDELIHWKRRQQLAGNGGPPEGGLDILQSWCEKLAETIWQNRQQIRRAEHLR
QQLPIPGPIEELLNDLNSTITDIISALVTSTFIIEKQPPQVLKTQTKFAATVRLLVGGKL
NVHMNPPQVKATIISEQQAKALLKNENTRNDSSGEILNNNCVMEYHQATGTLSANFRNMS
LKRIKRSDRRGAESVTEEKFTILFESQFSVGGNELVFQVKTLSLPVVVIVHGSQDNNATA
TVLWDNAFAEPGRVPFIVPDKVLWPQLCEALNMKYKAEVQSNRGLSEENLVFLAQKAFSS
SSINPDDYRGMTMTWSQFNRESLPGRNFTFWQWFDGVMELTKKHLKPHWNDGAILGFVNK
QQAQDMLMSKPNGTFLLRFSDSEIGGITIAWVAENPNKPGERMVWNLMPYTTKDFSIRSL
ADRISDLNHLLFLYPDRPKDEVFSKYYTPPLSKAVDGYVKPQIKQVVPEFTTTNPDPAAN
PTYMDHGASPVVSHPPNYGIYQPMSDSMLDADGDFDLDDTMDVARHVEELLRRPMENQWR
GQQS
>STAT5_L3 XM_014192796.1 XP_014048271.1 ssa03 NC_027302.1:60.501.000-60.559.701
MAVWIQAQQLQGDALHQMQSLYGQHFPIEVRHYLSQWLEGQLWDVIDLENPQEEFKAKRL
LDSLIQELQNKAEHQVGEDGFLLKIKLGHYASQLKSTYDRCPLELVRCIKHILYTEQRLV
REASNSSSPVGGMMDSMSQKYQQINQAFEELRLLTQDTENDLRKLQHNQEYFIIQYQESL
RIQAQLTSLATLPPADRQLREPTLLSKRATVEAWLTREANTLQKYRLDLAEKHQKSLQLL
RKQQTIILDDELIHWKRRQQLAGNGGPPEGGLDILQSWCEKLAETIWQNRQQIRRAEHLR
QQLPIPGPIEELLNDLNSTITDIISALVTSTFIIEKQPPQVLKTQTKFVATVRLLVGGKL
NVHMNPPQVKATIISEQQAKALLKNENTRNDSSGEILNNNCVMEYHQATGTLSANFRNMS

LKRIKRSDRRGAESVTEEKFTILFESQFSVGGNELVFQVKTLSLPVVVIVHGSQDNNATA
TVLWDNAFAEPGRVPFIVPDKVLWPQLCEALNMKYKAEVQSNRGLSEENLVFLAQKAFSS
SSINPDDYRGMTMTWSQFNRESLPGRNFTFWQWFDGVMELTKKHLKPHWNDGAILGFVNK
QQAQDMLMSKPNGTFLLRFSDSEIGGITIAWVAENPNKPGERMVWNLMPYTAKDFSIRSL
ADRISDLNHLLFLYPDRPKDEVFSKYYTPPLSKAVDGYVKPQIKQVVPEFTTTNPDPAAN
PTYMDHGASPVVSHPPNYGIYPPMSDSMLDADGDFDLDDTMDVARHVEELLRRPMESQWS
GQQS

SF5.3 Other transcription factors

>NFYA.1 XM_014147395.1 NP_001135288.1 ssa15 NC_027314.1:97.132.854-97.160.224
MEYTTATTSTGEQIVVQTSNGQIQQQVQGQPLMVQVSGGQLITSSGGQPIMVQAMGGGQGQ
TIMQVPVSGGQGVQQIQLQGGQQIQLQGGQQIQLQNGQMLQLGGQQIQLQNGQMLQLGGQ
QGQTQQIIIQQPQQAITAGQNQGQQIQLQQGQQLAQTADGQTIVYQPVNADGTVLQQGMI
TIPAGSLSGAQMVQTGGANTTTTNSGQQGTVTVTLPVSGNMMNTGGMVMMMPSGGTVPAM
QRIPLPGAEMLEEEPLYVNAKQYHRILKRRQARAKLEAEGKIPKERRKYLHESRHRHAMQ
RKRGDGGRFFSPKEREEMALALQQAELAAQAGEDDVAQMVRVS
>NFYA.2 XM_014134303.1 XP_013989776.1 ssa13 NC_027312.1:54.012.471-54.029.699
MEYTTATTSTGEQIVVQTSNGQIQQQTQGTMTAVQLQTEAPVLTASGQQVQTLQVQGQPL
VVQVSGGQLITSSGGQPIMVQAMGGGQGQTIMQVPVSGGQALQQIQLQGGQQIQLQNGQTI
HLGGQQIQLQNGQMLQLGGQQGQPQQIIIQQPQQAITAGQNQGQQIQLQQGQQIQLQQGQ
QIQLQQGQQLAQTADGQTIVYQPVNADGTVLQQGMITIPAGSLAGAQMVQTGGANTTTTN
SGQQGTVTVTLPVSGNMMNAGGMVMMMPSGGAVPAMQRIPLPGAEMLEEEPLYVNAKQYH
RILKRRQARAKLEAEGKIPKERRKYLHESRHRHAMQRKRGDGGRFFSPKEREEMALALAQ
QQAELAAQAAEDSVAQMVRVS
>NFYA.3 XM_014166883.1 XP_014022358.1 ssa22 NC_027321.1:27.442.640-27.452.769
MEQYTTATTSTGEQIVVQTAAGQIQQQVQGQPLMVQVSGGQLITSSGGQPIMVMGGQGGQG
QTIMQVPMSGAQGLQQIQLVQPGQIQLQGGQTLQVQGQQGQTQQFIIQQPQTAVTAGQNQ
GQQQITLQPGQQVAQTADGQTIVYQPVNADGSILQQGMITIPASSLAGAQIVQAGNSTNT
TNSSQGTVTVTLPGNMVNAGGMVMMVPGGGGSVPTMQRIPLPGAEMLEEEPLYVNAKQYH
RILKRRQARAKLEAEGKIPKERRKYLHESRHKHAMARKRGDGGRFFSPKEREEMALAMQL
QG
>NFYB.1a XM_014124522.1 XP_013979996.1 ssa10 NC_027309.1:71.648.409-71.700.001
MDGDSSTTDASQLGMSGEYMAGSHYVLQSQDDDGDESLHDHEEGNGSKENFREQDIYLPI
ANVARIMKNAVPQTGKIAKDAKECVQECVSEFISFITSEASERCHQEKRKTINGEDILFA
MSTLGFDMYVEPLKLYLQKFREAMKGEKGIGGVSVTEGLGEELTDDSFANQLPAGIITAD
GQQQNVMVYTTSYQQIPGVQQIQFS"
>NFYB.1b XM_014169900.1 XP_014025375.1 ssa23 NC_027322.1:35.750.908-35.783.684
MEGDGSTTDASQLGMPGEYMAGSHYVLQSQDDDCDESLHDHEEGNGSKENFREQDIYLPI
ANVARIMKNAVPQTGKIAKDAKECVQECVSEFISFITSEASERCHQEKRKTINGEDILFA
MSTLGFDMYVEPLKLYLQKFREAMKGEKGIGGISVTEGLGEELTDDSFANQLPAGIITAD
GQQQNVMVYTTSYQQIPGVQQIQFS
>NFYB.2a XM_014208086.1 XP_014063559.1 ssa07 NC_027306.1:40.991.492-40.994.683
MIVNQFLVSSNGNMEEDRSTTDASQLTLGISGEYMSGGYVLQSQDDDGEESLNDHDDGGM
KENFREQDIYLPIANVARIMKNGIPQTGKVTMIAKDAKECVQECVSEFISFITSEASERC
HQEKRKTINGEDILFAMSTLGFDMYVEPLKLYLQKFREAMKGEKGIPGVSVGEGLGEELT
DDSFTNQLPAGIITADGQQQNVMVYTTSYQQIPGVQQIQFS
>NFYB.2b XM_014153264.1 XP_014008738.1 ssa17 NC_027316.1:43.179.737-43.182.763
MEEDRSTTDASQLTLGISGEYMSGGYVLQSQDDDGEESLNDHDDGGMKENFREQDIYLPI
ANVARIMKNGIPQTGKIAKDAKECVQECVSEFISFITSEASERCHQEKRKTINGEDILFA
MSTLGFDMYVEPLKLYLQKFREAMKGEKGIPGVSVGEGLGEELTDDSFTNQLPAGIITAD
GQQQNVMVYTTSYQQIPGVQQIQFS
>NFYCa XM_014142803.1 XP_013998275.1 ssa14 NC_027313.1:78.725.782-78.765.010
MSMSADSFGAGGSDAQQNLQSFWPRVMEEIRNLTVKDFRVQELPLARIKKIMKLDEDVKM
ISAEAPVLFAKAAQIFITELTLRAWIHTEDNKRRTLQRNDIAMAITKFDQFDFLIDIVPR
DELKPPKRQEEVRQSVAPAEPVQYYFTLAQQPGQVQVQGQQQGQQQGQQATGQQTTTIQP
GQIIIAQPQQGQVLQGATMQQLQQVQVAQSQGTPITSAPMTMQVGDQQVQIVQASSQGQT
QTVQSGQTMQVMQQILTNSAEIQQIPVQLNTGQLQYIRLAQPVSGTQVVQGQIQTLGNTQ
QVQITQTEQGQQQFNQFTDGQQLYQIQQVTMPAGQDLSQPMFIQSTNQTADGQVTTQVSG
D
>NFYCb XM_014178432.1 XP_014033908.1 ssa27 NC_027326.1:29.300.001-29.354.350
MSMSADSFGAGGSDAQQSLQSFWPRVMEEIRNLTVDFRVQELPLARIKKIMKLDEDVKMI
SAEAPVLFAKAAQIFITELTLRAWIHTEDNKRRTLQRNDIAMAITKFDQFDFLIDIVPRD
ELKPPKRQEEVRQSVAPAEPVQYYFTLAQQPGQVQGQQATGQQAATIQPGQIIIAQPQQG

QVLQGTTMQQLQQVQVAQSQGTPITGGAPMTMQVGDQQVQIVQASSQGQTQTVQSGQTMQ
VMQQIITNSAEIQQIPVQLNNGQLQYIRLAQPVSGTQVVQGQIQTLGNTQQVQITQTEQG
QQQFNQFTDGQQLYQIQQVMPAGQDLSQPLFIQSTNQTADGQVTTQVSTD

NLRC5, RFX, RFXANK, RFXAP, CIITA

>NLRC5.1 XM_014149024.1 XP_014004499.1 ssa16 NC_027315.1:31.288.575-31.311.8356
MAMEDVEAEEDVQTVLTQETHELADILSYQDDGFLTRFYEMMDISSRQRLVQLASHRERI
LGLLNYFRTAEPAICRQFLQMVCMLCENMPMLLESRLMSVAGTVTKEVVENNSHTLVDEY
MASQECCLKRPRIDHIESYKAAVRRCLLQRWERVMQGVVKVSLEEAWVSLRHKSLVRPRD
RADRRLSPPELQEGAVEDRVTVDSLLGSEARVTLLLGQAGAGKTLLMHCLGQRWAQDAFP
SFHLLVLLEFRQLNLVARPLSLKELLFRFFLPPEGGEEEGAAVLDFILHNPEKICWIFDG
YDEFHTKITHSGSLRSPFDPQCPLPMAELISGLCSRRILPGCTLLITCRPRDVTDLESSV
DCIGELLGFNWRSVKEYAEQYFQDKDLDLKEKVVTHLLANHHLLTMCYLPALCHTCCVCL
DHIFSRGGSQSQALPQLPTTLTQVYLQILCAFLSRCPGRVTPLLQSHRAEVTQLSCMAMK
GLEDSRIVFLSEEVPPDLLDFVTKAGLLSQVDLTHEDGSKGQGYTFMHLTMQEFMGALHI
MTSKDITEAQLRKKFNLKTRWTTKSDPKTVFTDSLHLYVCGLAAPACSPYLFQLVVGAGA
VRWVKKRQALVLKLLRSLAASTNLTGPKVVELCHCIQETQDAQLAREVVGSRSCFELRNI
TLNPVDLDSLAFVVSSAGVGMGLDFGACSMELECLDILPSCQHIDYLIFRSRKYDDRFAD
KLSSILPRLPTLKRFEFICGSLTDVGAAKLARALESCPQITELNFSDNSLTDRGVGEIAD
LIPKLPSLASILLGKNGCSLEIIYTLLEKMTSCPSIQEVYTDGMKDINVLFFPSSDIKGS
KKNIGLTVSLLNCSLSTDQMTRLCQLLARSPGLTLLDLSGGHMKAGTLKALTDSLQKLNV
SKQIVLNDSHISVDGLMILTSFLSVCPDVVQVDIRLQDPVRVSMFFSRVIEKHKISRRLC
LTGCALRPPHLDRLCENLRDCSALTMLDISNNALENKGLKKLLDLLPQLSNIQEINVSEN
AVSMEGVVQLAGTLCSHRNMSEVNISHGGNKKLILKFLSSKRIQSTGLEQCKKLSLTHSD
IHPTDMNKLCGRLVQCPGLVELDFSHGSLKDDAIENLLKILPKMKSLQLLNMSHVQMSTD
GALLLVKSLIDCQRVRAVELRPQGEAFIKFVNMNAEQATCRLSQYTLSSGDVEKLSGILE
QCPHLSDLDLSSNLLRDEGVKRFVDFLPRLRIASSVNLDDNRLTQVGALYILNTVTTCEK
VVEVEVSLGTEKRSLIRFEQDSDCGKTLSLRECHFGADHLQRLAEILKSCAAQLVKLRLS
CNTMQREGLLALQNSLTTLSSLHTLDIRNNGLNVQVIEDLVKQLRCGHVQRCISIEETWI
TAEAAVNLVSCCLNLNPNIHTIRVNHTTVHITLEKCTNHTPTSGDSADMASSLSTVTISL
VGCAVQGHHLVSLQTVLQRCPLLQDLDLSHNSIGRVGAEFLCSVLPSLVSLRKLCVESKE
ASEDVVLLLAEGLLQAKSIESLNFSGHVISDRGAVVLTRTLQNLPNIRTINLSLCSGWTA
AGALDLVNGLGQCLSLEGISLDSAQLDQESTVSLAQGLHAMTSLKRLNLNRKVTMVTGSP
GEETTLVLLASLEGLRAMEEIELEGMRMSDKGVEELIKHLPTWTGLRKISLSDNYIGDQA
GERLVQVLANCTELEVLHLSRNKLSLACAAKMGQVLPTLTHLRVLDLSENPIGREGSVSI
SNALIFMKYLTKIHLTSIGTSELTGLAASLAYCVCAEDVSFAWNGCGDDVAVKLSEVLPQ
CQKLRRLDLESNSISATGAEALARSLQSCPSLEVIRLWRNSISTSDAQRLRQREKRLNFF
ST
>NLRC5.2 XM_014124224.1 XP_013979700.1 ssa10 NC_027309.1:79.263.529-79.290.760
MEAEEDVQTVLTQETHELADILSYQDDAVLAILNKMMDSRARERLVRLASHRERILGLLN
YFRTTDPAICRQFLQMVCMHCDMPMRLESRLMSVAGSVTSDPWPTPARETLSLCDPEVIE
NNSHTLVHEYIPSQGWCAKRPRIDHVESYKSAVRRFLLQRWERVMQGVVKEVRLEEAWVS
LRHRTHVRPRDRADGALSPSELPQGDEEGAVEDRVTVHSLLGSEAQVTLLLGQAGSGKTL
LMHCLGQKWTQGAFPSFHLLILLEFRQLNLVARPLSLKELLFRFFLPPEGGEEECTAVLD
FILENPEKICLIFDGYDEFHTKITHSGKLSSPFYPLCPLPMAELISGLCSRRILPGCTLL
ITCRPRDVTDLESSVDCIGELLGFNQRSVKEYAEQYFQDKGLDLKEKAVSHLLANHHLLT
MCYLPALCHICCVCLDHIFSSGGSQLLPQLPNTLTQVYLQILCAFLSRCPGSSTPLLQSH
RAEVTLLGRLAMRGLEGSKIVFLSEEVPPDLVDFATKAGLLSQVDLTHEDGSKGQGYTFM
HLTMQEFLGALHIMTSEDITDAQLRKKFNFKTRWTTKSDPKTVFTDSLHLYVCGLAAPAC
SSYLIQLVRGVGAVGWVKKRLALVRKLLRSLAMSTNLTGPKVVELCHCVQETQDAQLARE
MVGSRPCFELRNITLNPVDLDALAFVVSSAGVGIGLDFGACSMELECLDILPSCQHIDFL
IFRSRKYDDRFADKLSSILPRLPTLKRFEFICGNLTDVGAAKLARALESCPLITELNFSD
NSLTDSGVGEIADIFPKLPSLASVSLGRNGCSLESIYILLEKITSSPSIQGLYAEGMKDG
SVLFVPSLDMKCSKDKTGLTVSLLNCRFTTDQMNRLCQLLARCPGLSVLDLSGGDIKADT
LKALTDSLQELNVSKQIVLNEIPISVDGLMVLTSFLSVCPDVVQVDISLQDPVRASILFA
GGIEKQHPEMSKKLCLIGCGLRPPHLDHLCENLMDCSALTLLDISNNSLGNKGLKKLLDL
LPQLSTIQEINVSENAVSMEGVVLLADTLCSHRNMSEVNVSHGGKKLFLKFHSSKRIQSE
ALRTSPDMEQDKKLSLTHSDIQPTDMTRLCRRLVQCPGLLKLDFSHGSLKDDAIENLLNI
LPKMTSLQLLNLSHVQMSTDGALLLVRSLIDCQRVRAVELRPQGEAFIKFVNMKVEQATC
RLTQYTLSSRDVEKLSGILEQCPHLSDLDLSSNLLRDEGVKSFVDSLPRLRIASSVNLND
NRLTQMGVLYLVNTVTTCEKVAAVEVSLGTEERSLIRFEQDSDCGKTLSLRECHFGADHL

QRLAEILTRCAAQLVKLRIRNNGLSVQVIEDLVKQLRCGHVQRYISIEEPWITAEAAVNL
VSCCLNLNPNIHTIRINKSTVHITLEECQNPTATSGGTSTDMSGSLSTVTISLVDCAVQG
HHLVSLQTVFQSCVLLQELDLSLTSIGRVGAELLCSVLPSLASLRKLSLESKETSEDVVL
LLAEGLLQAKSIESLNLSGHVITDRGAVALTRTLQNLPRLRTINLSLCYGWTPASALVLV
RGLGQCLSLEGISLDSVQLDEESTVCLAQGLHAMTSLKRLNLNNKVTMLTGSPWEGATLV
LLSSLEGLRGMEEIELEGMRMSDKEVEELIKHLPTWTGLRKISLSGNCISDQAGERLVQA
LTTCTALEELNLSRNNLSLACAVKMGQVLPTLTHFRVLDLSENEIGTKGSVSISKALISM
KYLTKIHLTSIGTSELACLADSLAHCVCAEDVSFAWNDCGDDVAVKLAEVLPQCQKLRRL
DLESNRISTIGAEALARSLQSCPSVEVIRLWRNVISASDAQKLRQREKRLNFSST
>CIITA.1 XM_014183310.1 XP_014038785.1 NW_012347531.1:5.494-24.308
MCQDMEAGLSLASHYVDVRLVQRQIQIGSGKNGSKCLEKDLVAIGDGERRRDSLARSQVF
ESSAGAKPKRSVVLLGNAGMGKSTLIKKLCVDWSDGLLPQFDFVFLLDGKALTLPPEPTY
SLQSLLLHYSSSSSPIPSTCPHHRDVFNQVLSVPERVLVIFDGFEVVRDLEGLLQCPADD
SKGETYSVRQLFSGLLQRKLLPGCSLLLSARPRGTVSGLLRRADSLLELSGFSPPDVERY
LGQYFSGQSQEVPTSGLTSDPIPVPVPTSDPASDPIPVPVPTSDPASDPIPVPAPVPVPA
PVPPRGRRRPQGECLDLAQRFAVGLLFRNKAELRDLATLDTGAMKVVMAKRAAVISHLEN
LHHGDLSPARLLEACHCVYETGDVHLVRHLVRNLPEVLSFQGVPLCPADTFVVWNLLDQC
RTLRRRFCLGLEDTGLRMTGLKLLTGLNNIHSYRYWYCYTDRPDTPRGRRRPQGECLDLA
QRFAVGLLFRNKAELRDLATLDTGAMKVVMAKRAAVISHLENLHHGDLSPARLLEACHCV
YETGDVHLVRHLVRNLPEVLSFQGVPLCPADTFVVWNLLDQCRTLRRRFCLGLEDTGLRM
TGLKLLTGLNNIHSYRACIADTITLWEELEQSGEEELLKAAVSKLTLNPFRATQVSHVKH
LSTLVNIHTSRRLSESLSDGVLGEGIPAVRDLHKLEFELGPVNGPLALPKLLELLPALHS
LHHLDLENSKLGDSGAQGLAEAVVSLSSLQIINLSQNCIGDQGIGSLAPALSTLSSLHCL
SLYSNVISDGGAESLAAVLPQMTSLSDLDVKYNSFTALGAQSLSSSLRDCPWIKSLGMWN
VCIPYGVLERLQQQDPRIQLL
>RFX5.1 XM_014177217.1 XP_014032693.1 ssa27 NC_027326.1:11.825.492-11.840.435
MTEDRLKADPSKREGLDSEEGDTEPSLLLQKLKSNISKNVQSRVDVILQDVQRFSDNDKL
YLYLQLPSGPSSGEKSLAMAPLAQLRRWCISASMSALEERTLPSSSSDPSSFNTADQLHT
CNWIRSHLEEHADTCLPKQDVYETYKRYCENLQHRPLSAANFGKIIRDIFPNIKARRLGG
RGQSKYCYSGIRRKTVLNMPLLPNLDLKNDPSELTELVQTYKQEVTEAACELICDWAQKI
LKRSFDTVVEIARFLVQEHIVNPRCSQAELVTSAAMAGGPAKPHKVIKKNPVPSKGGGPE
TEGSGSEAKRDKDVGDQSLPGKLQSSDKPIKGAESVRPGGRESQVEALMKHLPRILPRSS
IPEKSQLSVRSSPPSLAPKDTGGVKVITMTALPQQQGGALPVMILPQSVSLSYPDREKAP
PNTMAPVAPMSVVQRARTAGKRAPEAASGGPGPGGTPAKRKRGRPRKPRPEDTAPPQPPN
PPLPSVNQAPIMNSLTGGVIQKACSSSSSQVVEVVFQDQQALVLGQLHSVADTGDPEHRG
VVLETDPRPVLLLSGASHTNWDMGRAMVEVIQRAPRPPTIITKNNNNSQSTPQHRLPLPT
VLEDRGEVEITLTPMAPSDNLPNPTAQARSEGGPTPDDSSKEPSPGLPCPRD
>RFX5.2 XM_014141621.1 XP_013997098.1 ssa14 NC_027313.1:60.828.513-60.843.570
MEWSREQALWNSAPTTSIRPRGLRVCLPPLCEQTMTEDRLKADPSKREGLDSGEGDTEPS
LLLQKLKSNISKNIQGKVDVILQDVQRFSDNDKLYLYLQLPSGPSSGEKSSSSSSSSDPS
SFNTADQLHTCNWIRSHLEEHVDTCLPKQDVYETYKRYCENLQHRPLSAANFGKIIRDIF
PNIKARRLGGRGQSKYCYSGIRRKTVLNMPLLPNLDLKNDPSELTELVQTYKQEVTEAAC
ELICDWAQKILKRSFDTVVEIARFLVQEHIVNPRCSQAELVTSAAMAGGPAKPHKVIKKN
PVPLKGGGPETEGSSSDAKRDKEVGDQSSPGTLQSSDKPTKGAESVRPGGRDLQVEALMK
HLPRILPRSSVPEKSQGGALPVMILPHTVSLSYPEKSPPVTMAPVAPTSVVQRARAVTKR
ALEAPTATSGGPGPGGMPAKRKRGRPRKPRPEDTTSPQLPPPSLPSVNQAPIMKSLTGGV
IQKACSSSSSSQVVEVVFQDQHAVVLGQLHSVADSGNPEHRGVVLETDPRSLLLLPGTSH
ANWDMGRAMVEVIQRAPRPPTTKNNNNSQSTPQHRLPLPTLLEDRGEVEITLTPMEPSDD
LPTPTSQASSEGRPAPDDSAKVERP
>RFXANK.1 NM_001140973.1 ssa16 NC_027315.1:38.347.886-38.350.460 pseudo
MDGTDGGEIPDLAASLHIFSDDLALESTTCLPGEARGERLNGVMATTENMGVDEEESLLK
YSTTLTNRQRGNEVTVRPATLDILSIHQLAAQGEVLQVATHLSKDSSLLNRQDERGLTPL
MWAAAFGEKAMVDFLLENGADPSTIAWERESALTLASSGGYAVIVKRLLEHGVDINAYDW
NGGTPLLYAVRGNHVRCAEALLDKGADMNIEADSGYSPMALAVALGHKQVQKVLEDHILK
LLKKKA
>RFXANK.2 XM_014128069.1 XP_013983544.1 ssa11 NC_027310.1:51185525-51190841
MDKGLVPGRTSSVSEMEGICVIPDMSGIKSEHPVGSVDNTGAQNVAMGIKFILPNRFDMN
VCSRFVKSLNEEDSKNIQDQVNSDLEVASVLFKAECNIQTSPSPGIQVRHVYTPSTTKHF
SPIKQSTTLTNKHRGNEVSSTPLLVHSLSIHQLAAQGEMVFLASRIEQETVINLQDEEGF
TPLMWAAAHGQIAVVEFLLQNGANPNLLAKGRESALSLACSKGYTDIVKMLIDCGVDVNE
YDWNGGAPVLYAVHGNHVPCVEILLERGADPTIESDSGFNAMDMAVAMGHRNVQQVMEAH
LLKLLMGMRE
>RFXAP.1 XM_014129757.1 XP_013985233.1 ssa11 NC_027310.1:87.363.503-87.371.928
MSEEESSTSSGNNKDSSISLLLTKDGQTYYVDKSGVVDSRNVVTPQDSYNNNMFSYDMDD

ADEESDVLDTSDPRDSAASPEELNEEDVFGENENVSKTCTYDGCTETTTQVAKQRKPWMC
KKHRNKMYKDKYKKKKSDQAMSSGKQDESLEERPVSVNKQRLGTMGDRPARPSLIEQVLN
QKRLSLLRSPEVIRFLQQQQRLLTTQSHSQSQPDFQGC
>RFXAP.2 XM_014198370.1 XP_014053845.1 ssa04 NC_027303.1:80.495.837-80.499.234
MSEEESSTSAGNNNKDSSILLTKDGQTYYVDDCGVVDSRNVVTPQDPDNNNMFSYEMDDA
DEEESDILDTSDPRDSAASPEELNDEERYGDNDNISKTCTYDGCTETTSQVAKQRKPWMC
KKHRNKMYKDKYKRKKSDQAMSTGKQDESLEERPVSVNKQRLGTVGDRPARPSLIEQVLN
QKRLSLLRSPEVIMFLQQQQRLLANQSLSQSQPHFQGC

NF-kB

>NFKBp65a XM_014206784.1 XP_014062259 ssa07 NC_027306:11.733.819-11.738.213 no data
MDGMYGWGQPPLNQGNSFIEIIEQPKPRGMRFRYKCEGRSAGSIPGEKSNDTTKTHPAIK
VHNYNGPLRVRVSLVTKNPPHKPHPHELVGKDCKHGYYEADLQERRVHSFQNLGIQCVKK
KDVNEAVSCRLQTQNNPFNIPEAKVWEEEFDLNAVRLCFQASITLPTGELHPLEPVVSQP
IYDNRAPNTAELKICRVNRNSGSCRGGDEIFLLCDKVQKEDIEVRFFQDSWEGKGTFSQA
DVHRQVAIVFRTPPFYDTNLTEPIRVKMQLRRPSDREVSEPMDFQFLPSDPDEYRLIEKR
KRTEGMFQNLKLGSISVAMPAERPFNTARRTVAAKPVASQPVNPVAPPRATSVKPQPYYD
SPQPGQLFQTQPKAEPSSSTPAETWKFLNSLTLDSQHKATPVARFTTNPQASAATSQAFP
TVNLSDLHGFTFSTFASPQEPVSAAATPEPTSTFGVQDSQFRVDGPMVDEELPEFPSFSE
VHQSGTLDSINIDDFQAMLVQSGLAGEGPGNSRVSVSQAPCHLPATNPVVNNSCGGSTWM
NYPNSIVNLLQSEGMVDSSPGNSSQPAVLDDLDVFSSIDEDRLMSILNSGN
>NFkBp65b1 XM_014156036.1 XP_014011511.1 ssa18 NC_027317:47.623.987-47.631.102 p65-like
MRFRYKCEGRSAGSTPGEKSNDTTKTHPVIKVHNYNGPLRVRVSLVTKNPPHKPHPHELV
GKDCKHGYYEADLQERRVHSFQNLGIQCVKKEDVAEAVSCRLQTQNNPFNIPEANVWEEF
DLNAVRLCFQALITLPSGELFALEPMVSQPIYDNSHQAARYGAHLSPSLQCSDVGLFQCS
LTGQVFLMEGEVLYKTVQWDESLLHSTGRTHAGALFRSSGVCPSATPPTL
>NFkBp65b2 XM_014155658.1 XP_014011133.1 ssa18 NC_027317:47.645.715-47.655.880
MDGIYGWEQPPLNQGNPFIEIIEQPKQRGMRFRYKCEGRSAGSTPGEKSNDTTKTHPAIK
VHNYNGPLRVRVSLVTKNPPHKPHPHELVGKDCKHGYYEADLQERRVHSFQNLGIQCVKK
KDVAEAVSCRLQTQNNPFNIPEANVWEEFDLNAVRLCFQASITLPTGELFALEPVVSQPI
YDNRAPNTAELKICRVNRNSGSCRGGDEIFLLCDKVQKEDIEVRFFQDAWEGKGTFSQAD
VHRQVAIVFRTPPYCDTNLTEPIRVKMQLRRPSDREVSEPMDFQFLPSDPDEYKLMEKRK
RTEGMLQNLKLGSLMSGAMPAETRPFNIARRTVTAKPAASQPVMNQVAPPSAVSVKPQPY
YNGPQPGQLFQTQPKAEASTTAETWKFLNSLTLDSQPKATPVASFITNPQASVATAASSQ
DFPTVNLLDLHGYAFHTFASPQEPVSAAATTEPTSTFGVQGSQFKVDEELPEFPSFEAQP
TGTLDSINFDDFQAMLGQSCLAGEGPKSSEASAPQAPCHLPSTNTVANNAAQNQADPANH
HISCGGSTWMNYPNSIVNLLQNEGMMDSSPGNASQPAALDDLDVLSSMDEDHLMSILSSG
NQYTFVSGHQT
>NFKB_p105a_p50a XM_014199784.1 XP_014055259.1 LG05 NC_027304.1:34.546.596-34.582.648
MAEEEAYLPHPQYYDIEPIWDPLNFPPMSLTNSLRTADRPYLQIIEQPKQRGFRFRYGCE
GPSHGGLPGASSEKNRKSYPQVKICNYQGLARVVVQLVTNSKDAHLHAHSLVGKQCDKGI
CITDLQPKDCSISFPNLGILHVTKKNVSKTLEDRMTEAYRMGYNCGIVIHPEIDTIQGEV
RIPRELTDHQRSMICSAATKQAKEMDLSVVRLMFTAFLPDSDGGFSRRLDPVISDPIFDS
KAPNASNLKIVRMDRTAGCVTGGEEVYLLCDKVQKDDIQVRFYEDDETGLTWEAFGDFSP
TDVHRQFAIVFKTPKYRDLNLQKPTSVFVQLKRKSDNETSEPKPFTYHPQIIDKEEVQRK
RQKTLPNFQDYNGQGGAGGMYRGTGGGSATGGGPGSGGGGYFQAYSTYNNYGTGYNAGFS
PGMSGGGAGIKHATQSRAEDSGDDSDMGDDPSSGAVVADRAQSEEDASAGETSEERGAGV
ELGPGDRTGECVLEARLVDVAERQAEALFHYAVTGDVRYLMAPQRHLMTAQDENGDTGLH
LGVIHSQTDAVRSLAQVLSALPGEEVLNMRNDLYQTPLHLAVITQQKEAAEALVLAGADV
TLSDRNGNTALHLATQQKEGGMVGFLLRHREVVELVDLPNTAGFCSLHLAVLANSLCSLR
DLLVSGGNVEVQERSCGRTALHLATELDNVSLAGCLLLEGNADVDCCTYNGSSPLHIAAG
RGSVKLTALLMAAGANPHKENFEPLFFREDDCYVDEEEEQDEGYIPGMTPLNMAATPEVL
EILNGKEYKPETTIPVFIPPQGDMRSLGSDTKRALCQALECQGGSWESLANTLGLGILNS
AFRLSPSPASTLLDSYEVSGGMVKDLLEGLRTVDNSTALTVLQGALCETEQAPLAPQSTT
ELLGRVQDLKLDGQEDSGVCDSGVELSTA
>NFKB_p105b_p50b XM_014212190.1 XP_014067665.1 LG09 NC_027308.1:65.482.780-65.505.283
MAEEEPYLPHQPYNFDIDPLWDPLNFSPMSHSNSLRTVTADGPYLQIIEQPKQRGFRFRY
GCEGPSHGGLPGASSEKNRKSYPNIKICNYQGLARVVVQLVTNSKDAHLHAHSLVGKQCD
KGICIADLQPKDSSISFPNLGILHVTKKNVSKTLEDRMSEAYRMGYNCGIVIHPEMDAFQ
GEFRIPRELTDHQRNLISSAASYQAKEMDLSVVRLMFTAFLPDSDGGFSRRLDPVISDPI

```
FDSKAPNASNLKIVRMDRTAGCVTGGEEVYLLCDKVQKDDIQVRFYEDDETGLTWEAFGD
FSPTDVHRQFAIVFKTPKYRDLNLQKPTSVFVQLKRKSDNETSEPKPFTYHPQIIDKEEV
QRKRQKTLPNFQDYSGHGGAGGLYRGPGGGGPATGGGGGFFQGYSTYSNYGTSYSSGFSP
GMSGGGAGIKHAPQGRAEDSGDDSDMDDDPASGAVVGVRAKSEDASAGETSEERGAGVEL
GPGDRTGECVLEARLVDVAERQAEALFQYAVTGDVSYLLAPQRQLMTAQDENGDTGLHLG
VIHSQTDAVRSLAQVLSALPGEEVLNMRNNLYQTPLHLAVITQQKAAAEALLLAGADVTL
SDRHGNTALHLAAQQEEGGMVGFLLRHREIVELVDLPNAAGLCSLHLAVLANSLSSLRDL
LVSGGSVEVQERSCGRTALHLATEQDNISLAGCLLLEGNANVDCCTYNGSSPLHTATGRG
SVKLTALLMAAGADPHKENFEPLFFREHDCCVDEEEEDEGYIPGTTPLNMAATPEVLEIL
NGKEYKPETDVFVPPQGDMQSLGSDTKRALCQALECPEGGWESLAHILGLGILNSAFRLS
SSPASTLLDSYEVSGGKVKDLLEGLRTVGNCSALTVLEGALCEAEQAPQSTTKLLERVCD
LKLDGWEDSGVCDSGVELSTA
```

SF5.4 Interferon response factors IRF1 and IRF9

```
>IRF1a XM_014136758.1 XP_013992233.1 NM_001123645.1 NP_001117117.1 ssa13
NC_027312.1:54.674.198-54.678.695
MPVSRMRMRPWLEEKIESNSISGLVWVDKDNKIFSVPWKHAARHGWDLNKDACLFKQWAM
HTGKFIQGETKTDPKTWKANFRCAMNSLPDIEEVKDKSINRGSGAVRVYKMKNIYSKPNN
KRSKANNVKKNKKGSQIKTGGMAYSETNCPENLNTNTHLQEDSMTQESIVDSTGNLGDFT
FAPECSTNVEIGPDSTNNFYASFQVSPDHSTDYEDGHQETLIGMTHHWEQGSVNDKGFQS
NEVGTAESFDTAESYHSQESQWSDNSETEIELRLYTELSSGLPIIDDILSYTDYWTLNNN
TSSYPQQITCPL
>IRF1b NM_001252364.1 NP_001239293.1 ssa04 NC_027303.1:69.623.751-69628218
MPVSRMRMRPWLEDKIESNSISGLVWLDKDKKIFSIPWKHAARHGWDLNKDACLFKQWAM
HTGKFIQGETTPDPKTWKANFRCAMNSLPDIKEVKDKSINRGSGAVRVYKMLNVSTKPNN
KRSKAKDAKKNDKGLKIKTEEMDYSATHCLEDRNTNTHLQEDRKIQENKVDSSDNLGETI
TAASYLDGSVNDPDVPDFITSVEIGPDSINYYSSFQVSPDHSTDYEDLNEETLIEIAKHW
EQLELPGSVNSKGFLSNEEATVESYNTAESNHSPESQWSDNSGSEIQLRLYTELSPGLPM
AEDLVSYTDHWALNNTLNNSTTSYLQQISCPL
>IRF9a XM_014159557.1 NM_001173719.1 NP_001167190.1 ssa19 NC_027318.1:68.799.550-
68.807.766
MASGRVRSTRRLRSWMVDQVTSGKYPGLIWDDDAKTMFRIPWKHAGKQDFRSEEDGAIFK
AWAVFKGKLSDGGRVDPASWKTRLRCALNKSPEFREVPERSQLDISEPYKVYCLVPMNEQ
VLGNVKLKGRARAGGRKRRSSDSDSEEEVKEEEVVVKQMKEEVITAPITMSVQEVEESVL
TIQQDQFDQPLVILKSDGTVDELQVNVTIETVPPPGARDSFHVLVKYIGQEVLKREVIGS
DVRIAYLPSSPVPPTLMMGGFPRIPLPESPSTLTSSPGIGPRLQALSTLLPFMEKGVILT
STGAGIYAKRYCQGRVFWTGPHSTTTGPHKMNRAVEPVLLFDREAFKMELDHFRSYGGDP
PQCGITLCFGEEFSATEDPSSKLIITQITLPWAQQQVKEAEDFRESMTYLRDIASQSGDV
TINLVPVPYP
>IRF9b XM_014181409.1 XP_014036882.1 ssa29 NC_027328.1:25.025.478-25.031.127
MASGKIRSTRRLRAWMVEQVSSGKYPGLIWDDDDKTMFRIPWKHAGKQDFRSEVDGAIFK
AWAVFKGKLSEGGHADPASWKTRLRVALNKSPEFREEPERSQLEISEPYKVYRLVPINEQ
ALGSVDMKVQARAGGRKRRSCDSDIEVEEEVVKVKQMKEVTTSLPITMSVQEIEESLLTI
QLDQVEPSVMVKSAGTVNEIQVNFTIETVPPPGAQDSFHVLVKYMGEEVLKRGVMGSDVR
IAYLPSSPVPPTLMVAGFPRIPLPDPPSTLTSSIGPQFQALSTLLPFLEKGVILTSTRTG
VYAKRYCQGQVFWTGPHSATAGPHKMNHAVEPVRLFNREAFRMELDHFSSYGGDPPQCGF
TLCFSDKEDPSSKLIITQITLPWAQQQVKEAEDFRESMTYFRNITSESGEVTINLVSGSL
LSEILGTSLP
```

SF5.5 Deduced MX amino acid sequences

```
>MX1 XM_014133086.1 NP_001133390.1 ssa12 NC_027311.1:66.776.321-66.785.731
MNYTLNQHYEEKVRPCIDLIDSLRSLGVEKDLALPAIAVIGDQSSGKSSVLEALSGVALP
RGSGIVTRCPLELKMKRKKEGEEWHGKIRYQDREEEIEDPSDVEKKIRKAQDEMAGVGVG
ISDDLISLEIGSPDVPDLTLIDLPGIARVAVKGQPENIGEQIKNLIRKFITKQETINLVV
VPCNVDIATTEALKMAQEVDPQGGRTLGILTKPDLVDKGTEEMVVDIVHNEVIHLTKGYM
IVKCRGQKEIMEQVSLTEATEREKAFFKEHLHLSTLYDEGHATIPKLAEKLTLELVQHIE
KSMPRLKEQIEEKLEETRTTLEKCGTGPPEDPKERQYFLIDKVTLFTQDVINLSTGEELK
SGDINIFSTLRTEFGKWKAQLDRSGKNFNKKIEKEVADYEKTYRGRELPGFINYKTFEVM
```

VKDQIKQLEEPAVKKLKELSDVARKAFILLAQNSFTGFPILLKTAKTKIETIKQEKESTA
ESMLRTQFKMELIVYTQDITYSSSLRKRKREEEELEEGELVKNPSLSFGSQKVLSVFSVR
STVNGHDNHAALREMMLHLKSYYNIASQRLADQIPMVIRYLVLQEFASQLQREMLQTLQE
KDNIEQLLKEDIDIGSKRASLQSKLKRLMKARSYLVEF
>MX2 NM_001123690.1 NP_001117162.1 ssa12 NC_027311.1:66.798.392-66.803.823
MNNTLNQHYEEKVRPCIDLIDSLRSLGVEKDLALPAIAVIGDQSSGKSSVLEALSGVALP
RGSGIVTRCPLELKMKRKKEGEEWHGKISYQDHEEEIEDPSDVEKKIREAQDEMAGVGVG
ISDDLISLEIGSPDVPDLTLIDLPGIARVAVKGQPENIGEQIKRLIRKFITKQETINLVV
VPCNVDIATTEALKMAQEVDPEGERTLGILTKPDLVDKGTEETVVDIVHNEVIHLTKGYM
IVKCRGQKEIMERVSLSEATEREKAFFKEHAHLSTLYDEGHATIPKLAEKLTLELVHHIE
KSLPRLEEQIEAKLAETHAELERYGTGPPEDSAERMYFLIDKVTAFTHDAINLSTGEELK
SGVRLNVFSTLRKEFGKWKLHLDHSGENFNQRIEGEVADYEKTSRGRELPGFINYKTFEV
MVKDQIKQLEEPAVKKLNQISDAVREVFLLLAQSSFIGFPNLLKSAKTKIEAIKQVNEST
AESMLRTQFKMEMIVYTQDSTYSHSLSERKREEEDDRPLPTIKIRSTIFSTDNHATLQEM
MLHLKSYYRISSQRLADQIPMVIRYLVLQEFASQLQREMLQTLQEKDNIEQLLKEDFDIG
SKRAALQNKLKRLMKARSYLVEF
>MX3 ssa12 NM_001123675.1 NP_001117147.1 NC_027311.1:66.816.619-66.828.660
MNNTLNQHYEEKVRPCIDLIDSLRSLGVEKDLALPAIAVIGDQSSGKSSVLEALSGVALP
RGSGIVTRCPLELKMKRKKEGEEWHGKISYQDHEEEIEDPSDVEKKIREAQDEMAGVGVG
ISDDLISLEIGSPDVPDLTLIDLPGIARVAVKGQPENIGEQIKRLIRKFIMKQETINLVV
VPCNVDIATTEALKMAQEVDPEGERTLGILTKPDLVDKGTEETVVDIVHNEVIHLTKGYM
IVKCRGQKEIMERVSLTEATEREKAFFKEHAHLSTLYDEGHATIPKLAEKLTIELVHHIE
KSLPRLEEQIEAKLAETHAELERYGTGPPEDSAERMYFLIDKVTAFTHDAINLSTGEELK
NGVRLNVFSTLRKEFGKWKLHLEHSGENFNQRIEGEVADYEKTYRGRELPGFINYKTFEV
MVKDQIKQLEEPAVKKLKEISDAVRKVFLLLAQSSFIGFPNLLKSAKTKIEAIKQVNEST
AESMLRTQFKMELIVYTQDSTYSHSLSERKREEEEDEDKPFSEIRSTIFCTDNHATLQEM
MLHLKSYYSIASQRLADQIPMVIRYLVLQEFASQLQREMLQTLQEKDNIEQLLKEDFDIG
SKRASLQSKLKRLMKARSYLVEF
>MX4 XM_014214722.1 XP_014070197.1 ssa09 NC_027308.1:117.838.751-117.853.817
MHRPGAGSEDEERYRDGMQSGVFYSHLDRQVRPFIELIDFLRSIGIEKDLALPAIAVVGD
QSSGKSSVLEALSGVALPRGSGIVTRCPLELKLRKSFGGKWKAKISYQGVVETFEDPSLV
EIHVRTAQNTLAGDGVGICDDLITLEITSPDVCDLTLIDLPGITRVPVTGQPEDIGDQIR
RLILKFIKKQETINLVVVPCNVDIATTEALRMAQSVDPEGARTLAILTKPDLVDKGAEPD
ILKIVNGQVVHLNKGYIIVKCRGQNDINQKISLADATRLEMEFFKNHHHFSPLLEQNKVT
TQCLATKLTQDLVDHIKTSLPYLTDQIREHLETVKTELKKYSTGPPLERKKMGPYLTERL
MDFIDKIHELCRIGNSSEKNLYTFLRPVFQQWDSYLSNTKGSFLNKVEAMIKNYDKEHRG
RELITFSDYCVYEHAVQKHILGLQEPALDVLKAIRDMVQAEFRHVCEACFKSYPQLRCLA
LTKIDEIQMKQEAKVEKRIKEYINMERLVYTQDSIFVKGLKDHKDQLKEAFEEEHFYDPE
EIEDITATFNCTAFDSRKLTTDKLGVYYEIVYQRLADYVPMLI
>MX5 XM_014174614.1 XP_014030089.1 ssa25 NC_027324.1:47.104.856-47.121.653
MSYDDGPSMFQDQLAEKVRPFIDLVDNMRSIGIDKELPLPTIAVVGDQSSGKSSVLETLS
GVALPRGTGIVTRCPLLLQLCNDRTVKWEAVISYGEKVFDFDEPSEVVNHVEQAQNELAG
EGLGICEHLITLKITSSMVCDLSLIDLPGIARVAVKGQPDDIGAQIKNLILKFIKNKRTI
ILVVVPCNVDIATTEALKMAQEVDPEGTRTQAILTKPDLIDPGAEKNVLEIVHNKVVFLN
MGYVIVKCRGQKNIDENMSITDAIEEELEFFQNHEHFRSLLREEKASTKCLATKLSNALV
KHIKKSLPQMSATIKERLVEVKHLLSQIEDRPPLEPEEKRKYLIQVITDFNDQITQLSNG
DMIVEENLFELMRKEFTEWMKCLENAKSHYHEVVQQVVDEYDQKHRGSELPGFSNYRVFQ
RVVQKLVAELKRPAMMTLQTIRDMVQKQFDHLSRESFKNYPYLHRVSMRKNETIQEKQST
IVKERIVEQFEMEMQVYTQDEIFNKHSRMEGTAEGSVHDTRSKYPELLKAYYEIVVQRLA
DQVPMLISYFILKQSAKIVCSEMLDLLHRDDTDNILQEDSEIEQYRAKLQAQLDRLILAN
DKISSL
>MX6 XM_014174616.1 XP_014030091.1 ssa25 NC_027324.1:47.139.133-47.161.993
MTQQATCLPESIHSLKRKRDMSYEDGPRMFQDQLAEKVRPFIDLIDDMRSIGIDKELPLP
TIAVVGDQSSGKSSVLETLSGVALPRGTGIVTRCPLLLQLCKDRTVKWEAVISYRDKVNE
FDEPSEVVRYVEQAQSALAGKGVGICEDLITLKITSSMVCDLSLIDLPGITRVAVKGQPD
DIGAQIKNLISKFIKNKRTIILVVVPCNVDIATTEALKMAQEVDPEGTRTLAILTKPDLI
DQGAEKNVLEIVHNKVIFLNMGYVIVKCRGQKQIDENMSITCAIEEELEFFRNHEHFRSL
LREEKATTKCLATKLSNALVNEIKKYLPKMSENIKEQMGEVKHLLSQIESGPPLQPAEKR
KYLIQVITDFNDQITQLSKGDIIVVEHLFELMRKEFTEWMECLKNAKSNYHKVVQQVVDE
YDQKHRGSELPGFTNYRVFQHVVQKLVAELKNPAMMTLQKIRDMVQKQFNHLSRESFKNY
PYLHQVSMKNIETIQEQQSTIVKERIVEQFEMEMQVYTQDEIFNKHILEEGKTAGGKEHD
TRSKYPGLLKAYYEIVVQRLADQVPMLISYFMLKESAKIVCSEMLDLLSRDDTDNILQED
SEIEQKRAKLKAQVDRLILANDKISSV
>MX7 XM_014174617.1 XP_014030092.1 ssa25 NC_027324.1:47.175.786-47.193.273

MFQDQLAEKVRPFIDLIDDMRSIGIDKELPLPTIAVVGDQSSGKSSVLETLSGVSLPRGT
GIVTRCPLLLQLCNDRTVKWEAVISYRDKVNEFDEPSEVVRHVEQAQNALAGKGVGICED
LITLKITSSTVCDLSLIDLPGITRVAVKGQPDDIGAQIKNLISKFIKNKRTIILVVVPCN
VDIATTEVLKMAQEVDPEGTRTLAILTKPDLIDPGAEKNVLEIVHNKVVFLNMGYVIVKC
RGQKQIDENMSITGAIEEELEFFRNHEHFRSLLREEKATTKCLATKLSNALVNQIKKYLP
KISEKIKEKLGHVKHSLSQIESGPPLEPAEKRKYLIQVITEFNEQITQLSKGDIIVEENL
FELMGKEFAEWMKCLENAKSNYHEVVQQVVDEYDQKHRGSELPGFSNYRVFQHVVQKLVA
ELKRPAMMTLQKIKDMVQKQFYILSRERFKNHPFLHQVSKKNIETIQEKQSIIVKERIVE
QFQMEMQVYTQDEIFNKHILEEGETAADPSDCSDEDTRSKYPGLLKAYYEIVVQRLADQV
PMLIRYFILKQSAKIVCSEMLDLLHRDDTDDILQEDSEIEQYRAKLQAQADRLILANDKI
SSL
>MX8 XM_014174615.1 XP_014030090.1 ssa25 NC_027324.1:47.217.828-47.228.438
MSYEDGPRMFQDQLAEKVRPFIDLIDYMRSIGIDKELPLPTIAVVGDQSSGKSSVLETLS
GVALPRGTGIVTRCPLLLQLCNDRTVKWEAVISYGGKFRNEFDEPSEVVRHVEQAQNTLA
GKGVGICEDLITLKITSSTVCDLSLIDLPGITRVAVKGQPDDIGAQIKNLISKFIKNKRT
IILVVVPCNVDIATTEALKMAQEMDPESTRTLAILTKPDLIDPGAEKNVLEIVHNKVIFL
NMGYVIVKCRGQKQIDENMSITRAIEEELEFFQNHEHFRSLLREEKASTKCLANKLSNAL
VNHIKKSLPQMSATIKERLVEVKHLLSKLEGGPPLEPEEKRKYLIQVITDFNDQITQLSK
GDIIVEENLFVLMRKEFAEWMKCLQNDKSNYHKVVQQVVDEYDQKHRGSELPGFSNYRVF
QRVVQKLVAELKRPAMTTLQIIRDMVQKQFDHLSKECFKNYPYLHQVSMRNIETIQEQQS
NIVKERIVEQFEMEMQVYTQDEIFNKIILEEGEIAEDKDKDTRGKYPGLLKAYYEIVVQR
LADQVPMMICYFILKQSAKIVCSEMLDLLHRDDTDNILQEDSEIGQYRAKLLAQADRLIL
ANDKISSV
>MX9 XM_014174618.1 XP_014030093.1 ssa25 NC_027324.1:47.243.603-47.262.617
MSHDDGPRMFQDQLAEKVRPFIDLVDDMRSIGIDKELPLPTIVVVGDQSSGKSSVLETLS
GVALPRGTGIVTRCPLLLQLCNDRTVKWEAVISYGGKFNEFDDPSEVVRYVEQAQNALAG
KGVGICEDLITLKITSSTVCDLSLIDLPGITRVAVKGQPDDIGAQINHLIRKFIKEKRTI
ILVVVPCNVDIATTEALKMAQEVDPEGTRTLAILTKPDLIDPGAEKNVLEIVHNKVIILN
MGYVIVKCRGQKQIDENMSITHAIEEELEFFRNHEHFRSLLHEEKATTKCLANKLSNALV
KHIKKSLPKMSKKIKEQLGEVKHLLSQIESGSPLEPAEKRKYLIQVITDFNDQITQLSKG
DIIVEENLFELMRKEFTEWMECLKNAKSHYHEVVQQVVDEYDQKHRGSELPGFTNYRVFQ
HVVQKLVAELKNPAMMTLQKIRDMVQKQFDNLSRESFKNYPYLHQVSKKKIETIQENQST
IVKERIVEQFQMEMQVYTQDEIFNKHIPKEGETAAGSDKDTRSKYPGLLKAYYEIVVQRL
ADQVPMLIRYFILKQSAKIVCSEMLDLLHRDDTDNILQEDSEIGQYRAKLLAQAYRLILA
NDKISSL

SF5.6 CC and CXC chemokines

>CK10a CCL20-like XM_014143429.1 XP_013998904.1 ssa15 NC_027314.1:3.252.135-3.256.106
MVSTMDSAKILFSVLFLCVCYQVTQGQMVMDCCLEVSKKEIPQRVVTGYQPQVRGQGCSI
DAVVFHTRKGHKLCAPTGPAWVTNLIKHMDKPTKMCHNTNFKGKRCRKVKPKHS
>CK10b CCL19-like XM_014172597.1 XP_014028072.1 Ssa24 NC_027323.1:41.009.563-41.020.152
MMSAVGIAKILFCVLFLYSCCQVTQGQMLMDCCLTVSQREIPRHVVVGYQSQVRGQGCSI
DAVVFTTRRGLKLCAPADPTWVTDLMNLMDRLIKKCHETNFKAKHCKKLKNNSP
>CK13a CCL19-like XM_014128861.1 Ssa11 NC_027310.1:71.597.209-71.598.608
MSLQLAALLLLTSVLWSHAAANTDEAMDCCLTTTDAKLPRRVVKSFSIQTVSGGCRIHAT
VFVTRKNLRLCAPPAKNNNWVTKLIKQLKRKSQNGKARKGKNGKNSRH
>CK13b XM_014133457.1 ssa01 NC_027300.1:122.209.795-122.211.056
MSLQVAAFLLLASVLWSHVAASTDQAMDCCLTTTDTKLPHRVVKSYSIQTVSGGCRIAAT
VFVTKKNLRLCAPPATKNNWVAKLIKQLKRKSHKGKARKGKNGKKRH
>CXCL11_L1.1 XM_014143455.1 XP_013998930.1 ssa15 NC_027314.1:3.502.962-3.504.253
MTNMMTSTVLISFLACLLLVNVEGQVGHSKARCLCLNGMVNHVKPVLIEKLEVYTSSHSC
RNMEIIVTLKNGKGKKCLNPEAPFAKKTIEKILKNQRSVQ
>CXC11_L1.2 NM_001141223.1 NP_001134695.1 ssa15 NC_027314.1:3.514.954-3.517.032
MTNMMTSTVLISFLACLLLVNVEGQVGHSKTRCLCLNGMVNHVKPVLIEKLEVYTSKHSC
QNMEIIVTLKNGKGMKCLNPEAPFAKKTIEKIMKNQRSVQ
>CXC11_L1.3 XM_014143446.1 XP_013998921.1 ssa15 NC_027314.1:3.545.565-3.547.816
MTNMITSTVLISFLACLLLVNVEGQVGHSKARCLCLNGMVNHVKPVLIEKLEVYTSSHSC
RNMEIIVTLKNGEGKRCLNPEAPFAKKTIEKIMKKQKECAVNL

SF5.7 Deduced SOCS1 amino acid sequences sequences

```
>SOCS1.1 XM_014190655.1 XP_014046130.1 NW_012416168.1:784-1.683
MVAHSTVEEQDTTSAPSSSSSSASSSSSSVSQSHRQHPSKQCVASPIPVPDLLDQQPPLP
LLDPVPTHFPPFRCQVDFLIITRTASMLERSGFYWGPLGVEEAHSRLKDVATGTFLIRDS
RQKDVFFTLSYRADGGPVSVRIIYKGQRFSLAGSEHSFPCLFLLLEHYINSSKKSLTVPY
RKQRPTLQELCRKQVVESCSGDVERVARVPVNPVLKHFLLEFPYSI
>SOCS1.2 XM_014187410.1 XP_014042885.1 NW_012358293.1:6.016-7.112
MVAHSTVEEQDTTSAPSSSSSSASSSSSSVSQSHRQHPSKQCVASPIPVPDLLDQQPPLP
LLDPVPTHFPPFRCQVDFLIITRTASMLERSGFYWGPLGVEEAHSRLKDVATGTFLIRDS
RQKDVFFTLSYRADGGPVSVRIIYKGQRFSLAGSEHSFPCLFLLLEHYINSSKKSLTVPY
RKQRPTLQELCRKQVVESCSGDVERVARVPVNPVLKHFLLEFPYSI
>SOCS1.3 XM_014183194.1 XP_014038669.1 NW_012347411.1:23.097-27.026
MVAHSTVEEQDTTSAPSSSSSSASSSSSSVSQSHRQHPSKQCVASPIPVPDLLDQQPPLP
LLDPVPTHFPPFRCQVDFLIITRTASMLERSGFYWGPLGVEEAHSRLKDVATGTFLIRDS
RQKDVFFTLSYRADGGPVSVRIIYKGQRFSLAGSEHSFPCLFLLLEHYINSSKKSLTVPY
RKQRPTLQELCRKQVVESCSGDVERVARVPVNPVLKHFLLEFPYRI
>SOCS1.4 XM_014190859.1 XP_014046334.1 NW_012426344.1:13-1.027
IPLPCLRLPLHLPDPIPDYWTTHLRPFFSQQEYVVVRHTHRQLVHSGYYWGPMEMEEAHR
TLLHTPPGSFLIRDSSQPDVFFTLSYHGDQGPISVRVLLTSQRFRLNGSNKAFDSLFALL
RFYMESSHRRLRRAWRRERPMTLQQLCRGRIIELYGAERINSLPELNQVVTQYLQDYPYS
I
>SOCS1.5 XM_014184557.1 XP_014040032.1 NW_012349686.1:7.664-9.765 corrected
SSFRTARPSIPLPWPSPRPCLLPIPLPCLRPPLHLPDPIPDYWTTHLRPFFSQQEYVLVR
HTHRQLVHSGYYWGPMEMEEAHRTLLHTPPGSFLIRDSSQPDVFFTLSYHGDQGPISVRV
LLTSQRFRLNGSNKAFDSLFALLQFYMESSHRRLRRAWRRERPMTLQQLCRGRIIELYGA
ERIDSLPELNQVVTQYLQDYPYSI
```

SF5.8 Deduced IFI44 amino acid sequences

```
>IFI44.1 NM_001140400.1 NP_001133872.1 ssa14 NC_027313.1:62.942.467-62.979.179
MSVVTSSLSGDQEKKLLSLFGHVRLSLLYKASVQGYTAAAFHARCNSQGPTVVAAYNKAG
FLFGAYTSKDYTQNGQPINDDKAFLYSINDERKHPLRVSSANGQYGFTDENTGPNFGALV
FLFNDTATVQYNAGNSYNFDAAHMHGDDLQLTECEVYRVEDLGGCLTKPWRNMQWTSERK
RQLMEKIKKYKPDVKSVSQARVLLVGPVGAGKSSFFNSVNSIFRGNMTSQAICGSAGTSL
TTQFRTFTIKSGKHGEPIPLILCDTMGLEEASGAGLDMEDLVSIYKGHVQDRYQFNPMTP
LSEDVPGYRKHVSLKDQIHCVVYVVDACRVSLLSAKIVEKFAAIRKKTNQIGIPQLVIMT
KVDEACPLVSEELQNVYMSHYIQRKIQELSASLGIPVSGILPVKNYSHELELNQNTDLLL
LSALDQMLNYADSFFENQPEEEKEEL
>IFI44.2 NM_001140400.1 NP_001133872.1 ssa14 NC_027313.1:63.251.718.-63.254.829
MSVVTSSLSGDQEKKLLSLFGHVRLSLLYKASVQGYTAAAFHARCNSQGPTVVAAYNKAG
FLFGAYTSKDYTQNGQPINDDKAFLYSINDERKHPLRVSSANGQYGFTDENTGPNFGALV
FLFNDTATVQYNAGNSYNFDAAHMHGDDLQLTECEVYRVEDLGGCLTKPWRNMQWTSERK
RQLMEKIKKYKPDVKSVSQARVLLVGPVGAGKSSFFNSVNSIFRGNMTSQAICGSAGTSL
TTQFRTFTIKSGKHGEPIPLILCDTMGLEEASGAGLDMEDLVSIYKGHVQDRYQFNPMTP
LSEDVPGYRKHVSLKDQIHCVVYVVDACRVSLLSAKIVEKFAAIRKKTNQIGIPQLVIMT
KVDEACPLVSEELQNVYMSHYIQRKIQELSASLGIPVSGILPVKNYSHELELNQNTDLLL
LSALDQMLNYADSFFENQPEEEKEEL
>IFI44.3 XM_014151635.1 XP_014007110.1 ssa17 NC_027316.1:9.105.5499.110.942
MVALRDLDFVVDYVVIEAQQPTALKELYFHDHQLQTFLEDVMSVVKSSLSVEQERKLLSL
FGHVRLHLLYKASVHGYMSLAFHSRCDGQGPTVLVAYNRAGFVFGGYISKDYAQTGQAIN
DDKAFLYSITDQREKPLRVSSTDGQYGFTDGAYGLNVGALWFLNNNTATVQVVAGNCYAF
EGEEMHGNDLQLTECEVYRVEGCEGILETPWRKIDLEGYGTKDRLMDYIKNYKPEVKSVV
QARVLLVGPVGAGKSSFFNSINSVFKGHVTGQANTGSAGTSLTTQFRTYNIKAEQGGKAL
PLVLCDTMGLEEGPSAGLDIDDITSILKGHVQDRYQFNPSMSVQTDGVGFCKSPSLKDMI
HCVVYVLDACKVTLLSAKMVDKLAAIRKGINKMGIPQLVLLTKVDEACPLVREDLTNIYL
SHYIERMTREVSVCLGVSQSCVLPVKNYSREFELDIHTDILLLTAVVQMLRYTDDYFDDI
YQAGDQKSE
>IFI44.4 XM_014149377.1 XP_014004849.1 ssa16 NC_027315.1:43.549.791-43.558.484
MSFNFGMVSGGCPNFSAPVLHGEKPSSKPLATDLLRGDGRPDSSLPWAEKCKSKMESPWR
DVAWTEDRRETLMESISSYKPGSEALSEARVLLVGPVGAGKSSFISSVQSVFTGRFTNRA
```

MVGSSSASFTKKVELQLFNIRGRSPEQPTALVLCDVMGLGDGETTGLTLHDTLAIIKGHA
PEGHKFSPEQPVRSETVGYVKKPSLKDKVHCVVFVVDASKVSSYTKGLGTTFQQLREHIS
DLGVHQVALLTHVDKVCQETARDITQVYNSRIVQQTMTKAGALLGMSTSYIVPVKNYSSE
LDVDENTDILLLSAVDHILQYVDLYFQDCAATYPKLSL
>IFI44.5 XM_014149379.1 XP_014004854.1 ssa16 NC_027315.1:43.616.73043.627.930
MMKSWLRIKRLNDETAQRTTPTETNVIPTMNHKLTQEQEKQLCSLLGNVKLSLLFKASIY
GYTGAAFHQKCDHQGPTVSVGYNSTGFVFGGYTSKDHDVAKQGQYIQDDKAFLFSLTGRN
PVRYPVTNAQYAVKMHNTTGPYFGEALLLMNANTATVISIPGKYYNFNAAEMHGNNLNLT
ECEVYKVEEGGNIIEKPWRTILWKAERRKELMDSVKLYRPMISSVGQARVLLIGPVGAGK
SSFFNSVNSIFRGHVTSQAISGSSGTSLTTQFRTYSVKAGRDGKPLAFILCDTMGMEEAT
GAGLDVDDISSILKGHVPDRYQFNPSVPLQADAHGFRQSVKLHERIHCVVYVMDTCKVSI
MSTKLEEKLVAIRRRVNLLGIPQLVLLTKVDEACPCVANNLRNVYNSQYIKTKAQEVSGR
LGLPMSCIVPVKNYSEELELDMSCDILLLSALIQMLRFADNYFDDVNDQEKHD
>IFI44.6 XM_014149381.1 XP_014004856.1 ssa16 NC_027315.1:43.668.157-43.675.309
MNPKLTQEQEKQLCSLLGNVKLSLLFKASIYGYTGAAFHQKCDHQGPTVSVGYNSTGFVF
GGYTSKDHDVAKQGQYIQDDKAFLFSLTGRNPVRYPVTNAQYAVKMHNTTGPYFGEALLL
MNANTATVISIPGNYYNFNAAEMHGNNLNLTECEVYKVEEGGNIIEKPWRTILWKAERRK
ELMDSVKLYRPMISSVGQARVLLIGPVGAGKSSFFNSVNSIFRGHVTSQAISGSSGTSLT
TQFRTYSVKAGRDGKPLPIILCDTMGMEEATGAGLDVDDISSILKGHVPDRYQFNPSVPL
QADAHGFRQSVKLHERIHCVVYVMDTCKVSIMSTKLEEKLVAIRRRVNLLGIPQLVLLTK
VDEACPCVANNLRNVYNSQYIKTKAQEVSGRLGLPMSCIVPVKNYSEELELDMSCDILLL
SALIQMLRFADNYFDDVNDQEKHD
>IFI44.L1a XM_014132022.1 XP_013987497.1 ssa12 NC_027311.1:44.185.618-44.193.898
MGGEESKPSPPPPPTPTPPPPPPPPPKEFDKEWRETSWSKGERDRMVDELRSFKLSDPDVG
QLRCLLYGPVGAGKSSFINSVNNVFQNRTTSGALVASTSGTSFTMTYDTHYIQGRSGEKR
LPFAFNDVMGLETQKNGLHPDDIINALKGHLPEGYEFNPFHPLSDQKKEFIQNPSLSDKT
HCLVSIVSADKISRMQKDVIDKMKNVRAEASRLKIPQVLVMTMPDMACDVVNKDVKRIFY
SKAIKEKMQICSNELGLPMNCILPVKNYHEEGMLDNDMDILILNAMTQIMNFANDYLWNL
QQHAIQK
>IFI44.L1b1 XM_014167585.1 XP_014023062 ssa22 NC_027321.1:42.904.84442.926.820
MGGGGSTSPAPPPPPPPKKEFDQQWRKTCWSKKQRDAMVDALRKFELSDPDVGQLRCLLH
GPVGAGKSSFINSVNNVFQGRSTHNALVAAASGTSFTMKYKTHYFQGRKGENRLPFAFND
VMGLETQKNGVHPDDIINALKGHLPEGYEFNPLHPLSDQKEEYIQNPSLSDKTHCLVSIV
TADTISRMNKDVIVKMKHIRAEASKLQIPQVVVMTMPDKACELVNKDVKRIYFSKAIKDK
MQICSNELGVPMNCILPVKNYHEEGMLDNDMDILILNAFTQIMNFANDYIGDLQQHA
>IFI44.L1b2 XM_014167590.1 XP_014023067 ssa22 NC_027321.1:42.975.136-42.984.875
MVAALRNFKLSDPDMGQLRCLLYGPVGAGKSSFVNSVNNVFQGRVAHNALVAAASGTSFT
KTYNTHYIKDGDKRLPFAFNDVMGLEAQEKGMQPEDIINALKGHLPEGYKFNPCSALTDE
SAEYNKKPRSSDKVHCLVSVVAADKISLMSNDVIVKMRKVREKASELGIAQVVVMTMPDK
ACPLVEMDVKKMYTSKAIKDKMQICSNEVGIPMNCILPVKNYHEEGMLDNDMDILILNAM
TQMVNFANDYIWNLQQTA
>IFI44.L1b3 XM_014167588.1 XP_014023063 ssa22 NC_027321.1:43.039.358-43.066.852
MYHMFKDDWRFPTLTPTPTPPPPPPPKKEFDSPWRKVSWSKQHRDEMVRVLRNFELNDPD
VGQLRCLLHGPVGAGKSSFINSVNNVFQGRTTSGALVAATSGTSFTKTYKTHYIKDGNSR
LPFAFNDVMGLEAQEKGMQTKDIINALKDHLPEGYKFNPCWPLTEESPEYNRIPTSSHKV
HCLVSVVPADKISLMSSDVTAKMRKVREKASKMGIPQVVVMTMPDQACDLVDRDVKNIYT
SKAIKEKMQICSNELGFPMNCILPVKNYHEEEMLDDDMDILILNAMTQIMNFANDYIWDL
QQHA
>IFI44.L1b4 XM_014203108.1 XP_014058583 ssa06 NC_027305.1:19.840.801-19.856.156
MQLFKSTTPPMPTAAFDKEWRITPWGQKEVILQKLKDYVPGNPDIKSIRVFLHGPVGAGK
SSLINFINSVFQGRITSIALADSAIAAESFTIKYQTHKIEKGKHGIYYPIVFNDVMGLEE
SSGKGVHKDDIINALKGHVKEGHKFNPMSPLSEEDPGYIKIPSSEDRVHCLVSVMPADKM
AFLGKHVIQKMRDIRLAADMGIPQMVVLTRVDEACPSVRKDVKNIYLSKYIKEKMEQCS
TELGVPVNCILPVKNYHEEIDLNEDMDVLLLRALRQMVDFADDFIKNIPLKITPQGN
>IFI44.L2 XM_014167582.1 XP_014023057 ssa22 NC_027321.1:43.104.110-43.110.856
MGGKESMPPKTEFDSPWRDQNWDKQERDTMVAALRNFKLSDPDMGQLRCLLYGPVGAGKS
SFVNSVNNVFQGRVAHNALVAAASGTSFTKTYNTRYIKDGDKRLPFAFNDVMGLEAQEKG
MQAEDIINALKGHLPEGYKFNPCSALTDESGEYNKKPRSSDKVHCLVSVVAADKISLMSN
DVIVKMRKVREKASELGIPQVDVMTMPDKACPLVKMDVKKMYTSKAIKDKMLICSNELGV
PMNCILPVKNYHEEGMLDNDMDILILNAMTQIMNFANDYIWNLQQGKENGGCHVCCI

SF3.9. Beta2-microglobulin protein and promoter sequences

```
>b2m1 NM_001123699.1 NP_001117171.1 NC_027306.1:57.825.509-57.828.543
MKSILSIVVLVLIYSAVESKESPPKVQVYSRNPGNFGDKNTLICHVSGFHPPDISIQLLK
NGVEIPDAKQTDLAFEQGWQFHLTKSVGFTPDSGEEYTCRVRHLKNLKTYTWEADM
>b2m2 XM_014208868.1 XP_014064343.1 NC_027306.1:58.301.914-58.303.127
MKSILSIVVLVLIYSAVESKESPPKVQVYSRNPGNFGDKNTLICHVSGFHPPDISIQLLK
NGVEIPDAKQTDLAFEQGWQFHLTKSVGFTPDSGEEYTCRVRHLKNLKTYTW
>b2m3 XM_014188686.1 XP_014044161.1 NW_012366138.1:15-1.215
MKSILSIVVLVLIYSAVESKESPPKVQVYSRNPGNFGDKNTLICHVSGFHPPDISIQLLK
NGVEIPDAKQTDLAFEQGWQFHLTKSVGFTPDSGEEYTCRVRHLKNLKTYTW
>b2m4 XM_014187757.1 XP_014043232.1 NW_012360375.1:3.156-4.398
MKSILSIVVLVLIYSAVESKESPPKVQVYSRNPGNFGDKNTLICHVSGFHPPDISIQLLK
NGVEIPDAKQTDLAFEQGWQFHLTKSVGFTPDSGEEYTCRVRHLKNLKTYTWEPDM
>b2m5 XM_014189380.1 XP_014044855.1 NW_012376632.1:2.522-3.764
MKSILSIVVLVLIYSAVESKESPPKVQVYSRNPGNFGDKNTLICHVSGFHPPDISIQLLK
NGVEIPDAKQTDLAFEQGWQFHLTKSVGFTPDSGEEYTCRVRHLKNLKTYTWEPDM
>b2m6 XM_014184298.1 XP_014039773.1 NW_012349147.1:6.171-7.264
MKTVLSVIAFCVFLGFINAKESPPKVQVYSRNPGNFGDKNTLICHVSGFHPPDISIQLLK
NGVEIPDAKQTDLAFEQGWQFHLTKSVGFTPARGEEYTCRVRHLKNLKTYTWESNM
>b2m7 XM_014184299.1 XP_014039774.1 NW_012349147.1:9.889-11.950
MKTVLSVIAFCVFLGFINAKESPPKVQVYSRNPGNFGDKNTLICHVSGFHPPDISIQLLK
NGVEIPDAKQTDLAFEQGWQFHLTKSVGFTPDSGEEYTCRVRHLKNLKTYTWEADM
>b2m8 XM_014153920.1 XP_014009395.1 NC_027316.1:57.628.552-57.632.032
MKTVLSVIAFCVFLGFINAKESPPKVQVYSRNPGEHGKDNTLICHVSGFHPPDISIQLLK
NGVEIPDAKQTDLAFEQGWQFHLTKSVGFTPDSGEEYTCRVRHLKNLRSTPGNQICKASV
LIAAEYTSLRESPLQMLWIFLGIIFTTLSYQN
>b2m9 NM_001245913.1 NP_001232842.1 NW_012347820.1:16.383-20.477
MKTVLSVIAFCVFLGFINAKESPPKVQVYSRNPGEHGKDNTLICHVSGFHPPDISIQLLK
NGVEIPDAKQTDLAFEQGWQFHLTKSVGFTPDSGEEYTCRVRHLKNLKTYTWESNM
>b2m10 NM_001198574.1 NP_001185503.1 NW_012353146.1:474-1.303
MKTVLSVVAFCVVLVTINAKESPPKVQVYSRNPGEHGKDNTLICHVSGFHPPDISIQLLK
NGVEIPDAKQTDLAFEQGWQFHLTKSVGFTPDSGEEYTCRVRHLKNLKTYTWEPDM
>b2m11 XM_014190178.1 XP_014045653.1 NW_012394841.1:962-1.728
MKTVLSVVAFCVVLVTINAKESPPKVQVYSRNPGEHGKDNTLICHVSGFHPPDISIQLLK
NGVEIPDAKQTDLAFEQGWQFHLTKSVGFTPDSGEEYTCRVRHLKNLKTYTW
>b2m12 XM_014190151.1 XP_014045626.1 NW_012394112.1:459-1.277
MKTVLSVVAFCVVLVTINAKESPPKVQVYSRNPGEHGKDNTLICHVSGFHPPDISIQLLK
NGVEIPDAKQTDLAFEQGWQFHLTKSVGFTPDSGEEYTCRVRHLKNLKTYTWGELLV
```

B2m promoter sequences terminating at Start codon ATG:

B2m9 and b2m10 had open reading frame errors making definition of promoter region difficult and are thus not presented.

```
>b2m1 NC_027306.1:57.829.543-57.825.509
GACGTGCGTCAGTTCACTCCACTTCCGGCTTCCGGCTTGGTTTGTCATGTGACATGTCTC
GTGATTTCTGTGAAACCTGCAATAGCACAACACACCACCTACTGTTGACAAAGGGTCATT
TTCAGGGAACTATTAAAGCGGTAGAGTAGACTATGCTCAACTTTATGCTAAAACTCAAAA
CAAAAACCTTAATTAAAAGAGGTATGTGTATTGTGGCCTAGGGTGGTCTAGTGGTCTATG
CCGCACGTCTACGGTGTTGGCCTGGGTTCAAATCCGGCGTACTGCCTGATATAAACTATC
TTTCCCCACTGTCATCCTCACTCTGTTCAATAAAATCTGAAAATACCGTATAAATACACA
TATATTTTTAAAGAGATACATGCATTGTTATATTGGATTAAGTAATGGTGTGTAACCATT
CTAGCAACTTTTCACTGTAATGACTAATATATTTGGGCTCCCGAGTGGCGCAGCAGTCTA
AAGGTACTGCATCTCAGTGCAAGAGTCCCTGGTTCGAATTAAGGCTGTATCACATCCCAT
AGGGCGGCGCACAATTGGTCCCAGCGTCGTCCGGGTTTGGCCGGGGTAGGCCGTCATTGT
TAATAAGAATTTGTTCTTAACTGACTTGCCTAGTTAAATAAAGTTTCAATAAATACAAAT
TACAATTAGATCAACAGAGACACAAAATATTGAAAAGGCAAAACAACTATTCTATTCTAT
ATTTTTTGATTCTCAAAACGGATGCCTAGGTGAAATAACATTATCTCATGACATACATGT
TAAATCGAAATTGTTATTATGGTAGCTCGCCTGGATTTCAGGTTATTTCCAGACTATAGT
CCTCCCCAATAAACTACCTAACCTGTGAGGCTTTCTTTAGCCTAACAACAGAAATATACC
ACTTTTGATTGGCTTTTAAAATAGAGAGGAGGCGGTCTGGAGAGTATTTATACGACTATC
TGCCTTACTTTCAGTTAGGTTATTTCCACAGACAGACACAGACAGACCGATCTGTCCATT
```

ATCTAGTTTAGTTGACTACGAATAATTCCGAATTAGACCAAAT**ATG**
>b2m2 NC_027306.1:58.300.914-58.303.127
CTTTAATTCAGACTAAAATACTGTGTGATGTTTTCTGTTTTGGGCCCTTGCACAGCTGTT
CCTGTTGACGTGCGTCAGTTCACTCCACTTCCGGCTTCCGGCTTGGTTTGTCATGTGACA
TGTCTCGTGATTTCTGTGAAACCTGCAATAGCACAACACACCACCTACTGTTGACAAAGG
GTCATTTTCAGGGAACTATTAAAGCGGTAGAGTAGACTATGCTCAACTTTATGCTAAAAC
TCAAAACAAAAACCTTAATTAAAAGAGGTATGTGTATTGTGGCCTAGGGTGGTCTAGTGG
TCTATGCCGCACGTCTACGGTGTTGGCCTGGGTTCAAATCCGGCGTACTGCCTGATATAA
ACTATCTTTCCCCACTGTCATCCTCACTCTGTTCAATAAAATCTGAAAATACCGTATAAA
TACACATATATTTTTAAAGAGATACATGCATTGTTATATTGGATTAAGTAATGGTGTGTA
ACCATTCTAGCAACTTTTCACTGTAATGACTAATATATTTGGGCTCCCGAGTGGCGCAGC
AGTCTAAAGGTACTGCATCTCAGTGCAAGAGTCCCTGGTTCGAATTAAGGCTGTATCACA
TCCCATAGGGCGGCGCACAATTGGTCCCAGCGTCGTCCGGGTTTGGCCGGGGTAGGCCGT
CATTGTAAATAAGAATTTGTTCTTAACTGACTTGCCTAGTTAAATAAAGTTTCAATAAAT
ACAAATTACAATTAGATCAACAGAGACACAAAATATTGAAAAGGCAAAACAACTATTCTA
TTCTATATTTTTTGATTCTCAAAACGGATGCCTAGGTGAAATAACATTATCTCATGACAT
ACATGTTAAATCGAAATTGTTATTATGGTAGCTCGCCTGGATTTCAGGTTATTTCCAGAC
TATAGTCCTCCCCAATAAACTACCTAACCTGTGAGGCTTTCTTTAGCCTAACAACAGAAA
TATACCACTTTTGATTGGCTTTTAAAATAGAGAGGAGGCGGTCTGGAGAGTATTTATACG
ACTATCTGCCTTACTTTCAGTTAGGTTATTTCCACAGACAGACACAGACAGACCGATCTG
TCCATTATCTAGTTTAGTTGACTACGAATAATTCCGAATTAGACCAAAT**ATG**
>b2m3 NW_012366138.1:1-2.200
TGTTTTCTGTTTTGGGCCCTTGCACAGCTGTTCCTGTTGACGTGCGTCAGTTCACTCCAC
TTCCGGCTTCCGGCTTGGTTTGTCATGTGACATGTCTCGTGATTTCTGTGAAACCTGCAA
TAGCACAACACACCACCTACTGTTGACAAAGGGTCATTTTCAGGGAACTATTAAAGCGGT
AGAGTAGACTATGCTCAACTTTATGCTAAAACTCAAAACAAAAACCTTAATTAAAAGAGG
TATGTGTATTGTGGCCTAGGGTGGTCTAGTGGTCTATGCCGCACGTCTACGGTGTTGGCC
TGGGTTCAAATCCGGCGTACTGCCTGATATAAACTATCTTTCCCCACTGTCATCCTCACT
CTGTTCAATAAAATCTGAAAATACCGTATAAATACACATATATTTTTAAAGAGATACATG
CATTGTTATATTGGATTAAGTAATGGTGTGTAACCATTCTAGCAACTTTTCACTGTAATG
ACTAATATATTTGGGCTCCCGAGTGGCGCAGCAGTCTAAAGGTACTGCATCTCAGTGCAA
GAGTCCCTGGTTCGAATTAAGGCTGTATCACATCCCATAGGGCGGCGCACAATTGGTCCC
AGCGTCGTCCGGGTTTGGCCGGGGTAGGCCGTCATTGTAAATAAGAATTTGTTCTTAACT
GACTTGCCTAGTTAAATAAAGTTTCAATAAATACAAATTACAATTAGATCAACAGAGACA
CAAAATATTGAAAAGGCAAAACAACTATTCTATTCTATATTTTTTGATTCTCAAAACGGA
TGCCTAGGTGAAATAACATTATCTCATGACATACATGTTAAATCGAAATTGTTATTATGG
TAGCTCGCCTGGATTTCAGGTTATTTCCAGACTATAGTCCTCCCCAATAAACTACCTAAC
CTGTGAGGCTTTCTTTAGCCTAACAACAGAAATATACCACTTTTGATTGGCTTTTAAAAT
AGAGAGGAGGCGGTCTGGAGAGTATTTATACGACTATCTGCCTTACTTTCAGTTAGGTTA
TTTCCACAGACAGACACAGACAGACCGATCTGTCCATTATCTAGTTTAGTTGACTACGAA
TAATTCCGAATTAGACCAAAT**ATG**
>b2m4 NW_012360375.1:3.100-5.300
GTGCGTCAGTTCACTCCACTTCCGGCTTCCGGCTTGGTTTGTCATGTGACATGTCTCGTG
ATTTCTGTGAAACCTGCAATAGCACAACACACCACCTACTGTTGACAAAGGGTCATTTTC
AGGGAACTATTAAAGCGGTAGAGTAGACTATGCTCAACTTTATGCTAAAACTCAAAACAA
AAACCTTAATTAAAAGAGGTATGTGTATTGTGGCCTAGGGTGGTCTAGTGGTCTATGCCG
CACGTCTACGGTGTTGGCCTGGGTTCAAATCCGGCGTACTGCCTGATATAAACTATCTTT
CCCCACTGTCATCCTCACTCTGTTCAATAAAATCTGAAAATACCGTATAAATACACATAT
ATTTTTAAAGAGATACATGCATTGTTATATTGGATTAAGTAATGGTGTGTAACCATTCTA
GCAACTTTTCACTGTAATGACTAATATATTTGGGCTCCCGAGTGGCGCAGCAGTCTAAAG
GTACTGCATCTCAGTGCAAGAGTCCCTGGTTCGAATTAAGGCTGTATCACATCCCATAGG
GCGGCGCACAATTGGTCCCAGCGTCGTCCGGGTTTGGCCGGGGTAGGCCGTCATTGTAAA
TAAGAATTTGTTCTTAACTGACTTGCCTAGTTAAATAAAGTTTCAATAAATACAAATTAC
AATTAGATCAACAGAGACACAAAATATTGAAAAGGCAAAACAACTATTCTATTCTATATT
TTTTGATTCTCAAAACGGATGCCTAGGTGAAATAACATTATCTCATGACATACATGTTAA
ATCGAAATTGTTATTATGGTAGCTCGCCTGGATTTCAGGTTATTTCCAGACTATAGTCCT
CCCCAATAAACTACCTAACCTGTGAGGCTTTCTTTAGCCTAACAACAGAAATATACCACT
TTTGATTGGCTTTTAAAATAGAGAGGAGGCGGTCTGGAGAGTATTTATACGACTATCTGC
CTTACTTTCAGTTAGGTTATTTCCACAGACAGACACAGACAGACCGATCTGTCCATTATC
TAGTTTAGTTGACTACGAATAATTCCGAATTAGACCAAAT**ATG**
>b2m5 NW_012376632.1:1.750-3.788
TAAAGCGGTAGAGTAGACTATGCTCAACTTTATGCTAAAACTCAAAACAAAAACCTTAAT
TAAAAGAGGTATGTGTATTGTGGCCTAGGGTGGTCTAGTGGTCTATGCCGCACGTCTACG
GTGTTGGCCTGGGTTCAAATCCGGCGTACTGCCTGATATAAACTATCTTTCCCCACTGTC

ATCCTCACTCTGTTCAATAAAATCTGAAAATACCGTATAAATACACATATATTTTTAAAG
AGATACATGCATTGTTATATTGGATTAAGTAATGGTGTGTAACCATTCTAGCAACTTTTC
ACTGTAATGACTAATATATTTGGGCTCCCGAGTGGCGCAGCAGTCTAAAGGTACTGCATC
TCAGTGCAAGAGTCCCTGGTTCGAATTAAGGCTGTATCACATCCCATAGGGCGGCGCACA
ATTGGTCCCAGCGTCGTCCGGGTTTGGCCGGGGTAGGCCGTCATTGTAAATAAGAATTTG
TTCTTAACTGACTTGCCTAGTTAAATAAAGTTTCAATAAATACAAATTACAATTAGATCA
ACAGAGACACAAAATATTGAAAAGGCAAAACAACTATTCTATTCTATATTTTTTGATTCT
CAAAACGGATGCCTAGGTGAAATAACATTATCTCATGACATACATGTTAAATCGAAATTG
TTATTATGGTAGCTCGCCTGGATTTCAGGTTATTTCCAGACTATAGTCCTCCCCAATAAA
CTACCTAACCTGTGAGGCTTTCTTTAGCCTAACAACAGAAATATACCACTTTTGATTGGC
TTTTAAAATAGAGAGGAGGCGGTCTGGAGAGTATTTATACGACTATCTGCCTTACTTTCA
GTTAGGTTATTTCCACAGACAGACACAGACAGACCGATCTGTCCATTATCTAGTTTAGTT
GACTACGAATAATTCCGAATTAGACCAAAT**ATG**
>b2m6 NW_012349147.1:6.100-8.140
CACATAATGCACTTTTACTTTCTTCTCCAACACTTTGTTTTTGCATTATTTAAACCAAAT
TGAACATGTTTCATTATTTATTTGAGGCTAAATTGATTTTATTGATGTATTATATTAAGT
TAAAATAAGTGTTCATTCAGTATTGTTGTAATTGTCATTATTACAAATAAATAAATAAAA
ATCGGCCCGATAAATCGGTAGCGGCTTTTTTGGTCCTCCAATAATCGGTATCGGCGTTGA
AAAATCATAATCGGCCGGCCGCTAACTCACATTGCCAAGGCAGGTAGGCAATGTATGCAG
AGCACAGTAGTCTACAGCATTATATTGGGTGATGATACGATGTTGACATTTCAGATCATA
CACTATACTTTATTACCATACCTTCTCTCAATGCAATGCATGTCCAGCATTGTAACTGGT
AAAATGAAGATTGCGTCAACTTTGTAACAGACTGTCATGGCTAGAAGGGATACATACAGC
TTTTGTCAAATTCAAGTCATTTAAAAAAAAAAAAAATTGTTCTTTTGCATTTCAGCAAACC
CTTTTCCAAACCTTATCCAAATTCGCCTAACCTGCTACAAAAAGTCAATTCTCACGTTCA
TTTCACAAAAGCTGTTTCTCTTCTAACCCTGTAACCTTTTGGGTCTCAATTTAAGGTTAG
GCACAAAGTTAACAGTGTGGTTAAGGTTGAGTTTAAATCAGATAAATTGTGGAATTGGGT
GGAAATGGGTGGGGTTTAGCCATAATTATTACTTTGTGGCTGTATTAACTAGTGACGACC
CTGGATCAGCCTAGAGAAACATCGAGAGGTGAAGCCTGCATAGCAACTGTGAAGTACGAT
TTTCAATTGGCGCATCAATCGAAGTGGGTGCGGCTTTAGTACATTTCAGGAAGAATATAT
ATTTCACTTTCATCAGACACCATCTGCAAAGTCTGTTTTGTTCTGTTTGGCGCAGTTGTA
TATTTTTTCAGCCTTGGAAAGAAATC**ATG**
>b2m7 XM_014184299.1 XP_014039774.1 NW_012349147.1:12.760-13.693
TAAAGCGGTAGAGTAGACTATGCTCAACTTTATGCTAAAACTCAAAACAAAAACCTTAAT
TAAAAGAGGTATGTGTATTGTGGCCTAGGGTGGTCTAGTGGTCTATGCCGCACGTCTACG
GTGTTGGCCTGGGTTCAAATCCGGCGTACTGCCTGATATAAACTATCTTTCCCCACTGTC
ATCCTCACTCTGTTCAATAAAATCTGAAAATACCGTATAAATACACATATATTTTTAAAG
AGATACATGCATTGTTATATTGGATTAAGTAATGGTGTGTAACCATTCTAGCAACTTTTC
ACTGTAATGACTAATATATTTGGGCTCCCGAGTGGCGCAGCAGTCTAAAGGTACTGCATC
TCAGTGCAAGAGTCCCTGGCTCGAATTAAGGCTGTATCACATCCCATAGGGCGGCGCACA
ATTGGTCCCAGCGTCGTCCGGGTTTGGCCGGGGTAGGCCGTCATTGTAAATAAGAATTTG
TTCTTAACTGACTTGCCTAGTTAAATAAAGTTTCAATAAATACAAATTACAATTAGATCA
ACAGAGACACAAAATATTGAAAAGGCAAAACAACTATTCTATTCTATATTTTTTGATTCT
CAAAACGGATGCCTAGGTGAAATAACATTATCTCATGACATACATGTTAAATCGAAATTG
TTATTATGGTAGCTCGCCTGGATTTCAGGTTATTTCCAGACTATAGTCCTCCCCAATAAA
CTACCTAACCTGTGAGGCTTTCTTTAGCCTAACAACAGAAATATACCACTTTTGATTGGC
TTTTAAAATAGAGAGGAGGCGGTCCTGGAGAGTATTTATACGACTATCTGCCTTACTTTC
AGTTAGGTTATTTCCACAGACAGACACAGACAGACCGATCTGTCCATTATCTAGTTTAGT
TGACTACGAATAATTCCGAATTAGACCAAAT**ATG**
>b2m8 nnn region 5 prime NC_027316.1:57.628.552-57.632.032
TTTATCAGCTTCTTGATATGCCACACCTATCAGGTGGATAGATTATCTTGGCAAAGGAGA
AATGCTCATCAATAGGGATGTAAACAAATGTGTGTACATCAATTGAGAGCAGTAAGCTTT
TTGTGCCCATGGATAATTTCTGGGATCTTTTATTTCAACTCATGAAACATGGGACCAACA
CTACATATTGCTTTTATATTTTTGTTCACCATACTTTATTATGGCTATGCTGTACGTGGC
CTACCTCGCCTTTCACCATACTTTATTATGGCTATGCTGTACGTGACCTACCTAGCCTTT
CACCATACTTTATTATGGCTATACTGTACCTAGCCTTTCACCATACTTTATTATGGCTAT
ACTGTACATGACCTACCTAGCCTTTCACCATACTTTATTATGGCTATACTGTACCTAGCC
TTTCACCATACTTTATTATGGCTATACTGTACGTGGCCTTTCACCATACTTTATTATGGC
TATACTGTACGTGGCCTTTCTAGCCTTTATTATGACTATGCTCTACGTGAACTAGCCTTT
CACCATATTTTATTATGACTATACTGTACATGACCTTGCCTTTCAACATACTTTATCTCA
ATGTATTATGTGTGCAACATTGTAACTAATAAAATGAAGACTGCATCAACTTTGTCACTG
ACTGCATCAGGCTGCCTAAAGAAGCATCAAGAGGAGAAGACTGCATAGCAACTGTGAACT
AGGATTTTCAATTGGTGCATAAATCGAAGAGGGTGCGGCTTTACTACATTGAGTAAAATA
TATATTTTACTTTCAGTTTCATTTCTTCGTTTGCGACTGTTGGTTTGGTCCAGTTTCATA
TATTTTTCAGCATTGGAAAGACTTGGAAAAAGAAATC**ATG**

```
>b2m11 NW_012394841.1:1-2.268
CTTTATTATGGCTATGCTGTACGTGACCTACCTAGCCTTTCACCATACTTTATTATGACT
ATACTGTACCTAGCCTTTCACCATACTTTATTATGGCTATACTGTACCTAGCCTTTCACC
ATACTTTATTATGGCTATACTGTACATGACCTACCTAGCCTTTCACCATACTTTATTATG
ACTATACTGTACCTAGCCTTTCACCATACTTTATTATGGCTATACTGTACGTGGCCTTTC
TAGCCTTTATTATGGCTATACTGTACGTGGCCTTTCTAGCCTTTATTATGACTATGCTCT
ACGTGAACTAGCCTTTCACCATATTTTATTATGACTATACTGTACATGACCTTGCCTTTC
AACATACTTTATCTCAATGTATTATGTGTGCAACATTGTAACTAATAAAATGAAGACTGC
ATCAACTTTGTCACTGACTGCATCAGGCTGCCTAAAGAAGCATCAAGAGGAGAAGACTGC
ATAGCAACTGTGAACTAGGATTTTCAATTGGTGCATAAATCGAAGAGGGTGCGGCTTTAC
TACATTGAGTAAAATATATATTTTACTTTCAGTTTCATTTCTTCGTTTGCGACTGTTGGT
TTGGTCCAGTTTCATATATTTTTCAGCATTGGAAAGACTTGGAAAAAGAAATCATG
>b2m12 NW_012394112.1:1-2.304
TTCACTCATGAAACATGGGACCAACACTACATATTGCTTTTATATTTTTGTTCACCATAC
TTTATTATGGCTATGCTGTACGTGGCCTACCTAGCCTTTAACCATACTTTATTATGGCTA
TGCTGTACGTGACCTACCTAGCCTTTCACCATACTTTATTATGACTATACTGTACCTAGC
CTTTCACCATACTTTATTATGGCTATACTGTACCTAGCCTTTCACCATACTTTATTATGG
CTATGCTGTACGTGACCTACCTAGCCTTTCACCATACTTTATTATGACTATACTGTACCT
AGCCTTTCACCATACTTTATTATGGCTATACTGTACATGACCTACCTAGCCTTTCACCAT
ACTTTATTATGGCTATACTGTACCTAGCCTTTCACCATACTTTATTATGGCTATACTGTA
CCTAGCCTTTCACCATACTTTATTATGGCTATACTGTACATGACCTACCTAGCCTTTCAC
CATACTTTATTATGGCTATACTGCCCCTAGCCTTTCACCATACTTTATTATGGCTATACT
GTACATGACCTACCTAGCCTTTCACCATACTTTATTATGGCTATACTGTACCTAGCCTTT
CACCATACTTTATTATGGCTATACTGTACATGACCTACCTAGCCTTTAACCATACTTTAT
TATGACTATACTGCCCCTAGCCTTTCACCATACTTTATTATGGCTATACTGTACCTAGCC
TTTCACCATACTTTATTATGGCTATACTGTACATGACCTACCTAGCCTTTAACCATACTT
TATTATGACGTGAACTAGCCTTTCACCATATTTTATTATGACTATACTGTACATGACCTT
GCCTTTCAACATACTTTATCTCAATGTATTATGTGTGCAACATTGTAACTAATAAAATGA
AGACTGCATCAACTTTGTCACTGACTGCATCAGGCTGCCTAAAGAAGCATCAAGAGGAGA
AGACTGCATAGCAACTGTGAACTAGGATTTTCAATTGGTGCATAAATCGAAGAGGGTGCG
GCTTTACTACATTGAGTAAAATATATATTTTACTTTCAGTTTCATTTCTTCGTTTGCGAC
TGTTGGTTTGGTCCAGTTTCATATATTTTTCAGCATTGGAAAGACTTGGAAAAAGAAATC
ATG
```

SF5.10 Peptide loading complex sequences

```
>CALR1a XP_013995146.1 ssa14 NC_027313.1:15.482.204-15.485.137
MLVSVLLMIALASAKPSVYFREQFEDDAWNTRWVESSHRSDYGKFVLTAGKFYGDAEKDK
GLQTSQDAHFYSSSARFEPFSNQGKTLVIQFTVKHEQNIDCGGGYIKLFPADLDQADMHG
DSNYNIMFGPDICGPATKKIHVIINYKGKNHLIRKDIRCKDDEYTHLYTLILNPDNTYEV
KIDNKKVESGSLEEDWDILPPKKVKDPEAVKPDHWDERERMEDPDDKKPEDWDRPENIAD
PDAKQPEDWDDEMDGEWEPPMVSNPDYKGEWKPRTIDNPDYKGKWLHPEIDNPDYSADSE
IYRFDSIGVIGLDLWQVKSGTIFDNFLITDDATLAEEVGNETWGQTKDPEKKMKESQEEK
ERKKLEAEEMARKEETKDEPGEEEEEEEEEELEHEEEEDEEEGETGAQEEEEESDSIKDE
L
>CALR1b XP_014045728.1 ssa03 NC_027302.1:16.022.537-16.025.752
MRGSMLFSALIALASAEPSLYFKEQFEDGDAWTTRWVESSHRSDYGKFVLTPGKFYGDPE
KDKGLQTSQDARFYSSSARFEPFSNQGKTLVIQFTVKHEQNIDCGGGYIKLFPADLDQAD
MHGDSKYNIMFGPDICGPGTKKVHVIINYKGKNHLISKDVRCKDDEYTHLYTLILNPDNT
YEVKIDNKKVESGSLEEDWDILPPKKVKDPEAVKPDDWDERERVEDPDDKKPEDWDRPEN
IADPDAKKPEDWDNEMDGEWEPPMVSNPDYKGEWKPRKIDNPDYKGKWVHPEIDNPEYSA
DSEIYRFDSIGVIGLDLWQVKSGTIFDNFLITDDATLAEEVGNETWGQTKDPEKKMKVSQ
EEQERKKLEAEEMGRKEQTKDEPEEEEEEEEEKEEELEDREEEEEGTGAEEEEEEETDSIK
DEL
>CALR1.2 XP_014024943.1 ssa23 NC_027322.1:26.361.479-26.364.218
MTTMLILLMTVLVASIFGESSVYFREEFEDGDAWKSRWVESKHRSDYGKFVHTAGKFYGD
VERSKGLQTSQDARFYSASSRFESISNKDQTLVIQFTVKHEQNIDCGGGYIKLFPADLNQ
EEMHGDSTYNIMFGPDICGPGTKKVHVIFNYKGKNHLINKDIRCKDDEYTHLYTLIVNPD
NTYEVKIDNKKVESGSLEEDWDFLPLKKIKDPDAEKPDDWDEKENIDDPEDKKPEDWDVA
ENIPDPDAKKPDDWDDEMDGEWEPPMVTNPDYKGEWKPKQIDNPAYKGKWVHPEIDNPEY
TADPEIYQYASIGVIGLDLWQVKSGTIFDNFLITNDPKLAEEVGNETWGATKDPEKKMKD
RLEEEERKKREAEEVNKKEEDEDEDEDKEERDEEEDYDDEEEEEEEEETDSKLKDEL
```

```
>CALRL1 XP_013996614.1 ssa14 NC_027313.1:41.509.871-41.518.596
MQVLGSFAIILSIFSVHSTVYFQEQFLDGDAWKTRWLDSKHKADYGEWKLTAGNFYGDAE
KDKGLQTSQDARFYAASARFEPFSNEGKTLVIQFTVKHEQKIDCGGGYVKVFPSTLDQAD
MHGDSQYYIMFGPDICGYSTKKVHVIFNYKGKNHLIKKEVKCKDDELTHLYTLILNPNQT
YEVKIDNEKVESGTLEEDWDFLPAKTIKDPEAKKPDDWDDRPKMDDAEDAKPEDWDVAEN
IPDPDAKKPDDWDEDMDGEWEPPVITNPEYKGEWKPKQIDNPNYKGAWIHPEIDNPEYAA
DSTIYKFDDISVLGLDLWQVKSGTIFDNFLVSDDVKEAEKFGAETWGVTKEPEKKMKQEE
DDKKRKEEDEKNKEQATEAEEEGEEEEGEDEGEEEETPEEGTEEEEAPGKDEL
>CALRL2a XP_014071687.1 ssa10 NC_027309.1:16.605.321-16.609.148
MRVAVAILAVFASVAVTIDATVYFKEQFQ
DGDAWKSRWLVSEHKSDYGEWKLTAGKFYGDAEADKGLQTSQDARFYALSSRFEPFSNEG
KSLVVQFTVKHEQKIDCGGGYVKIFPADLDQAAMHGDSQYYIMFGPDICGYSTKKVHVIF
NYKGKNHLIKKEIKCKDDELTHLYTLILNPDQTYEVKINNEKVESGTLEDDWDILPPKTV
KDPEAKKPEDWDDRAKIDDPTDTKPEDWEKPENIPDPDAKIPDDWDVDMDGEWEPPMIPN
PEYQGEWKAKQIDNPEYKGAWVHPEIDNPEYTADASIYKFDNIGVLGLDLWQVKSGTIFD
NFLIGDDIKEAEEFGNETWGATKDPEKKMKDAQEEEERKAREEEEKSKDTADDEGDEDED
EPEEEDDDSPTEEEEGEDPKKDKDEL
>CALRL2b XP_014004651.1 ssa16 NC_027315.1:40.259.148-40.267.862
MRVAVAIFSVFASVAVTIDATVYFKEQFQDGDAWKSRWLVSKHKTDYGEWKLTAGKFYGD
AEADKGLQTSQDARFYAMSSRFEPFSNEGKPLVVQFTVKHEQKIDCGGGYVKIFPANLDQ
AAMHGDSQYYIMFGPDICGYSTKKVHVIFNYKGKNHLIKKEIKCKDDELTHLYTLILNPD
QTYEVKINNEKVESGTLEDDWDILPAKTIKDPEAKKPEDWDDRPKIDDPTDTKPEGWEKP
ENIPDPDAKKPDDWDVDMDGEWEPPVIPNPEYQGEWKPKQIDNPDYKGTWVHPEIDNPEY
TADTSIYKFDNIGVLGLDLWQVKSGTIFDNFLIGDDVKEAEEFGNETWGTTKEPEKKMKD
AQEEEERKAREEEEKSKKDTADDEGDEDEEEDESKEEEEDSPTEEGEEEIPMKDKDEL
>ERp57a (PDIA3a) XP_014025621.1 ssa23 NC_027322.1:41.701.278-41.708.848
MLKLFFFVVLAGAALASDVIEFTDDDFDSKIGDHGMILVEFFAPWCGHCKKLAPEYEVAA
TRLKGIVGLAKVDCTVHNNVCQKYGVSGYPTLKIFRDGEDAGPYDGPRTADGIVSHLKKQ
AGPASVELKTEADFTKYVGDRDASVVGFFADDGSPAKAEFLKSASALRESFRFAHTNSEE
LLQKHSVEGEGIILFRPSRLNNKFEEGSVKFSEDTFTNAKIKQFIQDNIFGMCPHMTDDN
KDQMKGKDLLVAYYDVDYEKNPKGSNYWRNRVMKVAKGFLDQGNKLNFAVASKNSFSQDI
AEMGLDASSGELPVVGIRTAKGDKYVMTEEFSRDGKALERFLQDYFDGKLKRYLKSEPIP
ENNDGPVKTVVAENFDAIVNEEDKDVLIEFYAPWCGHCKSLEPKWKELGEKLSSDPNIVI
AKMDATANDVPSQYEVRGFPTIFFAPAGQKMSPKKYEGGREVSDFISYLKKEATNPLVAQ
EEETSKKKKKNEL
>ERp57b (PDIA3b) NP_001161991.1 ssa10 NC_027309.1:65.889.946-65.898.140
MLKLFFFIVLAGAARASDVIEFSDDDFDSKIGDHGMILVEFFAPWCGHCKRLAPEFEVAA
TRLKGIVALAKVDCTVQNNVCQKYGVSGYPTLKIFKDGEDAGAYDGPRTADGIVSHLKKQ
AGPSSIELKTEADFTKYVGDRDASVVGFFADGGSPAQAEFLKSASALRESFRFAHTNSGE
LLQKNGVEGEGIILFRPARLSNKFEESVIKFSEDKFTNAMIKKFIQDNIFGMCPHMTDDN
KDQMKDKDLLVAYYDVDYEKNPKGSNYWRNRVMKVAKSFLDQGKTLNFAVASKNSFSHDI
SEMGLDASSGELPVVGIRTAKGDKYVMAEEFSRDGKALERFLQDYFDGKLKRYLKSEPSP
ENNDGPVKTVVAENFDAIVNNEEKDVLIEFYAPWCGHCKSLEPKWKELGEKLSSDPNIVI
AKMDATANDVPSQYEVRGFPTIFFAPAGQKMSPKKYEGAREVSDFISYLKREATNPLVAQ
EEETSKKNIQIEL
>ERp57L1 XP_014016726.1 ssa02 NC_027301.1:21.616.513-21.621.161
MGTLGPFRMFLLFALAQQNVFVAASDVLELGDSDFHYTVAEYETVLVEFFAPWCGHCQQL
APEYETAATKLKGTVSLAKVDCTVNSETCGRFGVNGYPTLKIFRNGEDFAAYDGPRSADG
IVSYMKKQAGPSSVPLHNGRDLDAFVNNFDASVVGFFSGVDSSQMAEFLKASSAMRDSHR
FAHTTDLSLGLKHGVESDTVVLFRPPRLNSKFEDSLVKSDEAVSTASLRQFIRDNVFGLC
PHLTAENRENMRGRDLLVAYYDVDYLRNIKGTNYWRNRVMKVATQFQSRGLSYAVANRAE
FQEELEEEFGLGPSDGGELPLITIRNREGHKYSMQEEFTRDGKSLERFLEDYFAGKLKRQ
VKSEAASENNDGPVKVVVADNFEEIVNNPSKDVLVEFYAPWCGHCKSLEPKYTELGEQLS
ADTHIVIAKMDATANDVPPTYDVQGFPTIFFVPAGQKDQPRKYEGGREVNDFLNYLKEEA
THPLVLGTAREDL
>ERp57L2a XP_013997083.1 ssa14 NC_027313.1:61.089.980-61.110.809
MASFLSLIPPFLLSVLIFSGAAVARGDVLELGDADFDYLAAEHETMLVKFYAPWCGHCKK
LAPDFETAATRLKGTVPLAKVDCTANPDTCGRFGVTGYPTLKIFRNGEDASSYDGPRSAD
GIVHFMKKQAGPNSVTLRREADLEAFVNHFDASVVGFFSGPDSGQLAEFLKAASVMREHF
RFAHTIDMTLGLKHGVDTERVLLFRPPRLSSKFEESVLHFTETITTHTLRRFIRDNIFGM
CPHLTNENRDKLKGQDLLTAYYDLDYLQNPKGSNYWRNRVMKVGSQFASQGLSFAVANRR
DFVDELEEEFGLGASDGGDLPFVTIRTRQGFKYTMREEFTRDGKSLERFLVDYFAGRLKR
YIKSEPIPEKNKGPVKVVVAESFEEIVNDPEKDVLIEFYAPWCGHCKSLEPKYKELAEQL
YSDPNIVIAKMDATANDVPQGFDVQGFPTIYFAQASKKDQPKRYEGAHEVKDFIKYLKRE
```

ASHVPVVSGVREDL
>ERp57L2b XP_014032674.1 ssa27 NC_027326.1:12.040.425-12.049.378
MASFLSLIPAFTLSVVIFCGAVVARGDVLELGDADFDYLAAEHETMLVKFYAPWCGHCKK
LAPDFETAASRLKGTVPLAKVDCTASPDTCGRFGVTGYPTLKIFRNGEDSSSYDGPRSAD
GIVHYMKKQAGPNSVTLRSEADVEAFVNHFDASVVGFFSGPDTAQLAEFLKAAGAMRDHF
RFAHTINMTLGLKHGVDTESVLLFRPPRLSGKFEESVLRFTETITTHTLRRFIRDNIFGM
CPHLTNENRDKLKGQDLLTAYYDLDYLRNPKGSNYWRNRVMKVGYQFASQGLSFAVANRR
DFVDELEEEFGLGASDGGDLPFVTIRTRQGFKYTMREEFTRDGKSLERFLEDYFAGRLKR
YIKSEPIPEKNKGPVKVVVAESFEEIVNDPEKDVLIEFYAPWCGHCKSLEPKYKELAEQL
YSDPNIVIAKMDATANDVPQGFDVQGFPTIYFARADKKDQPKRYEGAREVKDFIKYLKRE
ASHIPVVSGVREDL
>PDIA1 XP_014032240.1 ssa02 NC_027301.1:45.673.995-45.687.434
MFKFFLLCTLAVASRADIGEEDDVLVLKKSNFEEALKAHPNILVEFYAPWCGHCKALVPE
YAKAASMLKAEGSEIRLAKVDATEEADLAQEYGVRGYPTIKFFKGGDKESPKEYSAGRQA
DDIVNWLKKRTGPAATTLGEVAQAESMIAENEVAVIGFFKDAESEGAKAFLKAAEAVDDV
PFGITSNDAVFSKFEVSKDGVVLFKKFDEGRNTFDGELSKADLLAFIKANQLPLVIEFTE
QTAPKIFGGEIKSHILMFVPKAASDFNDKMAEFKKASEGFKGKILFIFIDSEVDDNQRIL
EFFGLKKEECPAIRLITLEDEMTKYRPESEAITADNIIAFCTLFTEGKLKPHLMSQDIPE
DWDKNPVRVLVGKNFEEVVFDPKKNVFVEFYAPWCGHCKQLDPIWTKLGEKYQDSADIVV
AKMDSTANEIETVKVHSFPTLKFFPAGDEHKVVDYNGERTLEGFTKFLESGGKDGGAPAG
EEGEEDEGIDDMEDLDEQDSDSDGDGGDHDEL
>PDIA2.L1 XP_014038534.1 NW_012347234.1:27.515-33.804
MRFCVVLAAVVLVLRVSWTQTAEDTSPEQTDAAEQETDLEKKEKTTEIEEEKNVMVLHIN
NFQRALSENKFLLVEFYAPWCGHCRQLEPVYAEAARVLKGEREEERGEEEGFGLAKVDAV
EENQLAEEFDVGSFPTIKLFTDGDRNNPVDFTGKRTVQGIVQWMKRRSGPVAVALETTDA
AAEHINLHNVTVLGFFTSLESEEAKVFYSVAMEMVDMEFGVTTSPEVFQKYEIENNRVVL
FKKFDEGRVDLSVSEEVKVGEEELTVFIRTNSLELVIEFNEQNADKIFGSKIHSHSLLFI
NSTVQEQKNLLPEYRTAAKDFKGKVLFIIIDVTGPVSHVLKYFGLSEGDAPAVRIINTDT
TKKFALIGQITAATLQTFCQGVLDGNVKSHLLSEEVPEDWDKGPVKVLVGKNFEAVALEN
NKNVFVEFYAPWCGHCKELAPVWEKLAEKYADRDDIIIAKMDATTNEVEGVSVSGFPTLR
YYPAGEDSKVVEYSGTRDLETFAMFLDNGGQLPKAEEEEDDGDEEEVKDDEEDVKDDEEA
VKDDEEDDEEEVTDESSPPPANETSKDEL
>PDIA2.L2 XP_014042341.1 NW_012355960.1:107-7.487
SLESEEAKVFYSVAMEMVDMEFGVTTSPEVFQKYEIENNRVVLFKKNADKIFGSKIHSHS
LLFINSTVQEQKNLLPEYRTAAKDFKGKVLFIIIDVTGPVSHVLKYFGLSEGDAPAVRII
NTDTTKKFALIGQITAATLQTFCQGVLDGNVKSHLLSEEVPEDWDKGPVKVLVGKNFEAV
ALENNKNVFVEFYAPWCGHCKELAPVWEKLAEKYADRDDIIIAKMDATTNEVEGVSVSGF
PTLRYYPAGEDSKVVEYSGTRDLETFAMFLDNGGQLPKAEEEEDDGDEEEVKDDEEDVKD
DEEDDEEEVKDDEEDVKDDEEAVKDDEEDDEEEVTDESSPPPANETSKDEL
>TAP2a XP_014032814.1 NC_027326:10.176.036-10.180.873
MMLRMCAFAMAVGLCIDITTFCAPCFGESISETGPITFGTFGNVVRLWVVAGIRLVLLLG
LTLLTLGSIKPVFKRWLAVHCFLAPVYETGRRMLYGSSPERVYGSLGLGSPSLWLLCTAA
AAAAALFWETTFPDSNGESNGKQKTQKARVLFMRVLYFYRPDTLLLVGAFIFLSLAVLCE
MFIPFYTGKVIDILGTQYKWNNFLTAIILMGLYSLGSSFSAGCRGGLFMCAINSFTCRMK
VELFGALVKQEIGFFETIKTGDITSRLSTDTTLMGRAVALNVNVLLRTLIKTVGMLSLMM
SLSWKLTLLMLMETPITGLLQSVHDNYYQRLSKEVQDSMARANEAAGETVGGIRTVRSFK
TEQHEAGRYNDRLMDTHNLKTRRDTVRAVYLLLRRLTAVVMQVAMLYYGRLFIQRGQMST
GNLVSFILYQSDLADNIRTLIYIFGDMLNSVGAAGKVFEYLDREPQVSTKGTLQPETLTG
HVHFNNLSFSYPTRQERKVLQGFSLELRPGQLTALVGPSGGGKSTCVSLLERFYQPQQGE
ILLDGLPLQSYQHHYLHRKIAMVGQEPVLFSGSIKDNIAYGLADCSLERVQEAARRANAH
SFISQLEKGYDTDVGERGGQLSGGEKQRIAIARALIREPQVLILDEVTSALDTESEHMVQ
EALASCPSQTLLVIAHRLKTIERADQIILIDQGTVQEQGTHQELMDRKGSYYKLKERLFT
EDDAPH
>TAP2b NP_001117161.1 NC_027313:59.118.496-59.123.144
MMRRTCVFTMAVGLCIDITTFCATGLGASISKTGPISFDVFGNLVRLWVEAGIRLVLLFG
LSLLTLGSIKPVLKRWLAVHCFLAPVYETGKLMLHGSSPESPYGSLGGPSLWLLCTAAAA
AAALFWEKTFPDSKEESNGKEKTQKARALFMRVLYFYRPDTLLLVGAFIFLALAVLSETF
IPFYTGKVIDILASQYKWNDFLTAIILMGLYSLGSSFSAGCRGGLFMCAINSFTCRMKVE
LFGALVKQEISFFETIKTGDITSRLSTDTTKMARALALNVNVLLRTLIKTVGMLSLMMSL
SWKLTLLMLMETPITGLLQGVYDNYYLRLSKEMQDSMARANEAAGETVAGIRTVRSFNTE
RSEAGRYDHRLMDTHNLKTRRDTVRAVYLLLKRLTALVMQVAMLYYGRLFIQRGQMSTGN
LVSFILYQSNLGANIRTLIYIFGDMLNSVGAAGKVFEYLDREPQVSTKGTLQPETLTGHV
HFHNLSFSYPTRQGRKVLQGFSLELRPGQLTALVGPSGGGKSTCVSLLERFYQPQQGEIL
LDGQPLHSYQHHYLHRKVAMVGQEPVLFSGSIKDNIAYGLADCSLERVQEAARRANAHSF

ISQLEKGYDTDVGERGGQMSGGEKQRIAIARALIREPQVLILDEVTSALDTESEHMVQEA
LASCPSQTLLVIAHRLKTIERADRIILIDRGSVLEQGTHQELMDRKGGYYKLRERLFTED
DTSH
>TAP2c XM_014159076.1 XP_014014551.1 ssa19 NC_027318.1:51.232.531-51.243.313
MARLKTYGLVLLYDLVLWGALWTGLVLLEFSSSGGLVGLWAFGALRWALHHSITLVLSDR
KPQPVLRRWVAVLCLLPPVFESGRMVMVPASTADDLGLVPGPSMVVLALVSSTVACLAWE
LGFPDDGGNQGKEKKKKQEARAQLMRVVRYCKPDALYLAAAFLFLSLAVICETFIPYYQG
KVIDTLRGQYQHNSFMYAIGGMGLVSLGSALCSGLRGGLFMCSLSRLNKRMRHMLFRNLV
QQDIPFFEENKPGSLVSRLGYDVDKMGRSVALNSNVLVRSLVKTAGMLVLMLGLSWQLTL
LTMVEMPLLTLLQTKYNTYNQEFTKQLQDCQAQIKELASQAIGAVRTVRSFRAEEKELDR
YNQALERMYKVQEHKGIISAVHLLLRRMVTVGLKVAMLFLCRRLISAGQLSIGSVLAFVL
FQKDMVTNMKHLVYVFGDMLCTVGAAAKVFTFLDRKSDQKEAGELAPAKLQGKLAFHNVS
FYYPSRPNTPALKAVSLELHPGKMTALVGPSGGGKSSCVSLLARLYEPKEGQVLLDGQPL
HCYQHQYLHQKMALVSQEPVLFSGSIRHNIEYGLVGCTLEKVKEAAKSANVHDFICTLEQ
GYDTDVGECGGQLSSGQKQCIAIARALVREPQVLILDEATSGMDINTQHVVQGVLAGSVG
QTVLVVAHRLQTVEKADQIIFMEGGEVVEQGTHQELMASKGRYHRLKEELFED
>TAP1 NM_001123673.1 NP_001117145.1 ssa05 NC_027304.1:55.587.008-55.596.066
MPKMNFSPLLFLCMDVCVVQTVRLAQLSPLLLHHPIITLWGGSLLRAGLHLFLTFTFPGS
PPWMSSFEGLQSMGVLCFHCPLYISLLWACGQSILGQLWGWHSWQGLLQGYCVTVVACLY
WTRYVPSLLTKPTKEPQKKTGASLEKLMGYMKPYIIRFGAVLSLVVISSLGEMAIPHYTG
RMTDWIMNEDEPEAFTHAIMVMSLITVMSAVCEFVCDLIYNITMSRIHTSIQGLVFQSVL
KQEIAFFDKAQTGDLVSRITTDTNDMSESLSEKLSLLMWYFMRVTFLFLFMLNLSWKLSL
FTAMGLPIIWVIPKISGKWHQELGAKVQKSLAQANDVATETFSSMKTVRSFANEEGETER
YRKRLEKTYSLNKEEAAAYAASTWTNSMSSLFLKVSILYYGGSLVTGGTVSSGDLVAFVL
YELQFSSAVEAVMSYYPSVMRAIGGSEKIFEYVDRQPQVPPEGTLAPQNLEGHVQFKNVT
FTYPTREDTPVLKGLSLELRPGQITALVGASGAGKSTCVSLLERFYLPQEGEILLDGEPL
HSYKNQYLHDKISVVSQEPVLFARSVKDNIKYGRDDATDEEIYRAAELANAHKFISDLPN
GYDTDAGEKGGQVSGGQKQRIAIARALIRKPRILVLDDATSNLDTESEHLVHQALLKDSN
PCSVLLIAHKLSTVEKANHIVVLEEGKVLEEGSHVELLEKGGTYADLVNKQNTGFQRKEG
EELKNENKGSNPMTLLNLH
>TAPBPa XM_014177071.1 XP_014032546.1 ssa27 NC_027326.1:10.023.358-10.039.557
MIQEKSLLYINTDPESEETKSQQGPSADINHDRVYYVTDPAAILCSSSLHPPEGSVHKPQ
CEINPFMPQPSTVQWVVPLTDSAHSPIYLQADWYSAALQGLDGQLGLSSVMRAPTATKEP
TVVLSVSSRTPLVHSRLGEPVVLDCGFWMEATSPLSGSGFAVEWRYQFRGDGRLVLAYDG
KTDRFAETKEKRAGLDFTALHETGNASLILQEAQVRHTGTYICTVYLPYLLAQVAVELEI
VEPPSLSIFPSPLPLSVPGQVVKVQCEASGFFPLSLDFHWELTGPDGKVRPLGQGSVTGH
RQGPDNTYSQTSRLELDSAKLDLGRGGEVTCVAVHPGGTRRASVTLNVIGINGPSIEDSM
AMVAVALGLYGLIKIVSWTFSSGSDDTNSQEKKVK
>TAPBPb XM_014141751.1 XP_013997226.1 ssa14 NC_027313.1:59.041.174-59.053.860
MTNISTILQSFLAFSYFMHVYGASCPVLECWFVQEKPGGGFHAAMSQEKSLLYINTDPDS
EETRSKQGLSTDHDRVYYVTDPAATLCSSSMHPPEGSVQKPQCEINPFMPQPSTVQWTVP
LTDSAHSPIYLQADWYTAALQGFDGQLRLSNVMRAPMATKEPKVLLSVSSRTFMIRSRLG
EPVVLDCGFWVDASSPLSGSGFAVEWRYQFRGDGRLVLAYDGKTDRFAEIQEEGAGLDFT
ALHETGNASLILQEAEVRHSGTYICTVYLPYLLAQVAVELEIVEPPSLSIFPSPLPLSMP
GQVVTVQCEASGFYPLSLEFHWVLTGPGGRVSPLGQGSVTGHRQGPDSTYSQTSRVELDS
AKLDLGRGGEVTCVAVHRGGTRRASVTLNITGVSAPSIEDYMAMVAVALGIYGLIKVVSW
TFSSGSDVAYTQEKKVK
>TAPBPc XM_014141493.1 XP_013996968.1 ssa14 NC_027313.1:50.968.346-50.981.042
PAATLCSSSLHPPEGSVQKPQCEINPFMPQPSTVQWTVPLTDSAHSPIYLQADWYTAALQ
GFDGQLRLSNVMRAPTATKEPKVLLSVSSRTFMIRSRLGEPVVLDCGFWVDASSPLSGSG
FAVEWRYQFRGDGRLVLAYDGKTDRFAETQEEGAGLDFTALHETGNASLILQEAQVRHSG
TYICTVYLPYLLAQVAVELEISNKVIEINPYLLGTMSGSAADCQYWERLLAKECRLYKLR
NKQRISVSAASKLLCNMMLGYRGMGLSMGSMTVGWDNKLDK
>TAPBPL1a XP_014069540.1 ssa09 NC_027308.1:102.783.830-102.789.947
MSLNVNILLCLFLCGEVPGIQSFEQVPWLPCQLVDESVTFNDEGHAETEYQHRDAGLQFG
HPGDSALNPNTITFLVTGSKVDMRKYIEGVVEHQLQCEIRRYSTESPQMRWPGLGAQEHD
IWFTCTIRHTDGVFIITSFLRHTPATPTPGQAHYLNWAAIADRAALTTSTVMLVLTRSPS
VRVGLVKQQSLHCQFDVDHKAADLTVEWRFQRRGERTTLFSHSSRSGQTEGSGVPLNAIG
RGDASLTLPLTKQSSEGTYVCHVSVPPLFGSHDITLQIMESPRVSLNVDSTISLVDRGEQ
KVVCEAEGYYPLDVEMEWFREPSGGGLLPEKLDTVLYSSHRHHQDGTYSLSAFFLLHASL
HDSGSKYFCRVSHSSQRMPIRKSFTLNVTEYDSWNAALWFFSGFGFILVMVATLFVMLPR
LSSARKANQRKPY
>TAPBPL1b XP_014017660.1 ssa20 NC_027319.1:46.797.073-46.803.184
MSLNVNIVICLFLCAEVPGIQSLEQVPWLPCMLVDERVKFNDEGHAETQYQHRNAGLQFG

```
HPGDSALNPNAITFLVTGSKVDMRKYIEGAVVEHQLQCEIRRYSTEGIQVRWPGLGAQDH
DIWFTCTLRHTDGLFVITSFLRHTPATPTPGQADYRNWAAIADREMLTTSTVMLVFTRTP
SVWVGLMKQSSLHCQFDVDHKAADLTVEWRLQRRSERTTLFSHSSRSGQREGGGVELKGI
GRGDASLTLPLTKQSSEGTYVCLVSVPPLFGSHEIALHIMEPPRVSLNVDSVISLVDTER
QKMVCEAVGYYPLDVEMEWFREPSGGGGGLLPEKLDTVLYSSHRHHQDGTYSLSAFFLLH
ASLHDSGSKYFCRVSHSSLRMPIRKSFTLIVTEVDSWNFLWLFFGCGFILVMVATLCVML
PRLSSARKANKRKPY
>TAPBPL2 XP_014062182.1 ssa07 NC_027306.1:12.926.171-12.930.490
MKTFTVLLCLIPYAGVLGFLQVQWLRCRLKDEYVWTNDEGHIETNNSYRDAVLQFENSGN
SALLSESITFLVAASKVDMRKFVEGPVDQLQCDIHRYSKGMSRVRWPSLGGTGHDIWFTC
TLRHTAGLFNITSFLRVTPATTMSAHQPDFLSWLTIGVKEQISASAVMVLMTRSPSVRVG
LQEERTLHCQFAVDHKVPHLTVEWHLQRHGARRTKLFSYSSSSGQTEGSGVAVKGIAGGD
ASLTVPITKVSSEGTYVCSVRVPPLNGSVDIALHIIEPPNVSLTTKEEQIPRVVCEANGF
YPLDVDIDLFKETSSGQRPEKLGNVLHSSHIQHHDGTHSVAAFVRLRPSPQDSGCETYSC
RVSHVSLQGLYIHKSIIGPGCWTWIHLTLPGLVCLIFVIIFIAVTRQRFF
>TAPBPR NP_001133983.1 ssa02 NC_027301.1:10.225.492-10.231.249
MLEILLFGYLITCVSGQGGADVVLSCSLVEEGSGMGGMGGGALFSRTPAMLVLRDLAVTP
DLSPDTLTPFNPPAVPDPDNIILEAKVESPEIPEADLLLHADCNEQEVTCEISRYFPRNA
KEGSTEPAYFIGSLQIEGGGLSLTLILQTLPLDQSDRPALMQSKLELPLSQSGTLLTEVV
FLVFSRSESHSAPLGGVALLDCGFRQQAPPPGWELGLEWRLQHRGSGRKVLEIRAGQTET
EEGPAVHVEREGSSVDAALLVGQGNASLTLARLKVSDEGTYICTVSTGLYQAQQVIQLHV
TQPPRVSLSEEKLVFRDELPQKLSCHCKNYYPLDVQMEWISVSSTDSEPSVLSDQVSLSS
HRQHSDRTMSISSHLTLHPSTFPPGTTVTCRVTHPALDTPLSLSLTVETPEPDSYWMVLG
FLVITVLFFYQVMK
>ERAP1 XP_013998888.1 ssa15 NC_027314.1:2.525.189-2.544.613
MRTLTVTILVLLHVSFAPSLAAQLPGDHDTNDKSSSLLPPIATNGQPFPWIHMRLPETVS
PIHYDLLVHPNLTSLDFTGEVQIQLQVFEDTSTIILHSKDLQIAKAELLAPEGPGSLPVP
LQVLEYPAFHQLALMSDVLLVRGGMYKVRLEFSANLSDSFHGFYKSSYRTTKGEVRFMAS
TQFEATSARAAFPCFDEPAFKANFTIQIRRESRHIALSNMPKVKTVELPGGVLEDHFDTS
VRMSTYLVAFIVSDFQSVSKTTSHGVKISVYAVPDKINQTDFALNAAVRLLDFYDDYFDI
PYPLPKQDLAAIPDFQSGAMENWGLTTYREAGLLFDPNKSSASDKLGITMVIAHELAHQW
FGNLVTMQWWNDLWLNEGFAKFMEFVSVNITNPELQVNDFFLGKCFEAMEVDSLSSSHPV
SSQVDTPTQIQEMFDDVSYDKGACILNMLRDFLTPEAFKIGIVRYLRRYSYQNTVNSHLW
ESLTNICQSDDLDEGRLKDEGFCSQEKAQSGAPKWYSGDQLDVRAIMDTWTLQEGFPLVT
VEVRGRQVRLSQERYLKTDDPSQTHGFLWQVPLTYITSSSSTVHRFLLKTRTDVLYLPEE
VGWIKFNVDMSGYYMVHYEGEGWRSLTSLLLTNHRALSSNDRASLINNAFQLVSVGKVGL
DTALDLSLYLSKETDIMPVTQGLGELVPLYKLMEKRDMEGLENQMKGYIVELFRGLIDRQ
TWSDDGSVSQRVLRSYLLLFGSVRNHPPCVATATHLFNKWRASDGNMSLPSDVSLAVFAI
GARDPEGWDFLFEKYRHSQHTSVKSRIKSALSISPLQHKLKRLMEQSLAGEVMKTQDLPH
VVISVSRNPKGYKLAWDFLRHNWHTLVKKFDLGSHSISGMVTGVTNQYSTREMLDEVRGF
FDSLSEETGSGLRCIQQTYESIEENIRWMDQHLPQLKAWLDRQAQGARTETQGHEDL
>ERAP2a XP_014026228.1 ssa24 NC_027323.1:15.056.418-15.064.045
MVRFLVLALLSLAGVTQTSASPTQASEPPNPTEEQPPLGTGSLSFPWSHLRLPGYIVPLH
YHLLLHPNLTMLSYSGTVRIELQVQNNTNWVVLHSKGLRITTATMLDQNLAHLSDRVLPV
LHNPTHEQTAIFSPRVLSGGQKYFLFLEFGAELGEGFYGFYRSTYRTSAGETRNLASTHF
EPTSARMAFPCFDEPSFKANYSISIRRSLAHTALSNMPVEQTVVLDDGLMEDRFAVSVRM
SSYLVAFIVCDFRSVSATTASGVKVSVYAAPEKWQQTHYALKAAVKLLEFYEKYFNIKYP
LPKQDLVAIPDFQAGAMENWGLITFRETSLLYDPATSSASDRVWVTMVIAHELAHQWFGN
LVTMEWWNDIWLNEGFARYMEYISVNATYPKLRVEDYLVDTCFAAIGRDSLNSSRPISSA
AESPTQIAEMFDTVSYDKGACVLHMLRHYLTDQVFQSGIVRYLRRYSYSNAHNQDLWDSL
ANTCSEEEFTSGEHCYSSRQAAKNAYLYAGEHLDLTTMMNTWMLQTGVPLVTVTRQGSRL
LLKQERFLRTAHPSDPAWPSLQQGFLWHIPLTYRTDTSTSIHRHLMTTLTDSVDVGEEVG
WVKVNVDMAGYYLVHYDGSGWDNLIQLLKDNHTALSFMDRTHLIHNAFQLTTAGRLSLDK
ALDLIGYLRSESHTVPLLEGLGYLEAFYRMVERRDIPDVTQNLRTYILWYFRDVIDRQTW
SDKGSVSERRLRSELLSLACHLGDLPCLKQAQRSFTHWLDSNSTLNLPADVAETVYSVGA
QEDTGWASLLQTYTHSLSETHKRKILSALASSRDTNKLTRLLELGVEGEVIRTQDLDSLI
VMVARNPRGHHLAWSYVQKYWSTLVDKFQLGSFSIRNIIIGTTAQFSSTEELTEVRVFFE
SIHEQASQLRVTEVAMDNVQKNILWLQRNLGTLRSWLDQQID
>ERAP2b XP_014016590.1 ssa20 NC_027319.1:28.634.401-28.645.787
MFWVRFLFLALLSLAGVTQTSSSPTQASEPPNPTEEQPPLNTGSLSFPWSHLRLPEYIVP
LHYHLLLHPNLTILSYIGTVRIELQVQNNTNWVVLHSKGLRITTATVLDQNLAHLSDQVL
PVLHNPTHEQVAIFSPRALTGGQKYFLFLEFGADLGEGFYGFYRSTYRTSTGETRTLAST
HFEPTSARMAFPCFDEPSIKANYSISIRRSPAHTALSNMPVEQTEVLDDGLMEDRFAVSV
RMSSYLVAFIVCDFRSVSATTASGVKVSVYAAPEKWQQTHYALKAAVKLLEFYEKYFNIS
```

```
YPLPKQDLVAIPDFQSGAMENWGLITFRETSLLYDPTTSSASDRLWVTKVIAHELAHQWF
GNLVTMEWWNDIWLNEGFATYMEYISVDTTYPKLRVEDYLLDTCFVAIGRDSLNSSRPIS
SVAESPTQIKEMFDTVSYNKGACVLHMLRHYLTDQVFQSGIMRYLRRYSYSNARNQDLWD
SLANTCPEEEFTSGGHCYSNSQAAKNAYLYAGEHLDLTTMMNTWTLQTGVPLVTVARQGS
RLVLKQERFLRTTHPSDPAWPSLQQGYLWHIPLTYRTDTSTSIHRHLMTTLTDSVEVGEE
VGWVKVNVDMAGYYLVHYDGSGWDDLIQLLKNNHTALSFMDRTHLIHNAFQLTTAGRLSL
DKALDLIGYLRSESHTVPLLQGLAYLEAFYRMVERMDIPDVTQNLSTYILWYFRGVIDRQ
TWSDKGSVSERRLRSELLSLACHLGDLPCLEQAQRSFTHWLDSNSTLSLPADVTETVFSV
GAQEDSGWASLLHIYTLSLSETHKHKILSALASSRDTNKLHRLLELGLEGEVIRTQDLDS
LIVMVARNPRGHHLAWSYVQKYWSTLVDKFQLGSFSIRNIIGTTGQFFSTEELTEVRVF
FESIHEQASQLRVTQVAMDNIQKNILWMQRNLGTLRSWLNQHID
```

SF5.11 Deduced MHC class I amino acid sequences

```
>UBA*0201 AF504023.1 AAN75117.1 ssa27 NC_027326:10.122.009-10.149.394
MGIVNGHQIDHYDSITKRAIQKAEWISGAVDPDYWKTNTQIYAGTETVFVNNINVAKSRF
NQTGGVHVNQKMYGCEWDDETGVTEGFDQDGYDGEDFLAFDLKTLTWIAPTPQAVITKLK
WDSNTAQNEYRKNYLTQTCIEWLKKYLDYGKSTLMRTVPPSVSLLQKTPSSPVTCHATGF
YPSGVMVSWQKDGQDHHEDVEHGETLQNDDGTFQKSSHLTVTPEEWKNNKYQCVVQVTGL
QEDFIKVLTESEIKTNWNDPNIVLIIGVVVALLLVVVAVVVGVVIWKKKSKKGFVPASTS
DTDSDNSGRAAQMT
>UBA*0301 XM_014177344.1 AAN75116.1 ssa27 NC_027326:10.122.009-10.149.394
MKCFILLLLGIALHSSSAATHSLRYVYTATSGIPDFPEFVTVGLVNGEPISYYDSIIRRE
TPRQDWMAKTEGSDYWESQTQVSIGSEQTFKANIDVAKQRFNQTGGVHVNQKMYGCEWDD
ETGVTEGFDQDGYDGEDFLAFDLKTLTWIAPTPQAVITKLKWDSNTAQNEYRKNYLTQTC
IEWLKKYLDYGKSTLMRTVPPSVSLLQKTPSSPVTCHATGFYPSGVMVSWQKDGQDHHED
VEHGETLQNDDGTFQKSSHLTVTPEEWKNNKYQCVVQVTGLQEDFIKVLTESEIKTNWND
PNIVLIIGVVVALLLVVVAVVVGVVIWKKKSKKGFVPASTSDTDSDNSGRAAQMT
>ULA XM_014177345.1 ABQ13870 ssa27 NC_027326:10.037.566-10.054.430
MKCFILLLLSISLHAASAAMHSLRYVYTATSGMPDFPEFMTVGLVNGEPISYYDSIIRRE
TPRQDWMKEAVDPDYWNRNTQTSIGDEQTFKANIDVAKQRFNQTGGVHVYQNMYGCEWDD
EAGVTEGFDQYGYDGEDFLAFDLKTLKWIAPTPQSLITKLKWDNNMAQIQQDKHYLTQTC
IEWLKKYLDYGKSTLMRTVPPSVSLLQKTPSSPVTCHATGFYPSGVMVSWQKDGQDHHED
VEYGETLQNDDGTFQKSSHLTVTPEEWKNNKYQCVVQVTGVKEDFIKVLTESEIKTNWGN
TNIGFVPANTSDVGSNSSHNTVPKE
>UDA XM_014141492.1 ACY30371.1 ssa14 NC_027313.1:50.992.799 -50.986.338
MKGFILMFMGTCHLFEAFGVTHSLKHFYTASSKVTNFPEFMVVGMVDGVQIDHYDSNIQR
MVPKQDWMNKQTEAEYWERETGIAFDSQQVFKDDVNILKQRFNQSGGVHVLQYIYGCSWD
DETEQRDGFGQLGYNGEDFLVYDMNTLTWKALKQQADVMRDKWNRDISRLVFWKTYFSQT
CIECLKKQVVNGKSTLRTAPPSVSLLQKTPSSPVTCHATGFYPSGVMVFWQKDGQEQHED
VEHGEILHNDDGTFQKSTHLRVTPEEWKNNKYQCVVQVTGIKEDFIKVLTESEIQTNWGD
PAPIIVPIIGGVVALLLVVVVVGVVIWKKKSKKGFVPASTNDTDSVYSGKDLLKT
>UGA XM_014141729.1 ACX35601.1 ssa14 NC_027313:59.497.676-59.507.931
MKTRLISAMKIYFVLLSCIHGALSVIHSLRYFYTSSSGISDFPEFVDMGMVNDQVISHYD
SITKRKVPKQSWMGKVFDQQYWDSTTEDLRGAEKVFKNNLQTAQKRFNQTGGMHISQDMY
GCEWDDETGLTEGFHHIGYDGQDLLVFDLKRATWIASVPQALHSKMKWEGDPSSIESEKR
YLTQDCIVWLKKYLEYGKTTLQRTVPPSVSLLQKTPSSPVTCHATGFYPSGVMVFWQKDG
QDHHEDVENGETLHNDDGTFQKRTHLKVTSEEWKNNKYQCVVQVTGIKEDFIKVLTESEI
QTNRGVNTIGSAPIIGVVVALLVVVVVVVGLVMWRRKKSKKGFVPASTSDTDSENSGKGA
QKI
>UHA1 XM_014165549.1 ACY30367.1 ssa21 NC_027320.1:48.663.107-48.675.014
MIVLNMYSVSNRIVHISTLLVTYLIPMTFAANHSLKYFYTALPQSTGLPEFSAVAYLDEE
PMYFYDSSTKEVVARQEWVKGAVDPDFWRRNTQILKENEMVFKDNMDTARDRFNQTSALV
LQKMYSCDWDEVTGATDEREQYGYGGEDFLLFDLKNKRWIVPGRQGLITKMKWDANVIKL
EAKIHYLTHTCIEWLKKYVSNWRRNLERTVPPQVSLLQKEPSNPVTCHATGFYPNAIMIF
WGRDGVEIHADVVHEETLPNGDGTYQKRIHLTVSPEDLQQHNYTCTVQHVRGDDVVLSAN
RDSIRSNSRNTQAKTAEMEKPKKRHSADTLQIQLVRIFIPVFV
>UHA2 XR_001323306.1 ACY30368.1 ssa21 NC_027320.1:48.681.967-48.696.230
MGGQILVLWLCLSLRTANSATHSLKYFYTALPQSTGLPEFSAVAYLDEEPMYFYDSSTKE
VVARQEWVKEAVDPDFWRRNTHIFKETEKVFKYNMDSARDRFNQTSALALQKMYSCDWDD
```

VTGAATDGREQYGYGGEDFLSFDLKNERWIAPGRQGLITKMKWDANVIKLKAKIHYLTHT
CIEWLKKYVSNRRRNLQRTVPPQVSLLQKEPSNPVTCHATGFYPNAIMILWGRDGVEIHD
DVVHEETLPNGDGTYQKRIHLTVSPEDLQQHNYTCTVQHIRGDDVVLSANRDSIRSNSRN
TQGHYITIVLVTLAFSVIVVFIMTKVCKGKEPTAGKEKQCPSEY
>SAA S lineage BAC: FJ969488.1, ACY30362.1
MITTILISFMQFSIVAPHSLHRHCIATQGTLYPKNIQLVMIDDVTVYYYNSSAEQEAVVP
EWLNHLEGIEFWQEVNRNLKFSRFVMDTAVRVTSEHYNHSHDHFYQAHGRCGWKSDGTTE
AFMSHAYDGKDFVSFDVSTRTWTAAVSHAVFYKRKRETDLEDLVRLVIHYESGCIRWLKK
LLQFSVTFREPKVPAVSLFERPPHGNSEVEVTCHVTGFYPRAVQVEWLGAEGLPMVDGVS
SGEVLPNGDGSYQLRKSLTVPQEAQDTQSYSCLVLHSSIAGNITVTWAPKKNLANVLMAI
VIIVSVVLILTVLFKYLVRRRAVGKSQS
>LCA L lineage AGKD03039122.1:3,341-4,520, transcritpome match
MGKLSVFLFVLSFYTIVNSGSGSHSLWALATYISGETPFPEFTVVVMLDDVQVAYYDSNM
KHFIYRGHNTPNKIHDDEAKNGDFVFGVMYHHMKERYFHLKHHLNLTEGVQVQQRMAGCE
MFDNGEPALIMTKNTFNAVFADHAIYYNITHFTYDAGKLLQGWDGMRQAQEKILYENVLL
TLCIRTLKTILKREKNIVMRKVPPRLRLIKKEVSGGFQVSCLVFGFYPRHINLTLLRDGQ
PVAEQELTGGEVLPSGDGTYQLRKSLEVSTEELKKRHNYTCTASHLSLDNKLDVSWESGA
ERVHLSTLSVLLVMLLILILLVTFICVKRRWSNTASQSELANVDAKVSEEMNLSSDSEN
>LDA L lineage AGKD03037778.1:12,196-13,372
MGKLSIFLFVLSFYTIVNAGSGSHSLWALATYIIGETPFPEFTVVLMLDDVQIGYYDSNI
KQSVYRGYHITDKMNDEAQDGTYVLGTMYDHMKERSFRLKHHLNLTEGVHVQQRIGGCEI
LHNGEPALIMTKNSFNAIFEDYAVYYNMTHFTYDSGKLLLGYNWIRQATERTLYANVWLP
ICINTLKKCLKRENFVMRRVPPRLRLIKKAVSGDIQVICLAFGFYPRHINLTLLRDGHPV
AEQELTGGEVLPSGDGTYQLRKSIYVSTEELRERHNYTCTASHLSLNNKLDVSWESGAER
VHLFILSAPLVMALIVILFCIFICLVRRIRAASQNLLQLASVDALEADEMNLSSDSEKT
>LFA L lineage AGKD03073275.1:8,761-9,894 and EG847842
MGKLSVLFFILFYTIGNAGSGSHSLWALATYINGETPFPEFTVVVMLDDVQVGYYDSNMK
DFIYRGHNPTDKIHDDVAQDGAYVFGIIYQSIKERSFHLKHQLNLTEGVQVQQRMSGCEM
LDNGEPALIMFKETFNGIFVDHAIYNSMTHFTYDSGTLLLGYYGIRQAYEKALFENVLLP
ICIKNLKTILKREKNVVMRKVPPRLRLIKKEVSGGLQVSCLAFGFYPRHINLTLLRDGQP
VAEQELTGGEVLPSGDGTYQLRKSLEVSTEELKKRHNYTCTASHLSLDNKLDVSWESEAE
RVHLSTLSVLLVMLILILLGIFICVKRRWRCTASHLKLVNVDAKA
>LGA L lineage AGKD03064454.1:3,875-6,336 and DY733800
MGKLSVFLFVFSFYTIVSPGSGSHSLWALATYIVGETPFPEFTVVVMLDDVQVAYYDSND
KQSVYRGQHITKTKDDEAQDGAHVFRVIYQSMKDRSFELKHRFNLTEGVQVQQKITGCEM
LNNGESALVMYKDVFNAIYTDRTLYYNMTHFTYDAGKLLLGWDGIRQAYERTLYENVYLP
ICIKSLKRLLKREKNIVMRKVPPRLRLIKKEVSGGFQVSCLAFGFYPRHINLTLLRDGQP
VAEQELTGGEVLPSGDGTYQLRKSLEVSTEELKKRHNYTCTASHLSLDNKLDVSWESGAE
RVHLSTLSVLLMMLLILILLGIFICVKRRWSNTASQSELANVDAKVSEEINLSSDSET
>LHA L lineage AGKD03049350.1:3,883-6,976, transcriptome match
MGKLSVFLFVLSFYTIANAGSGSHSLWALATHIIGETPFPEFTVVVMLDDVQVGYYDSNM
KHFIYSGHNPTDKIHDDVAQDGAYVFGTMYQSIKERSFHLKYHLNLTRGVQVQQRMAGCE
MLNNGEPALIMSKNTFNAIYTDRTLYYNMTHFTYDAGKLLPGWDAMRREYLRILFGNVFL
PICIKTMKTFLKMEKNVVMRKVPPRLRLIKKEVSGGLQVSCLAFGFYPRHINLTLLRDGQ
PVAEQELTGGEVLPSGDGTYQLRKSLEVSTEELKKRHSYTCTASHLSLDNKLDVSWEPGA
ERVHLFTISILLMMLLIVILLGIFICVKRRRCTASQAFVTSCQHGGH
>LIA L lineage AGKD03025882.1: 26,896-28,189, transcriptome match
MAKLCFFLILLSLYTIVNAGSHSLWAFATCISGEAPFPECSVVLMQDDIQVGYFDSNKEQ
FIHKGPYAPDETEVEEAQDAAYVFGHMFLSMKRRLSDLRYRFNSTGNIDVQQRMAGCEML
DTGEPGLILSTDAFNAILADLIYYNMTHYSYNSGNLLSPWSEVHQTYTKWHYQTIYLPVC
IKTLKRFLERLKNFVMRKVRPRVRLIQKAMSGGACVSCLAFGFYPRHINLTLLRDGQPIV
EQEMTGGQLLPNGDGTYQMRKSLEVNTEELRERHNYTCTTSHLSLDNKLDVSWIPESGMD
RVGLYVKSAPLATVAIIILLSIFVCVRRRNTAGSQTLSQLSNANDAQVAEQISLSSHSET
>LJAΨ pseudogene L lineage AGKD03038500.1:1,831-3,283
SGSHSLWAFATYIIGDTPFPEYTVLLLLDDIEVGYYERQFVYRGHNALDEKEMGIVLDIA
SVFGAMSFSMKGRSYDLKQLFHFTEGIHVQQRMACCEMLDNDKLVLILSRSTFNKIVADG
MCYNMTQNTYYTGNPQLAWDEVKLEYVKMLYAHVYLPLCIKTLNIFLEREKNIVMRKVRP
RIRLIKKAKSGGLQENDRLTARIAVLQAQLQTQSLGKGNFSVGKDETASVPP
>LKAΨ pseudogene L lineage AGKD03020594.1: 773-1,321
ECSPSHLHQNTEDSPEEREERCDAKVPPRLRLIKKEISGGFQVSCLVFGFYPRHINLTLL
RDGQPVAEQELTGGEVLPSGDGTYQLRKSLEVSTEELKKRHNYTCTASHLSLDNKLDVSW
ESGAERVHLSTLSVLLVMLLILILLVTFICVKRRWSNTASQSELANVDAKVSEEMNLSSV
SET
>LLAΨ pseudogene L lineage AGKD03683246.1:93,581-96,980

```
HPLSGSGSHSLWAFATYISG*TPFPTVVLMLDDIQVGYFDSNIKRYIHKGYNASEETEVE
EAYHIAFIFGPMSFSLRARSSELKRFNS*EGIHVQQRLAGCEMSDNGEPALIVSSLNGIY
ADSAIYYNMTHYSYNSGKLFPPWSEVHQTYS
>LMAΨ pseudogene L lineage AGKD03030462.1:91,197-91,772
YVKGLYQTIYLNICMDTLKTFLEKEKKCIMHKVHARVRLIQKDMSGGVQQMSCLPFGFYP
RHINLTLLRDGRPIAEQVLNGGAAAAQWRRHVPAEEESGGQYTGTKRDTTTPALPPTSVW
TTRWMSAGYLSLEQTE*VCLSCQLYWSITILICISVCLRKRSEAGSQTLSQLSNAVDAQV
DEQISLSSHSAT
>ZAAa XM_014177297.1 ACX35596.1 GQ505858.1 AGKD03017891.1:178,619-195,734
MNISHLTVFVLYFSLECICQSDTYSLSYIYTALSKPVDLPGIHEFTAMGLMNNQQIDYYD
SVSKKKIPKQDWMREKLPADYWEKGTQSRKSKEQWFKVNVDILMKRMRHNNTDVHVLQWK
VGCEIDQQSDGTLKFIKGIDQYSYDGDDFLAFDDVTMQWVAPVDQALPTKRKLDDVQILN
TYTKGYLEKECVDWLSKFMEYEDKEFSWADSAPKVYAFAKKAKTAGHVRLTCMATGFYPK
DVVMHIKKNGVPLTDRDGVQSAGLLPNDDETYQIRMSVQIPEADKETYECYVNHRALKEP
IVVKWDGKCCDCSSGGAVVIGAVVIAFIVVLILVGLFVLHRRGTIGRS
>ZBAa XM_014177293.1 AGKD03017891.1:51,744-55,896
MNSGAAMARRPVTSTAECRTPPEQGEEPTSAEVVTSDIYSLNYIYTALSKPVDLPGIHEF
NAMGLMNNKQIDYYDSVSKKKIPKQNWMREKLPADYWEKGTQSRKSKEQWFKVNVNILMD
RMRHNNTNVHILQWKHGCEIDQQRDGTVKFIKGIDQYSYDGDDFLAFDDVTMQWVAPVDQ
ALPTKRKWDGVQILNQYTKGYLEKECVDWLSKFMEYGDKEFSRADSAPKVYAFAKKAKTA
GHVRLTCMATGFYPKDVEMNIKKNGVPLTKHDGVQSAGVLPNDDETYQIRMSVQIPEADK
ETYECYVHHRTLEEPIVIKWDPLKHCQVEWGASIHIYFQVSRDVRSGSSLGSCWVTQGHS
EPCPEANPALSWLCA
>ZCAa XM_014177292.1 AGKD03017891.1: 38,609-40,974
MSAFKMYYVVALLLLFATLSTEDTVETWSLNYIYTALSKPVELPGIHEFTAMGLMNDKQID
YYDSVAKKKIPKQDWMREKLPADYWEKGTQSRKSKEQWFKVNVNILMDRMRHNNTDVHIL
QWKHGCEINQQSDGTLKFIKGTDQYSYDGDDFLAFDDVTMQWVAPVDQALPTKRKWDGVQ
ILNQYTKGYLEKECVDWLSTFMEYGKKHLRMDDSAPKVYAFAKKAKTAGHVRLTCMATGF
YPKDVVMHIKKNGVPLTKHDGVQSAGVLPNDDESYQIRMSVQIPEADKETYECYVYHRTL
EEPIVEKWDGKFYDCNQVTGVIIGVVAVVLLFIVVTPLLVLWKKGK
>ZDAa XM_014177296.1 AGKD03017891.1: 9,374-13,096
MEIIVEAAEQFLGLKDSAKEYDGRYGELKEVVRVLFYIYSLNYIYTALSKPVDLPGIHEF
TAMGLMNNQQIDYYDSVSKKKIPKQDWMREKLPADYWEKGTQSRKSKEQWFKVNVNILMD
RMRHNNTDVHILQWKHGCEIDQQSDGTLKFIKGTDQYSYDGDDFLAFDDVTMQWVAPVDQ
ALPTKRKWDGVQILNQYTKGYLEKECVDWLSKFMEYGEKEFSRPDSAPKVYAFAKKAKTA
GHVRLTCMATGFYPKDVVMHIKKNGVPLTDRDGVQSAGVLPNDDETYQIRMSVQIPEADK
ETYECYVHHRTLEKPIVIKWDGICCDCSSFNAVVIGAVVITFIVVLILVVLFVLHRRGTI
VIPGLRTTATGNGVAFSGVNTS*
>ZBAb XM_014141712.1 ACX35613.1 GQ505860.1
MYTFMLFVFYFSTECIVQSQSEIYSLNYIYTALSKPVELPGIHEFTAMGLMNDIQIDYYD
SVDKKKIPKQDWMREKLPADYWEKGTQSRKRKEQWFKVNVNILMERMRHNNTDVHVLQWR
HGCEVDKQPDGTLKFMKGIDQYSYDGDDFLAFDDVTMQWVAPVDQALPTKRKWDGVQILN
QYTKGYLEKECVDWLSKFMEYGEKHFSSTDSPPNIYVFTKKAKPAGNVHLTCMVTGFYPK
DVIIHFKKNGVQLTEDDGVLSTGARPNNDDTYQIRISVQIPEADKDMYECSVSHAMLKEP
IVEKWGAGNTGAPPPTGIQASLIGNGNGATNLNLTTTPSKTLFQYL
>ZCAb XM_014141708.1 ACX35618.1 GQ505860.1
MYTFMLFVFYFSTECIVQSQSEIYSLNYIYTALSKPVELPGIHEFTAMGLMNNRQIDYYD
SVDKKKIPKQDWMRDKLPADYWEKGTQSRKSKEQWFKVNVNILMERMRHNNTGVRILQWK
HGCEVDKQPDGTLKFIKGTDQYSYDGDDFLAFDDVTMQWVAPVDQALPTKRKWDGVQILN
QYTKGYLEKECVDWLSKFMEYGEKHFSSADSPPDINVFANKAKTAGNVHLTCMATGFYPK
DVIIHFKKNGVQLTEDDGVLSTGARPNNDDTYQIRISVQIPEADKQTYECSVSHITLVQP
IVVKWGKVVLVFNILYIFSTNQLFAWLSIMVKSISKMFVCYKVTNADLNVNQQLFQAHCI
GFCC
>HAA XM_014139619.1 XP_013995094.1 and TSA: GEGX01039681
MRLPLTFILFFKLNWGLPIDTVILFQRSGVVGEMDTVEQVLVNGAPLSSCGKYVSGILRA
VLLGNDSESFQQAFEMEENVTNTITKNPTYQYLRVRECKLNGFRVVHLTDQLLVNGSDFL
TLDQSTVTWTAEVPQALALKQLWDWDTERTRRERMQLHESCTELMKELTHSEPTTNRAGM
SLLTVLAPLLASLAFVAVVIASFLIANRQDPRVSGHPGGVLGSIVHYPQNVTETPQAPQS
GKDYQVL
```

SF5.12 Proteasome subunits PSMA, PSMB, PSMEs

>PSMA1.1 XM_014124690.1 XP_013980165.1 ssa10 NC_027309.1:67.050.826-67.063.258
MFRNQYDNDVTVWSPQGRIHQIEYAMEAVKQGSATVGLKSRTHAVLVALKRAQSELAAHQ
KKILHVDEHIGISIAGLTADARLLCNFMRQECLDSRFVFDRPLPASRLVTLIGSKTQIPT
QRYGRRPYGVGLLIAGYDDMGPHIFQTCPSANYFDCKAMSIGARSQSARTYLERHMETFL
DCNLNELVRHGLLGLRETLPAEQDLTTKNVSIGIVGKDMEFTIYDDDDVGPFLEGLEERP
QRKVIEAADEPAADKPDEPMDI
>PSMA1.2 XM_014175942.1 XP_014031417.1 ssa26 NC_027325.1:26.390.052-26.394.352
MFRNQYDNDVTVWSPQGRIHQIEYAMEAVKQGSATVGLKSRSHAVLVALKRAQSELAAHQ
KKILHVDSHIGISIAGLTADARLLCNFMRQECLDSRFVFDRPLPVSRLVTLIGSKTQIPT
QRYGRRPYGVGLLIAGYDDMGPHIFQTCPSANYFDCKAMSIGARSQSARTYLERHMDTFL
DCELNDLVQHGLRGLRETLPAEQDLTTKNVSVGIVGKDMEFTIYDDGDVASFLEGLEERP
QRRVAQPDDEPAAEKPEEPMEL
>PSMA1.3 XM_014175998.1 XP_014031473.1 ssa26 NC_027325.1:27.532.592-27.537.182
MFRNQYDNDVTVWSPQGRIHQIEYAMEAVKQGSATVGLKSRGHAVLVALKRAQSELAAHQ
KKILHVDSHIGISIAGLTADARLLCNFMRQECLDSRFVFDRPLPVSRLVTLIGSKTQIPT
QRYGRRPYGVGLLIAGYDDMGPHIFQTCPSANYFDCKAMSIGARSQSARTYLERHMDTFF
DCELNDLVQHGLRGLRETLPAEQDLTTKNVSIGIVGKDMEFTIYDDGDVASFLEGLEERP
QRRVTQPDDEAAAEKPEEPMEL
>PSMA2.1a XM_014142648.1 XP_013998121.1 ssa14 NC_027313.1:72.800.465-72.806.030
MAERGYSFSLTTFSPSGKLVQIEYALAAVAAGAPAVGIKASNGVVLATEKKQKSILYDET
SVHKVEPITKHIGMVYSGMGPDYRVLLRRARKLAQQYFLVYQEPIPTAQLVQRVASVMQE
YTQSGGVRPFGVSLLIAGWDEDRPYLFQSDPSGAYFAWKATAMGKNYVNGKTFLEKRYNE
DLELEDAIHTAILTLKESFEGQMTEDNIEVGICNEAGFRRLSPAEVKDYLAAIA
>PSMA2.1b XM_014178626.1 XP_014034101.1 ssa27 NC_027326.1:35.121.206-35.126.036
MAERGYSFSLTTFSPSGKLVQIEYALAAVAAGAPAVGIKASNGVVLATEKKQKSILYDET
SVHKVEPITKHIGMVYSGMGPDYRVLLRRARKLAQQYFLVYQEPIPTAQLVQRVASVMQE
YTQSGGVRPFGVSLLIAGWDEDRPYLFQSDPSGAYFAWKATAMGKNYVNGKTFLEKRYNE
DLELEDAIHTAILTLKESFEGQMTEDNIEVGICNEAGFRRLSSAEVKDYLAAIA
>PSMA2.2 XM_014186627.1 XP_014042102.1 NW_012355036.1:2508-7365
MGERGYSFSLTTFSPSGKLVQIEYALSAVAAGAPSVGIKASNGVVLATEKKQKSILYDET
SVHKVEMITKHIGMVYSGMGPDYRVLLRRARKLAQQYFLVYQEPIPTAQLVQRVASVMQE
YTQSGGVRPFGVSLLIAGWDDDHPYLFQSDPSGAYFAWKATAMGKNYVNGKTFLEKRYNV
DLELEDAIHTAILTLKESFEGQMTEDNIEVGICNEAGFRRLTTAEVKDYLAAIA
>PSMA2.3 XM_014186794.1 XP_014042269.1 NW_012355663.1:2532-6999
MGERGYSFSLTTFSPSGKLVQIEYALSAVAAGAPSVGIKASNGVVLATEKKQKSILYDET
SVHKVEMITKHIGMVYSGMGPDYRVLLRRARKLAQQYFLVYQEPIPTAQLVQRVASVMQE
YTQSGGVRPFGVSLLIAGWDDDHPYLFQSDPSGAYFAWKATAMGKNYVNGKTFLEKRYNV
DLELEDAIHTAILTLKESFEGQMTEDNIEVGICNEAGFRRLTTAEVKDYLAAIA
>PSMA2.4 XM_014188636.1 XP_014044111.1 NW_012365643.1:3019-4883
MGERGYSFSLTTFSPSGKLVQIEYALSAVAAGAPSVGIKASNGVVLATEKKQKSILYDET
SVHKVEMITKHIGMVYSGMGPDYRVLLRRARKLAQQYFLVYQEPIPTAQLVQRVASVMQE
YTQSGGVRPFGVSLLIAGWDDDHPYLFQSDPSGAYFAWKATAMGKNYVNGKTFLEKRYNV
DLELEDAIHTAILTLKESFEGQMTEDNIEVGICNEAGFRRLTTAEVKDYLAAIA
>PSMA2.5 XM_014186964.1 XP_014042439.1 NW_012356382.1:225-2340
MGERGYSFSLTTFSPSGKLVQIEYALSAVAAGAPSVGIKASNGVVLATEKKQKSILYDET
SVHKVEMITKHIGMVYSGMGPDYRVLLRRARKLAQQYFLVYQEPIPTAQLVQRVASVMQE
YTQSGSVLFCGFPQV
>PSMA2.6 XM_014186967.1 XP_014042442.1 NW_012356383.1:225-2340
MGERGYSFSLTTFSPSGKLVQIEYALSAVAAGAPSVGIKASNGVVLATEKKQKSILYDET
SVHKVEMITKHIGMVYSGMGPDYRVLLRRARKLAQQYFLVYQEPIPTAQLVQRVASVMQE
YTQSGSVLFCGFPQV
>PSMA3a XM_014211237.1 XP_014066712.1 ssa09 NC_027308.1:34.657.266-34.663.160
MSSIGTGYDLSASTFSPDGRVFQVEYAMKAVENSSTAIGIRCKDGVVFGVEKLVLSKLYE
QGSNKRIFNVDRHVGMAVAGLLADARSLSEVAREEASNFRSNYGHNIPLKHLSERVAMYV
HAYTLYSAVRPFGCSFILGSYDQDDGPQLYMVDPSGISYGYWGCAIGKAKQAAKTEIEKL
QMKDMTCRELVKEVAKIIYIVHDEVKDKSFELELSWVGEVTNGKHELVPKDVREEAEKYA
KDSLEEEDDSDEDNM
>PSMA3b XM_014194707.1 XP_014050182.1 ssa01 NC_027300.1:35.732.419-35.746.877
MSSIGTGYDLSASTFSPDGRVFQVEYAMKAVENSSTAIGIRCKDGVVFGVEKLVLSKLYE
QGSNKRIFNVDRHVGMAVAGLLADARSLSEVAREEASNFRSNYGHNIPLKYLSERVAMYV
HAYTLYSAVRPFGCSFILGSYDQDDGPQLYMVDPSGISFGYWGCAIGKAKQAAKTEIEKL
QMKDMTCRELVKEVAKIIYIVHDEVKDKAFELELSWVGEVTNGKHELVPKDIRVEAEKYA
KDSLEEEDDSEEDNM

>PSMA4.1a NM_001141043.1 NP_001134515.1 ssa26 NC_027325.1:32.939.278-32.943.243
MSRRYDSRTTIFSPEGRLYQVEYAMEAIGHAGSCLGILANDGVLLAAERRNIHKLLDEVF
FSEKIYKLNEDMACSVAGITSDANVLTNELRLIAQRYLLQYQEPIPCEQLVTALCDIKQA
YTQFGGKRPFGVSLLYMGWDKHYGFQLYQSDPSGNYGGWKATCIGNNSAAAVSMLKQDFK
EGEMSLSSALALAVKVLNKTMDVSKLSAEKVEIATLTREDGKTKIKVLKQKDVEELIKRH
EAEEAKAEKDKKDKEQKEKDK
>PSMA4.1b XM_014127632.1 XP_013983107.1 ssa11 NC_027310.1:38.380.553-38.384.534
MSRRYDSRTTIFSPEGRLYQVEYAMEAIGHAGSCLGILANDGVLLAAERRNIHKLLDEVF
FSEKIYKLNEDMACSVAGITSDANVLTNELRLIAQRYLLQYQEPIPCEQLVTALCDIKQA
YTQFGGKRPFGVSLLYMGWDKHYGFQLYQSDPSGNYGGWKATCIGNNSAAAVSMLKQDFK
EGEMSLSSALALAVKVLNKTMDVSKLSAEKVEIATLTREDGKTKIKVLKQKEVEELIKRH
EAEEAKAEKDKKDKEQKEKDK
>PSMA4.2 XM_014124038.1 XP_013979513.1 ssa10 NC_027309.1:62.944.801-62.979.504
MSRRYDSRTTIFSPEGRLYQVEYAMEAIGHAGTCLGILANDGVLLAAERRNIHKLLDEVF
FSEKIYKLNEDMACSVAGITSDANVLTNELRLIAQRYLLQYQEPIPCEQLVTALCDIKQA
YTQFGGKRPFGVSLLYMGWDKHYGFQLYQSDPSGNYGGWKATCIGNNSAAAVSMLKQDFK
EGEMSLSSALALAVKVLNKTMDVSKLSAEKVEIATLTRENGKTCIKVLKQKEVDELIKKH
EAEEAKAEKEKKEKEQKEKDK
>PSMA4.3 XM_014188166.1 XP_014043641.1 NW_012362606.1:5387-6061
ANVLTNELRLIAQRYLLQYQEPIPCEQLVTALCDIKQAYTQFGGKRPFGVSLLYMGWDKH
YGFQLYQSDPSGNYGGWKATCIGNNSAVSLFAVINIGTMHMFWQQHCHK
>PSMA5 NM_001140960.1 NP_001134432.1 ssa22 NC_027321.1:25.311.965-25.316.615
MFLTRSEYDRGVNTFSPEGRLFQVEYAIEAIKLGSTAIGIQTSEGVCLAVEKRITSPLME
PSSIEKIVEIDTHIGCAMSGLIADAKTLIDKARVETQNHWFTYNETMTVESVTQAVSNLA
LQFGEEDADPGAMSRPFGVALLFGGLDEKGPQLYHMDPSGTFVQCDARAIGSASEGAQSS
LQEVYHKSMTLKEAIKSSLTILKQVMEEKLNATNIELATIEPGKTFHMYSKEELEDVIKD
I
>PSMA6.1a XM_014204688.1 XP_014060163.1 ssa06 NC_027305.1:48.395.48348.398.517
MSRGSSAGFDRHITIFSPEGRLYQVEYAFKAINQGGLTSVAVRGKDCAVVVTQKKVPDKL
LDASTVTHLFRITENIGCVMSGMTADSKSQVQRARYEAANWKYKYGYEIPVDMLCKRIAD
ISQVYTQNAEMRPLGCCMIVIGVDEECGPQVYKCDPAGYYCGFKATAAGVKQTEATSFLE
KKVKKKLDWTFKETVETAITCLSTVLSIDFKPSELEIGVITTEDPKFRILTESEVDIHLV
SLAERD
>PSMA6.1b XM_014144015.1 XP_013999490.1 ssa15 NC_027314.1:24.839.460-24.842.313
MSRGSSAGFDRHITIFSPEGRLYQVEYAFKAINQGGLTSVAVRGKDCAVVVTQKKVPDTL
LDSSTVTHLFRITENIGCVMSGMTADGKSQVQRARYEAANWNYKYGYEIPVDMLCKRIAD
ISQVYTQNAEMRPLGCCMIVIGVDDECGPQVYKCDPAGYYCGFKATAAGVKQTEATSFLE
KKVKKKLDLTFKETVETAITCLSTVLSIDFKPSELEIGVISTEDPKFRILTESEVDTHLV
SLAERD
>PSMA6.2a XM_014213952.1 ssa09 NC_027308.1:105.413.273-105.421.306
MSRGSNAGFDRHITIFSPEGRLYQVEYAFKAIAQGGLTSLGVRGTDCAVVVTQKKVPDKL
LDSATLTNMYPLTPRIGCVMTGHNADSRSQVHRARVEAAEWKYKFGYDITADMLCRRMAD
ISQVYTQNAEMRPLGCCMILVAMDPQLGPMLYKCDPAGYFCGFRATSVGAKQTEANSYLE
KKLKKRPELSYEMTVELAISCLSSILSMDFKPSEIEVGVVTKESPKFRTLSETEIDTHLV
AIAERE
>PSMA6.2b XM_014162314.1 XP_014017788.1 ssa20 NC_027319.1:49.538.936-49.542.614
MSRGSNAGFDRHITIFSPEGRLYQVEYAFKAIAQGGLTSLGVRGTDCAVVVTQKKVPDKL
LDSATLTNMYPLTPRIGCVMTGHNADSRSQVHRARVEAAEWKYKFGYDITADMLCRRMAD
ISQVYTQNAEMRPLGCCMIFVAIDPQLGPVLYKCDPAGYFCGFRATSVGAKQTEANSYLE
KKLKKRPELSYEMTVELAISCLSSILSMDFKPSEIEVGVVTKESPKFRTLSETEIDTHLV
AIAERE
>PSMA6.3 XM_014211118.1 XP_014066593.1 ssa09 NC_027308.1:31.342.847-31.358.760
MSRGSSAGFDRHITIFSPEGRLYQVEYAFKAINQGGLTSLAIRGQDCAVVVTQKKVPDKL
LDASTVTHLFKITENIGCVMSGMTADSKSQVQRARYEAANWKYKYGYEIPVDMLCKRMAD
ISQVYTQNAEMRPLGCCMIVIGVDEECGPQVYKCDPAGYYCGFKATAAGVKQTEATTFLE
KKVKKKLDWTFAQTIETAITCLSTVLSIDFKPSELEIGVITTEDPKFRILTETEIDVHLV
SLSERD
>PSMA7a XM_014145490.1 XP_014000965.1 ssa15 NC_027314.1:60.419.493-60.421.676
MSYDRAITVFSPDGHLFQVEYAQEAVKKGSTAVGVRGKNVVVLGVEKKTVAKLQDDRTVR
KICALDDNVFMAFAGLTADARIVVNRARVECQSHRLTVEDPVTVEYITRFISSIKQRYTQ
SNGRRPFGISSLIVGFDFDGTPHLYQTDPSGTYHAWKANAIGRSAKTVREFLEKNYKEEH
MESDTDTIKLAIRALLEVVQSGGKNIELAIMKRDESMKILVQEEIEEYVTAIEKEKEEAE
KQKKKT
>PSMA7b XM_014135081.1 XP_013990556.1 ssa13 NC_027312.1:19.630.202-19.635.045

MSYDRAITVFSPDGHLFQVDYAQEAVKKGSTAVGVRGKNIVVLGVEKKTVAKLQEDRTVR
KICSLDDNVFMAFAGLTADARIIVNRARVECQSHRLTVEDPATVEYITRYISSIKQRYTQ
SNGRRPFGISSLIVGFDFDGTPHLYQTDPSGTYHAWKANAIGRSAKTVREFLEKNYKEDM
ESDTDTIKLAIRALLEVVQSGGKNIELAIMKRDESMKILVQEEIEEYVTAIETEKAEKQK
KP
>PSMA8.1a XM_014169175.1 XP_014024650.1 ssa23 NC_027322.1:14.663.367-14.665.838
MAARYDRAITVFSPDGHLFQVEYAQEAVKKGSTAVGIRGKDIVVLGVEKKSVAKLQEERT
VRKICALDDHVCMAFAGLTADARIVINRARVECQSHRLTVEDPVTVEYITRHIATLKQRY
TQSNGRRPFGISALIVGFDCDGTPRLYQTDPSGTYHAWKANAIGRSAKTVREFLEKNYTE
ESIATDNEAIKLAIKALLEVVQSGGKNIELAVIRRNQSLKLLESKEIETLVTEIEKEKEE
EAEKKKQKKST
>PSMA8.1b_XM_014123186.1 XP_013978661.1 ssa10 NC_027309.1:35.937.614-35.940.144
MAARYDRAITVFSPDGHLFQVEYAQEAVKKGSTAVGIRGKDIVVLGVEKKSVAKLQEERT
VRKICALDDHVCMAFAGLTADARIVINRARVECQSHRLTVEDPVTVEYITRHIATLKQRY
TQSNGRRPFGISALIVGFDNDGTPRLYQTDPSGTYHAWKANSIGRSAKTVREFLEKNYTD
EAIATDNEAIKLAIKALLEVVQSGGKNIELAVIRRNQPLKLLESKEIETLVAEIEKEKEE
EAEKKKPKKST
>PSMA8.2a XM_014140233.1 XP_013995708.1 ssa14 NC_027313.1:27.557.885-27.578.124
MAARYDRAITVFSPDGHLFQVEYAQEAVKKGSTAVGIRGKDIVVLGVEKKSVAKLQEERT
VCKICTLDDHVCMAFAGLTADARIVINRARVECQSHRLTVEDPVTVEYITRHIATLKQRY
TQSNGRRPFGISALIVGFDCDGTPRLYQTDPSGTYHAWKANAIGRSAKTVREFLEKNYTD
EAIATDNESIKLAIKALLEVVQSGGKNIELAVIRRNQPLKLLESKEIETLVTEMEKEKED
EAEKKKQKKST
>PSMA8.2b XM_014190989.1 XP_014046464.1 ssa03 NC_027302.1:27.740.137-27.753.767
MAARYDRAITVFSPDGHLFQVEYAQEAVKKGSTAVGIRGKDIVVLGVEKKSVAKLQEERT
VRKICALDDHVCMAFAGLTADARIVINRARVECQSHRLTVEDPVTVEYITRHIATLKQRY
TQSNGRRPFGISALIVGFDYDGTPRLYQTDPSGTYHAWKANAIGRSAKTVREFLEKNYTE
EAIATDNEAIKLAIKALLEVVQSGGKNIDLAVIRRNQPLKLLESKEIETLVAEIEKEKED
EAEKKKQKKST
>PSMB1a XM_014179894.1 XP_014035369.1 ssa28 NC_027327.1:19.352.01919.354.409
MFATQVYGDNGKMKEYHYTGPVEHRFSPYSFNGGTVLAVAGEDFAVVASDTRLSEGYSIH
SRDSPKCYKLTDTTVIGCSGFHGDCLTLTKIIDARLKMYKHSNNKCMTSGAIAAMLSTIL
YGRRFFPYYVYNIIGGLDEEGKGAVYSFDPVGSYQRDTYKAGGSASAMLQPLLDNQIGFK
NMEGVQHLPLSQEKAVQLVKDVFISAAERDVYTGDALRICIVTKDGIKEETFPLRKD
>PSMB1b XM_014210205.1 XP_014065680.1 ssa01 NC_027300.1:81.642.266-81.644.471
MFTTQVYGDNGKMKEYHYTGPVEHRFSPYSFNGGTVLAVAGEDFAIVASDTRLSEGYSIH
SRDSPKCYKLTNTTVIGCSGFHGDCLTLTKIIDARLKMYKHSNNKSMTSGAIAAMLSTIL
YGRRFFPYYVYNIIGGLDEEGKGAVYSFDPVGSYQRDTYKAGGSASAMLQPLLDNQIGFK
NMEGVQHLPLNQEKAVQLVKDVFISAAERDVYTGDALRICIVTKEGIKEETVPLRKD
>PSMB2a XM_014177580.1 XP_014033055.1 ssa27 NC_027326.1:7.633.585-7.647.942
MEYLIGIQGQDFVLVAADNVAAHSIVKMKQDQDKMFKLSDKILLLCVGEAGDTVQFAEYI
QKNIQLYKMRNGYELSPTAAANFTRKNLADYLRSRTPYHVNLLLAGFDETDGPGLYYMDH
LSALAKAPFAAHGHGAYLTLSILDRYYRPDLTRDEAVDLLKKCVEELNYRFILNLPSFSV
RLIDKDGIHDLEKLIPVGTK
>PSMB2b XM_014141946.1 XP_013997421.1 ssa14 NC_027313.1:56.740.677-56.749.913
MEYLIGIQGQDFVLVAADNIAANSIIQMKQDQDKMFKLSDKILLLCVGEAGDTVQFAEYI
QKNIQLYKMRNGYELSPKAAANFTRKNLADYLRSRTPYHVNLLLAGFDETDGPGLYYMDH
LSALAKAPFAAHGYGAYLTLSILDRYYRPDLTRDEAVGLLKKCVEELNKRFILNLPSFSV
RLIDTEGIHDLEKLMPVGAKFVARAPSS
>PSMB3a XM_014176616.1 XP_014032091.1 ssa02 NC_027301.1:46.073.424-46.075.407
MSIMSYNGGAVMAMKGKQCVAIAADRRFGVQAQMVTTDFQKIFPMGDRLYIGLAGLATDV
QTVSQRLKFRLNLYELKEGRQIKPKTFMSMVSNLLYERRFGPYYIEPVIAGLDPKTFEPF
ICSLDLIGCPMVTEDFVVSGTCSEQMYGMCESLWEPDMEPEDLFETISQAMLNAVDRDAV
SGMGVVVQVIEKDKITTRTLKARMD
>PSMB3b XM_014131496.1 XP_013986971.1 ssa12 NC_027311.1:23.091.646-23.093.585
MSIMSYNGGAVMAMKGKQCVAIAADRRFGVQAQMVTTDFQKIFPMGDRLYIGLAGLATDV
QTVSQRLKFRLNLYELKEGRQIKPKTFMSMVSNLLYERRFGPYYIEPVIAGLDPKTFEPF
ICSLDLIGCPMVTEDFVVSGTCSEQMYGMCESLWEPDMEPEDLFETISQAMLNAVDRDAV
SGMGVIVQVIEKDKITTRTLKARMD
>PSMB4a XM_014177216.1 XP_014032691.1 ssa27 NC_027326.1:11.840.919-11.845.141
MDPSGLKLNFWENGPKPGQFYSFPGSSLAPGCGPIKHTLNPMVTGTSVLGVKFTGGVIIA
ADMLGSYGSLARFRNISRLMKVNDTTILGASGDYADYQYMKQIIEQMVIDEELLGDGHSY
SPKAIHSWLTRVMYNRRSKMNPLWNTVVVGGFYNGESFLGYVDKLGVAYEAPTVATGFGA
YLAQPLMREVVENKVEITKDEARALIDRCLKVLYYRDARSYNRHEIAIVTEEGVEIVGPL

SCETNWEIAHMVSGFE
>PSMB4b XM_014141626.1 XP_013997101.1 ssa14 NC_027313.1:60.842.907-60.847.693
MDPSGLKLNFWENGPKPGQFYSFPGSSLTPGCGPIKHTLNPMVTGTSVLGVKFTGGVIIA
ADMLGSYGSLARFRNISRLMKVNDSTILGASGDYADYQYMKQIIEQMVIDEELLGDGHSY
SPKAIHSWLTRVMYNRRSKMNPLWNTVVIGGFYNDESFLGYVDKLGVAYEAPTVATGFGA
YLAQPLMREVVENKVEITKDEARALIERCLKVLYYRDARSYNRHEIAIVTKEGVEIVGPM
SCETNWEIAHMVSGFE
>PSMB5.1a XM_014169252.1 XP_014024727.1 ssa23 NC_027322.1:21.860.398-21.864.523
MALANVLQSELVDFSNYGRKRRGFASGLYETELGLGSVGGDSLSFAVRTACSDGDGPERK
IEFLHGTTTLAFKFQHGVIVAVDSRATAGAYIASQTVKKVIEINPYLLGTMAGGAADCSF
WERLLARQCRVYELRNKERISVAAASKLLANMVYQYKGMGLSMGTMVCGWDKTGPGLYYV
DSEGNRVCGDLFAVGSGSMYAYGIVDSGLKQKDLTVEEACDLGRRAIYQATYRDAYSGGQ
VNLYHVHSEGWNRVSQSDVLMLHQQYQAEKA
>PSMB5.1b XM_014122889.1 XP_013978364.1 ssa10 NC_027309.1:29.742.933-29.747.129
MALASVLQSESVDFSNYGRQRRGFASGLYEAELGLESVGGDLSFALRTLCSNGDGPDRKI
EFLHGTTTLAFKFQHGVIVAVDSRATAGSYIASQTVKKVIEINPYLLGTMAGGAADCSFW
ERLLARQCRVYELRNKERISVAAASKLLANMVYQYKGMGLSMGTMVCGWDKTGPGLYYVD
SEGNRVCGDLFAVGSGSMYAYGIVDSGLKQKDLTVEEACDLGRRAIYQATYRDAYSGGQV
NLYHVHSEGWTRVSQSDVLMLHQQYQAEKA
>PSMB5.2 XM_014140774.1 XP_013996249.1 ssa14 NC_027313.1:34.940.728-34.942.524
MALSSVLQGESADFSFSNDRAFSFGCGFGQTDLGFGAAPGDRLNFAIKASLGPDDKDGPE
RKIEFLHGTTTLAFKFQHGVIVAVDSRATAGAYIASQTVKKVIEINPYLLGTMAGGAADC
SFWERLLARQCRVYELRNKERISVAAASKLLANMVYQYKGMGLSMGTMVCGWDKRGPGLY
YVDSEGNRVCGDLFAVGSGSMYAYGVVDSGLRQDLSVEEACELGRRAIYQATYRDAYSGG
QVNLYHVHSEGWTRVSQEDVLKLHHQYQEPKK
>PSMB6a XM_014128982.1 XP_013984457.1 ssa11 NC_027310.1:77.336.790-77.339.742
MATAYMSANRSENAFSTADLVPEWAQEEVSTGTTIMAVEFDGGVVIGADSRTTTGAYIAN
RVTDKLTPIHDRIFCCRSGSAADTQAVADIVTYQLGFHSIELDEPPLVQTAANLFKQTCY
RYREELMAGIIVAGWDKRRGGQVYTVPMGGMIVRQPVSVGGSGSSYIYGFMDSNYKPGMT
KEECLHFCTQALALAMERDGSSGGVARLAAITEEGLERRVVLGNQLPKFSNA
>PSMB6b XM_014196309.1 XP_014051784.1 ssa04 NC_027303.1:35.985.565-35.988.288
MAAYMSASHSENAFSTHDFVPEWAQEEVSTGTTIMAVEFDGGVVIAADSRTTTGAYIANR
VTDKLTPIHDHIFCCRSGSAADTQAVADIVTYQLGFHSIELDEPPLVQTAANLFKQTCYR
YREELMAGIIVAGWDKRRGGQVYTVPMGGMIVRQPVSVGGSGSSYIYGFMDSNYKPGMTK
EECLHFCTQALALAMERDGSSGGVARLAAITEEGLERRVVLGNQLPRFSNA
>PSMB7 XM_014143540.1 XP_013999015.1 ssa01 NC_027300.1:147.075.032-147.096.867
MATLSVCQPQLGGFSFENCKRNAVLEAEVTKLGCNIPAARKTGTTICGVVFKDGLVLGAD
TRATEGMIVADKNCSKIHYISPNIYCCGAGTAADTEMTTQIISSNLELHSLSTSRLPRVA
TANRMLKQMLFRYQGHIGAALVLGGVDCNGPHLYSIYPHGSTDKLPYVTMGSGSLAAMAV
FEDRYKQDMEEEDAKRLVRDAIAAGIFNDLGSGSNIDLCVITKGRVDYLRPHDMANKKGV
RCVYEYEALPHLTGNYKYKQGTTGVLTKSVIKLDLEVVEETVQTMDTS
>PSMB8a_#C XP_014032817.1 NC_027326:10.153.897-10.157.743
MALFDVSGYKSYSELRGQIIGTGVGHFIDRPNKQFSVPVGVDPSGFLKSCSREGGVSIDL
NHGTTTLAFTFRHGVIVAVDSRASAGSYIASKEANKVIEINPYLLGTMSGSAADCQYWER
LLAKECRLYKLRNKQRISVSAASKLLCNMMLGYRGMGLSMGSMIVGWDNKGPGLYYVDDN
ATRLSGRMFSTGCGSSYAYGVIDSGYREDMTVEEAYELGRRGITHATHRDAYSGGVVNLY
HMQEDGWIKVCKEDVSELIHRYRKGMF
>PSMB8b_#C NP_001117007.1 NC_027313:59.095.880-59.098.288
MALFDVSGYKSHAGLRGQILGTGVGHLVDRPNQEFAVPVGVDPSGFLKSCSREGGVSIDL
NHGTTTLAFTFRHGVIVAVDSRASAGSYIASKEANKVIEINPYLLGTMSGSAADCQYWER
LLAKECRLYKLRNKQRISVSAASKLLCNMMLGYRGMGLSMGSMIIGWDNKGPGLYYVDDN
ATRLSGRMFSTGCGSSYAYGVVDSGYREDMTVEEAYELGRRGITHATHRDAYSGGVVNLY
HMQEDGWIKVCKEDVSELIHRYRKGMF
>PSMB9a_#C XP_014032548.1 NC_027326:10.171.104-10.175.148
MLEESSEPGWLSEEVKTGTTIIAIEFDGGVVLGSDSRVSAGETVVNRVMNKLSLLHDKIY
CALSGSAADAQTIAEMVNYQLDVHSIEVGEDPQVRSAATLVKNISYKYKEELSAHLIVAG
WDKRGGGQVYVTLNGLLSRQPFAVGGSGSAYVYGFVDAEYRKAMSKEDCQQFVVNTLSLA
MSRDGSSGGVAYLVTIDEKGAEEKCILGNELPTFYDQ
>PSMB9b_#C NP_001117186.1 NC_027313:59.112.113-59.116.449
MLEESSEPGWLSEEVKTGTTIIAIEFDGGVVLGSDSRVSAGETVVNRVMNKLSLLHDKIY
CALSGSAADAQTIAEMVNYQLDVHSIEVGEDPQVRSAATLVKNISYKYKEELSAHLIVAG
WDKRGGGQVYVTLNGLLSRQPFAVGGSGSAYVYGFVDAEYRKAMSKEDCQQFVVNTLSLA
MSRDGSSGGVAYLVTIDEKGAEEKCILGNELPTFYDQ
>PSMB10a_#C XP_014032774.1 NC_027326:10.580.777-10.585.553

MLHNSRPPQPQSAGFSFENTRRNAVLEGNLSELGYSSPKARKTGTTIAGIVFKDGVILGA
DTRATDDMVVADKNCMKIHYIAPNIYCCGAGVAADAEVTTQMMSSNVELHSLSTGRPPLV
VMVTRQLKQMLFRYQGHIGSSLIVGGVDVTGAHLYSVYPHGSYDKLPFLTMGSGAGAAIS
IFEDRYRPNMELEEAKKLVRDAIAAGIFCDLGSGSNVDLCVITQAGVQYLRSYDQPAQKG
KKEGQYKYKPGTTAVLTKTVTPLPLDVVDESIQLMDAQ
>PSMB10b_#C XP_013997188.1 NC_027313.1: 59.678.647-59.684.589
MLNNSRPYQPQSAGFSFENTRRNAVLEGNLSELGYSSPKARKTGTTIAGIVFKDGVILGA
DTRATDDMVVADKNCMKIHYIAPNICCGAGVAADAEVTTQMMSSNVELHSLSTGRPPLVV
TVTRQLKQMLFRYQGHIGSSLIVGGVDVTGAHLYSVYPHGSYDKLPFLTMGSGAGAAISI
FEDRYRPNMELEEAKKLVRDAIAAGIFCDLGSGSNVDLCVITQAGVQYLRSYDQPAQKGK
KEGQYKYKPGTTAVLTKTVTPLPLHVVDESIQLMDTQ
>PSMB11.1 XM_014123190.1 XP_013978665.1 ssa10 NC_027309.1:29.754.010-29.756.117
MALQDLCGFKDSFLNSRWNFQASHRMAQEDTCMLFGKRTRDISSRGLSWSNSESPLHLYI
PAAEYLSESPIQFGQINPNTAPNHITPLHQDSNLPFLSFPPGFPPSSSPSVPLPFPMSHG
TTTLAFMFQGGVIAAADTRSSCNGLVACPASQKILPVHSHLVGTTSGTSADCALWKRILA
RELRLYQLRHGRRLSTAGAAKLLAHMLHPFKGTELCVAATLCGWDGGEVETGPLEHGSKG
ADQQENRSKMGSETDDLEKTSESDSISQAEARGRNPCLSPAMSRQHCSAGGKQLVLSGPS
LYYVCSDGTRLQGALFSVGSGSPYAYSVLDGGVQWGLTVEEAISLAREAVYRATHRDAYS
GNCVDVYHITSHGWTRRDREELREEYYREKERERVKVKGRREKQEGVEDGQSSGRKK
>PSMB11.2 XM_014189126 XP_014044601.1 NW_012371889.1:3442-3942
MGSGRDDSISPVKRPLEETGMSTSDACSQDDLTDSACERTRHAVTDVDLTRSPVRDRCGP
KLFYVCSDGTRLQGELFSVGSGSPYAYGVLDGEMRWSLNEEEAISLAREAVYRATHRDAY
SGNCVDVFHITAQGYCRRDREDLREEYHRERERVLNRGKEESAEDKK
>PSMB12a_#C XP_014032816.1 NC_027326:10.163.427-10.168.543
MERHLMDSQIKGVSTGTTILAVTFNGGVIIGSDSRASIGGSYVSSKTINKLIQVHDRIFC
CIAGSLADAQAVTKAAKFQISFHSIQMESPPLVKAAASVLKELCYNNKVMLQAGFITAGW
DRKKGPQVYTVALGGMLLSQPFTIGGSGSTYIYGYADAKYKPDMSKEECLQFAKNALALA
MGRDNVSGGVAHLVVITEEGVEHVVIPGDKLPKFHDE
>PSMB12b_#C XP_013997223.1 NC_027313:59.103.305-59.111.118
MDSQIKGVSTGTTILAVTFNGGVIIGSDSRASIGGSYVSSKTINKLIQVHDRIFCCIAGS
LADAQAVTKAAKFQISFHSIQMESPPLVKAAASVLKELCYNNKEELQAGFITAGWDRKKG
PQVYTVALGGMLLSQPFTIGGSGSTYIYGYADAKYKPDMSKEECLQFATNALALAMGRDN
VSGGVAHLVVITEEGVEHVVIPGDKLPKFHDE
>PSMB13a_#C NC_027326.1:10.158.350-10.163.366 incomplete sequence
MALSNVLEIPTSGFNFENVARNVALEGLLEGGHTKTLKPMKTGTTIAGLVCKEGVVLGAD
TRATSGEVVADKMCAKIHYISPNISCCGAGTAADTEKTTDLLSSNLTIFSMNSGRNPRVV
MAVRYRGQIGASLILGGVDCTGNHLYTVGPYGSIDNVQYLAMGMEEAKELVRDAIHSGIM
SDLGSGNNIDICVITKQGVDYIRPYQESEGTGTTSILTEKIVPLELEVVQKTVQRMDTA
>PSMB13b_#C XP_013997221.1 NC_027313:59.099.410-59.103.327
MALTNVVETPASGFNFENVSRNVALEGLLEGGHTKAPKPMKTGTTIAGVVCKDGVVLGAD
TRATSGEVVADKMCAKIHYISPNMYCCGAGTAADTEKTTDMLSSNLTIFSMTSGRNPRVV
MAVNILQDMLFRYRGQIGASLILGGVDCTGNHLYTVGPYGSIDNVPYLAMGSGDLAALGI
LEDRFKPNMELEEAKELVRDAIHSGIMSDLGSGNNIDICVITKQGVDYIRPYQESEYKDK
RQRRYKYRPGTTSILTEKIVPLELEVVQETVQRMDTA
>PSME1a XM_014181079.1 XP_014036554.1 ssa29 NC_027328.1:20.102.086-20.115.998
MTSIDIRPESKKMVDDFCTRLTKEAETLVTSFFPQKIAEMEMLLKKSFSTDGLAALKSPL
DIPMPDPAKEEAKRKKKEEKEAKEGKKEKDSDKEDEDAGPACGPIPCNERVESLLKEIKP
QIQILKEKLNTVSMWVQLQIPKIEDGNNFGVAVQEKVFELLTNTRTKIEGFQTQISKYYS
ERGDAVDKASKEPHVGDYRQLVHELDQYQYCELRIVVLEIRNTYAVLLDIINKNYDKIKK
PRGDCKALIY
>PSME1b XM_014159707.1 XP_014015182.1 ssa19 NC_027318.1:74.400.001-74.401.958
MTSIDIRPESKKQVDDFCTQLTKEAETLVTSFFPQKIAEMDMLLKTSLSTEGLAALKAPL
DIPIPDPAKEEAKQKKKEEKEAKEGKKDKDSEKEDEDAGPPCGPIPCNERVESLLKEIKP
QIQLLKEKLNTVSMWVQLQIPKIEDGNNFGVAVQEKVFELLTNTRTKIEEFQTQISKYYS
ERGDAVAKASKQPHVGDYRQLVHELDQYQYCELRIVVLEIRNTYALLFDIINKNYDKIKK
PRGDCKALIY
>PSME2a NM_001141654.1 NP_001135126.1 ssa29 NC_027328.1:17.457.754-17.464.706
MSRSSVLKIKSANAVKVENFRQSLYQQAEDLFSNYIPLKITQLDNLLKEEDLSITDLSTL
HAPLDIPIPDPPTPEDEEMETDKNDDDEKKKKAPKCGFIKGNEKIVKLLDRVKPEILALR
ETIITVSCWIQHLIPKIEDGNDFGVAIQEKILERIVAVKTKVDGFHTNINKYFSERGDAV
SKASKLTHVMDYRSLVHEKDEAVYSDIRVILLDIRGFYAELYDIISKNLEKVTNPKGEEK
PSMY
>PSME2b XM_014159848.1 XP_014015323.1 ssa19 NC_027318.1:77.929.617-77.936.890
MSKSSVMKIKSVNAVKVEHFHHSLYQQADDLFSNYIPLKISQLDNLLKEDDLNIPDLSTL

QAPLDIPIPDPPTAEDEEMETDKNDDKKKKKKAPSCGLIKGNEKIVKLLDRVKPEILSLR
ETIITVSCWIQHLIPKIEDGNDFGVAIQEKILERIAAVKTKVDGFHTNINKYFSERGDAV
AKASKSTHVMDYRSLVHEKDEAVYSDIRVILLDIRGFYVELYDIISKNLEKVTNPKGEEK
PSMY
>PSME3.1a XM_014193350.1 XP_014048825.1 ssa03 NC_027302.1:66.606.224-66.611.859
MSSKGIKVDNDLKTKVDAFRERITGEAENLVADFFPKKLLELDSFLKEPILNVADLKEIH
SEINVKVPDPIILNNSHDGVDVQNSRKRKMEDGLEDDNCQDGPKVFAMPGGMIKSNGQLV
DLIERVKPEIRTLIEKCNTVKMWVQLLIPRIEDGNNFGVSIQEETVAELRTVEGEAASYL
DQISRYYITRAKLVSKITKYPHVEDYQRTVTEIDEKEYISLKIIVSELRNQYVTLHDMIL
KNIEKIKRPRSSNNEALY
>PSME3.1b XM_014203891.1 XP_014059366.1 ssa06 NC_027305.1:32.643.045-32.652.898
MSSKGIKVDNDLKTKVDAFRERITGEAENLVADFFPKKLLELDSFLKEPILNVADLKEIH
SEINIKVPDPIILNNSHDGVDVQNSRKRKMEDGLDDDNCQDGPKVFAMPGGMIKSNGQLV
DLIERVKPEIRTLIEKCNTVKMWVQLLIPRIEDGNNFGVSIQEETVAELRTVEGEAASYL
DQISRYYITRAKLVSKITKYPHVEDYQRTVTEIDEKEYISLKIIVSELRNQYVTLHDMIL
KNIEKIKRPRSSNNEALY
>PSME3.2a XM_014158504.1 XP_014013979.1 ssa19 NC_027318.1:49.740.013-49.746.327
MNSLLKLDNELNIKVDAFRQRITGEAENLVASFFPNKLLELDHFLKEIHSEINLTVPDPI
LLSNLHGGLEAQNAKKRKMEDGTGEDKVAGTKVFIMPSGMMKSNAKLVDLIEKVKPEIRT
LIEKCNTVKMWVQLLIPRIEDGNNFGVSIQEETVAELRTVEGEAASYLDQISGYYITRAK
LVSKIAKYPHVEDYRRTVTEIDEKKYISLKVIVSELRNQYVTLHDMILKNIDKIKKPRSS
NAEALY
>PSME3.2b XM_014179106.1 XP_014034581.1 ssa28 NC_027327.1:2.594.610-2.600.798
MNSLLKVDNELKTKVDAFRERITGEAEELVASFFPNKLLELDHFLKDPGLNICELKEIHS
EINLTVPDPIILSNLHDGLEAQNAKKRKMEDGSGEDKVAGTKVFVMPGGMMKSNAKLVDL
IEKVKPEIRTLIEKCNTVKMWVQLLIPRIEDGNNFGVSIQEETVAELRTVEGEAASYLDQ
ISRYYITRAKLVSKIAKYPHVEDYRRTVTEIDEKEYISLKIIVSELRNQYVTLHDMILKN
IDKIKKPRSSNAEALY
>PSME3.3 XM_014189387.1 XP_014044862.1 NW_012376700: 1.963-3.779
ELRTVEGEAASYLDQISRYYITRAKLVSKITKYPHVEDYQRTVTEIDEKEYISLKIIVSE
LRNQYVTLHDMILKNIEKIKRPRSSNNEALY

SF5.13 Deduced MHC class II pathway gene sequences

Only a fragment of the predicted duplicate allelic MHCII beta transcript XM_014191566.1 (669 base pairs) is present on scaffold NW_012562982.1 (454 base pairs). This transcript is then a pseudogene, which has been “extended” in the automated annotation process.

>DAB*0101 XM_014133067.1 XP_013988542.1 ssa12 NC_027311.1:61.693.946-61.699.456
MSMSIFCVSLTLVLSIFSGTDGYFEQVVRQCRYSSKDLQGIEFIDSYVFNKAEYIRFNST
VGKFVGYTELGVKNAEAWNSDAAVLAVERGELERYCKHNADLHYSTILDKTVEPHVRLSS
VAPPSGRHPAMLMCSAYDFYPKPIRVTWLRDGREVKSDVTSTEELANGDWYYQIHSHLEY
TPRSGEKISCMVEHISLTEPMVYHWDPSLPEAERNKIAIGASGLVLGAILALAGLIYYKK
KSSGVL
>DAB*2302ψ XM_014191566.1 XP_014047041.1 NW_012562982.1:173-454
EPHVRLSSVAPPSGRHPAMLMCSAYDFYPKPIRVTWLRDGREVKSDVTSTEELANGDWYY
QIHSHLEYTPRSGEKISCMVEHISLTEPMVYHW
>DAA*1201 XM_014133066.1 XP_013988541.1 ssa12 NC_027311.1:61.701.374-61.703.966
NMKTSVIVLILCWQVYAEHKVLHIDLHITGCSDSDGVEMYGLDGEEMWYADFNKGEGVMP
LPPFADPFTYPGAYEQAVGNQGICKANLAVNIKAYKNPEEKIDPPHSSIYPRDDVDLGVE
NTLICHVSGFHPAPVRVRWTRNNQNLTEGVRLSTPYPNADFTLNQFSSLPFTPEEGDIYG
CTVEHKGLAEPLTRIWEPEVIQPSVGPAVFCGVGLTVGLLGVAAGTFFLIKGNQCN
>Ii.1a XM_014199898.1 XP_014055373.1 Invariant chain ssa05 NC_027304:37.021.326-37.026.612
MEGQQQHDDALLERTGSQDVILPMTATRGASNSRPLKIAGFTVLACLLLAGQAFTAYMVF
NQRGQIHDMEKSNDNMRKQLRNRPPVAPVQMHMPMLNMPRLIDFTDEDKKTPMTKLEATA
IVSLEKQVKDLLQNPQLPQFNETFLANLQSLKRQMEEAEWKGFEAWTRNWLLFQMAQEKP
PATTPQPAAGLQTKCNLEASKGRKLGAYLPQCDEQGNYLPMQCWHATGFCWCVDKDGKPI
EGTSIRGRATCDRFPSRMAAFPRMMQLKEYKDE
>Ii.1b XM_014212490.1 XP_014067965.1 ssa09 NC_027308.1:68.290.229-68.299.367
MEEQQQQRHDDALLERAGSQDVILPITTNTRASNSRAFKVAGLTVLACLLLAGQALTAYL
VFNQRGQIHDMQKSNDNMRKQLRNRPLAVAPVKMQMPMLNMARLIDFTDEDSKTPMTNLE
ATAIAIVSLEDQVKDLLQNPQLPQFNETFLANLQSLKQQVEETEWEGFETWVRYWLLFQM

AQEKPPAPPTPQPVPHQRMAAYPRMMQLKEYKNE
>CTSL1a1 XM_014126439.1 XP_013981914.1 swsa01 NC_027300.1:113.270.838-113.273.816
MDCSRPQGNEGCNGGLMDRAFQYVQDNGGLDTEASYPYVGKDDLCHYRPEFSAVNETGFVDIPSGKEHAL
MSAVASVGPVSVAIDGSHESFQFYQSGIYYEKECSSEQLDHGVLVVGYGFEGEDVDGKKFWIVKNSWSEK
WGDKGYVYIAKDKMNHCGIATAASYPLV
>CTSL1a2 XM_014126425.1 XP_013981900.1 ssa01 NC_027300.1:113.282.847-113.288.939
MMSLYLAVLVLCMSAVYAAPMFDSQLEGHWHLWKNWHSKNYHESEQGWRRMVWEKNLKKIEMHNLDHTMG
KHSYRLGMNHFGDMTNEEFGQLMNGYKQTTERKYKGSLFMEPSYLQAPEAVDWREKGYVTPVMDQGSCGS
CWAFSSTGAIEGQLFRKTGNLVSLSEQNLVDCSRPQGNEGCNGGLMDLAFQYVKDNGGLDTEASYPYVGK
DDLCHYRPEFSAVNETGFVDIPSGKEHALMSAVASVGPVSVAIDGSHESFQFYQSGIYYEEECSSEQLDH
GVLVVGYSFEGEDVDGKKFWIVKNSWSEKWGDKGYIYMAKDRKNHCGIATAASYPLV
>CTSL1a3 XM_014126463.1 XP_013981938.1 ssa01 NC_027300.1:113.340.522-113.345.645
MSLYLAVLVLCMSAVYAAPMFDSQLEGHWHLWKNWHSKNYHDSEEGWRRMVWEKNLKKIEMHNLDHSMGK
HSYRLGMNHFGDMTNEEFGQLMNGYKQTTERKYKGSLFMEPSYLQAPKAVDWREKGYVTPIKDQGSCGSC
WSFSSTGAIEGQEFRKTGNLVSLSEQNLVDCSRPQGNEGCNGGLMDLAFQYVKDNAGLDTEASYPYVGKD
DLCHYRPEFNAVNETGIVDIPSGNEHALMKAVASVGPVSVAIDASHNSFQFYQSGIYYEEKCSSEQLDHG
VLVVGYGFEGEDVDGKKFWIVKNSWSEKWGDKGYIYMAKDRKNHCGIATAASYPLV
>CTSL1a4 XM_014126476.1 XP_013981951.1 ssa01 NC_027300.1:113.367.197-113.372.320
MMSLYLAVLVLCMSAVYAAPMFDSQLEGHWHLWKNWHSKNYHDREEGWRRMVWEKNLKKIEMHNLDHSMG
KHSYRLGMNHFGDMTNEEFGQLMNGYKQTTERKYKGSLFMEPSYLQAPKAVDWREKGYVTPIKDQGSCGS
CWSFSSTGAIEGQEFRKTGNLVSLSEQNLVDCSRPQGNEGCNGGLMDLAFQYVKDNAGLDTEASYPYVGK
DDICHYRPEFNAVNETGFMDIPSGKEHALMKAVASVGPVAVAIDASHNSFQFYQSGIYYEEKCSSEQLDH
GVLVVGYGFEGEDVDGKKFWIVKNSWSEKWGDKGYIYIAKDRKNHCGIATAASYPLV
>CTSL1a5 XM_014126489.1 XP_013981964.1 ssa01 NC_027300.1:113.380.498-113.388.445
MSLYLAVLVLCMSALSAAPMFDSQLEGHWHLWKNLHSKNYHESEEGWRRMVWEKNLKKIEMHNLDHSMGK
HSYRLGMNHFGDMTNEEFGQLMNGYKQTTERKYKGSLFMEPSYLQAPKAVDWREKGYVTPIKNQGSCGSC
WAFSSTGAIEGQLFRKTGNLVSLSEQNLMDCSRPQGNEGCNGGLEDRAFQYVQDNGGLDTEASYPYVGKD
DYLCHYRPEFNAVNETGFVDIPSGKEHALMKAVASVGPLAVAIDASHNSFQFYQSGIYYEKECSSEQLDH
GVLVVGYGFEGEDVDAKKFWIVKNSWSEKWGDKGYIHIAKDRKNHCGIATAASYPLV
>CTSL1b NM_001146546.1 NP_001140018.1 ssa13 NC_027312.1:93.533.493-93.537.645
MTALYLAVLVLCVSAVCAAPRFDSQLEDHWHLWKNWHSKSYHESEEGWRRMVWEKNLKKIEMHNLEHTMG
KHSYRLGMNHFGDMTNEEFRQTMNGYKQTTERKFKGSLFMEPNYLQAPKAVDWREKGYVTPVKDQGSCGS
CWAFSTTGAMEGQQFRKTGKLVSLSEQNLVDCSRPEGNEGCNGGLMDQAFQYIQDNAGLDTEESYPYVGT
DEDPCHYKPEFSGANETGFVDIPSGKEHAMMKAVAAVGPVSVAIDAGHESFQFYESGIYYEKECSSEELD
HGVLVVGYGFEGEDVDGKKYWIVKNSWSEKWGDKGYIYMAKDRKNHCGIATASSYPLV
>CTSL2a XM_014124116.1 XP_013979591.1 ssa01 NC_027300.1:10.068.398-10.074.289
MKLLVVVAAALAVASAASLSLEDLEFHAWKLKFGKSYSSQVEEAQRMSSWISNRKMVVVHNMLADQGIKS
YRLGMTYFADMDNEEYRRVISQGCLGSFNTSKARGGSTFFPMLGDNDLPTTVDWRDKGYVTPIKDQKQCG
SCWAFSATGSLEGQHYKKTNKLVSLSEQQLVDCSGDFGNMGCMGGLMDQAFEYIKSLAPGGVDTEDSYPY
QAEDKKCRYKPDSVGATCSGFVDVTSGDESALQQAVATVGPVSVAIDAAHSSFQLYDSGVYDEPECSSDD
LDHGVLAVGYGTSDDGHDYWLVKNSWGLEWGDKGYIMMSRNKHNQCGIATASSYPLV
>CTSL2b XM_014210112.1 XP_014065587.1 ssa09 NC_027308.1:7.731.484-7.781.352
MVINVCFGYKYKMYGAMNKRHRCCSVMASPNTCGYLKRRMKFLVVVAAALAVASAASLSLEDLEFHAWKL
MFGKSYSSPVEESQRRSSWISNRKLVLVHNMLADQGIKSYRLGMTYFADMENEEYRRIISQRCLGSFNAS
KPRGGSTFLPMLGDNDLPTTMDWRDNGYVTSIKDQKGCGSCWAFSTTGSLEGQHYRKTNKLVSLSEQQLV
DCSGKFGNMGCMGGLMDQAFEYIKSLAPGGLDTEDSYAYHAEDKKCHYKPDSLGATCTGYVDMTSGDESA
LQQAVATVGPVSVAIDAAHSSFQLYKSGVYDEPDCSSDQLDHGVLAVGYGTSDDGQDYWLVKNSWGLDWG
DKGYIMMSRNKHNQCGIATASSYPLV
>CTSL3 XM_014131537.1 XP_013987012.1 ssa12 NC_027311.1:28.445.519-28.463.099
MQMKLLLMALCTVVSSFNQPLSTELDGIWEEWKTQHGKQYSKVDEGFRRMIWETNQGLIRQHNLEAEMGK
HTFTLGMNQFGDMTNKEYNALLTANDAEEVKSLDGIPLSKWNCSLSAAPEMWDWRLYGYVTPVKNQGSCG
SCYAFAAVGALEGQLFKQTGKLLPLSEQNLVDCSGDYHNHGCGGGLAMRCFSYVSDHGIMSERKYPYTAE
VGPCEYQNATKEAWCKGFNRVPSLDEKVFRDTLYEVGPIAVSVNATHPSFKFYKDGVLYQPDCSTRTNHA
VLAVGYGSSYLDYWIVKNSWGTGWGRDGYILMARGYNQCGIARHPVYPIM
>CTSL4 XM_014169301.1 XP_014024776.1 ssa23 NC_027322.1:28.904.769-28.907.274
MKTRQPNFPSVQWASLMGRAVLLSLLLSDPLHVASDSDEEVQEPSEWQTWKRSNGVSYDEKRDDIERKVI
WEDNKRVIDENNNSFLRGTKMFTMAMNQYGDLTRHEYKRLQGAMINSKIKKRGKNASARKLRASAQKLGS
VTVDYRAMGYVTEVKDQGYCGSCWAFSTTGAIEGQMFKRTGQLVSLSEQNLVDCSKPYGTYGCSGGAWMAN
AYNYVVQNGLQSTDTYPYTSVDTQPCFYDSSQSVASITDYRFIPSGDEQALADAVATIGPITIAVDADHP
SFMFYSSGIYEEPSCNPNNLSHAVLLVGYGSEGGHDYWIIKNSWGTAWGEGGYMRMIRNGSNTCGIASYA
LYPIL
>CTSS1a NM_001141491.1 NP_001134963.1 ssa02 NC_027301.1:23.775.018-23.782.931
MMLWSLLLAALCGIAVALFDPMLEQHWQMWKKTHGKNYQTEVEELGRREVWERNLQLINLHNLEASMDMH
TYDLGMNHMGDMTQEEIAQSFASLRVPADLKREPSAFVGSSGAPIPDTFDWREKGYVTEVKMQGSCGSCW

AFSAVGALEGQLMKTTGKLIDISSQNLVDCSSKYGNKGCNGGFMSQAFQYVIDNQGIDSDQSYPYKGVQQ
QCSYNPAQRAANCSKYSFLPEGDEGVLKEALATIGPISVAIDATRPLFTFYRSGVYNDPTCTKKINHAVL
AVGYGTLGGQDYWLVKNSWSLSWGDQGYIRMSRNKDNQCGIALYGCYPVM
>CTSS1b XM_014200821.1 XP_014056296.1 ssa05 NC_027304.1:56.872.975-56.879.886
MMLWSLLLAVLCGTAVALFDPMLEQHWQMWKKTHGKNYQTEVEELGRREVWERNLQLISLHNLEASMDMH
TYDLGMNHMGDMTQEEIAQSFASLLVPADLKREPSAFVGSSGAPIPDTFDWREKGYVTGVKMQGSCGSCW
AFSSVGALEGQLMKTTGKLIDLSPQNLVDCSSKYGNKGCHGGFMTKAFQYVIDNQGIASDQSYPYKGVQQ
QCIYNPAQRAANCSRYSFLPEGDEGVLKEALATIGPISVGIDATRPSFAFYRSGVYNDPTCTKKTNHAVL
AVGYGTLGGQDYWLVKNSWGLSWGDQGYIRMSRNKDNQCGIALYGCYPVM
>CTSS2 XM_014126453.1 XP_013981928.1 ssa01 NC_027300.1:113.265.513-113.267.831
MTSLYLAVLVLCMSAVYAAHMFDSQLEGHWHLWKNWHSKNYHEREEGWRRIVWEKNLKKIEMHNLDHSMG
KHSYLLGMNHFGDMV
>CTSKa XM_014162370.1 XP_014017845.1 ssa02 NC_027301.1:23.783.794-23.795.107
MLLCGCVLLLLGSVLAHPLIEMSLDAEWDSWKTTHLREYNGLGEEAIRRTIWEKNMRLIEAHNEEAALGI
HSYELGMNHLGDMTSEEIVAKLTGLQVPMNRDRSNTWIPDNNVVKLPRSIDYRKKGMVTPVKNQLSCGSC
WAFSSAGALEGQLAKTTGKLIDLSPQNLVDCVTENNGCGGGYMTNAFEYVEENGGIDTEEAYPYLGQDEQ
CAYNASGVGAQCRGFKEIPEGDEQALTKAVAKVGPVAVGIDATLSSFQFYQRGVYYDPNCNKDDINHAVL
AVGYGQTAKGEKFWIVKNSWSESWGNQGYIKMARNRGNACGIANLASYPIM
>CTSKb NM_001140399.1 NP_001133871.1 ssa05 NC_027304.1:56.864.904-56.871.943
MLLCGCVLLFLGSVLAHPLNEMSLDAQWDSWKTTHLREYNGLGEEVIRRTIWEKNMRLIEAHNEEAALGI
HSYELGMNHLGDMTSEEIAEKLTGLQVPMNRDRSNTWIPDNNVVKIPRSIDYRKKGMVTPVKNQLSCGSC
WAFSSAGALEGQLAKTTGKLIDLSPQNLVDCVTENNGCGGGYMTNAFEYVEENGGIDTEEAYPYLGQDGQ
CAYNASGMGAQCRGFKEIPEGDEWALTKAVVKVGPVAVGIDATLSTFQFYQRGVYYDPNCNKDDINHAVL
AVGYGQTAKGMKFWIVKNSWSESWGKQGYIMMARNRGNACGIANLASYPIM
