# Supplementary material for: Selective Stimulation of Duplicated Atlantic Salmon MHC Pathway Genes by Interferon-Gamma
Source: Front Immunol. 2020 Oct 6;11:571650. doi: 10.3389/fimmu.2020.571650 (PMC7573153; doi:10.3389/fimmu.2020.571650)
Supplement: Supplementary file 6 [file Data_Sheet_6.pdf]

Supplementary file 6 (SF6). Phylogeny of selected deduced amino acid sequences

Table of Content		Page
SF6.1	JAK1 and JAK2	2
SF6.2	STAT1 and STAT2	3
SF6.3	IRF1 and IRF9	4
SF6.4	MX sequences	5
SF6.5	CC chemokine sequences	6
SF6.6	CXC chemokine sequences	7
SF6.7	IFI44 and IFI44.like sequences	8
SF6.8	B2m sequences	9
SF6.9	Proteasome 20S alpha (PSMA) sequences	10
SF6.10	Proteasome 20S beta (PSMB) sequences	11
SF6.11	Calreticulin (CALR)	12
SF6.12	Cathepsin sequences	13

Phylogenies of deduced amino acid sequences from selected genes. Sequence details can be found in Supplementary file 4. The evolutionary histories were inferred using the Neighbor-Joining method [1]. The optimal trees are shown. The percentage of replicate trees in which the associated taxa clustered together in the bootstrap test (100 replicates) are shown next to the branches [2]. The trees are drawn to scale, with branch lengths in the same units as those of the evolutionary distances used to infer the phylogenetic tree. The evolutionary distances were computed using the Poisson correction method [3] and are in the units of the number of amino acid substitutions per site. All ambiguous positions were removed for each sequence pair. Evolutionary analyses were conducted in MEGA7 [4]. Deduced Atlantic salmon amino acid sequences are shown in SF5 and amino acid sequence identity is shown in SF7 while other sequence references are included in parenthesis behind gene name.

1. Saitou N. and Nei M. (1987). The neighbor-joining method: A new method for reconstructing phylogenetic trees. Molecular Biology and Evolution 4:406-425.
2. Felsenstein J. (1985). Confidence limits on phylogenies: An approach using the bootstrap. Evolution 39:783-791.
3. Zuckerkandl E. and Pauling L. (1965). Evolutionary divergence and convergence in proteins. Edited in Evolving Genes and Proteins by V. Bryson and H.J. Vogel, pp. 97-166. Academic Press, New York.
4. Kumar S., Stecher G., and Tamura K. (2016). MEGA7: Molecular Evolutionary Genetics Analysis version 7.0 for bigger datasets.Molecular Biology and Evolution 33:1870-1874.

SF6.1 JAK1 and JAK2 phylogeny

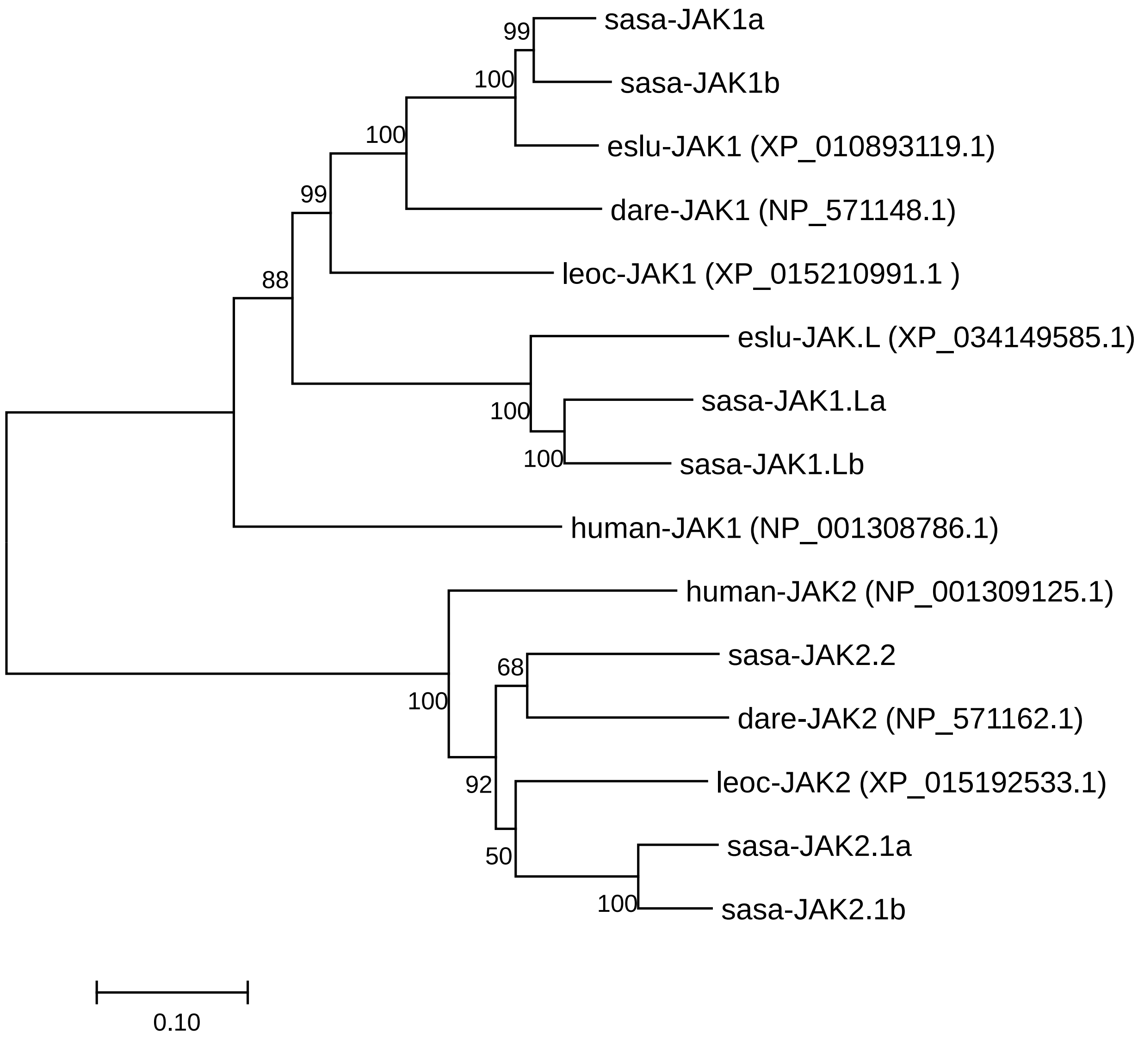

SF6.1. Evolutionary relationships of selected JAK1 and JAK2 sequences
The optimal tree with the sum of branch length = 2.52749142 is shown. The analysis involved 15 amino acid sequences. There were a total of 1269 positions in the final dataset. Sasa is *Salmo salar,* eslu is *Esox Lucius,* dare is *Danio rerio,*Leoc is *Lepisosteus oculatus*.

SF6.2 STAT and STAT2 phylogeny

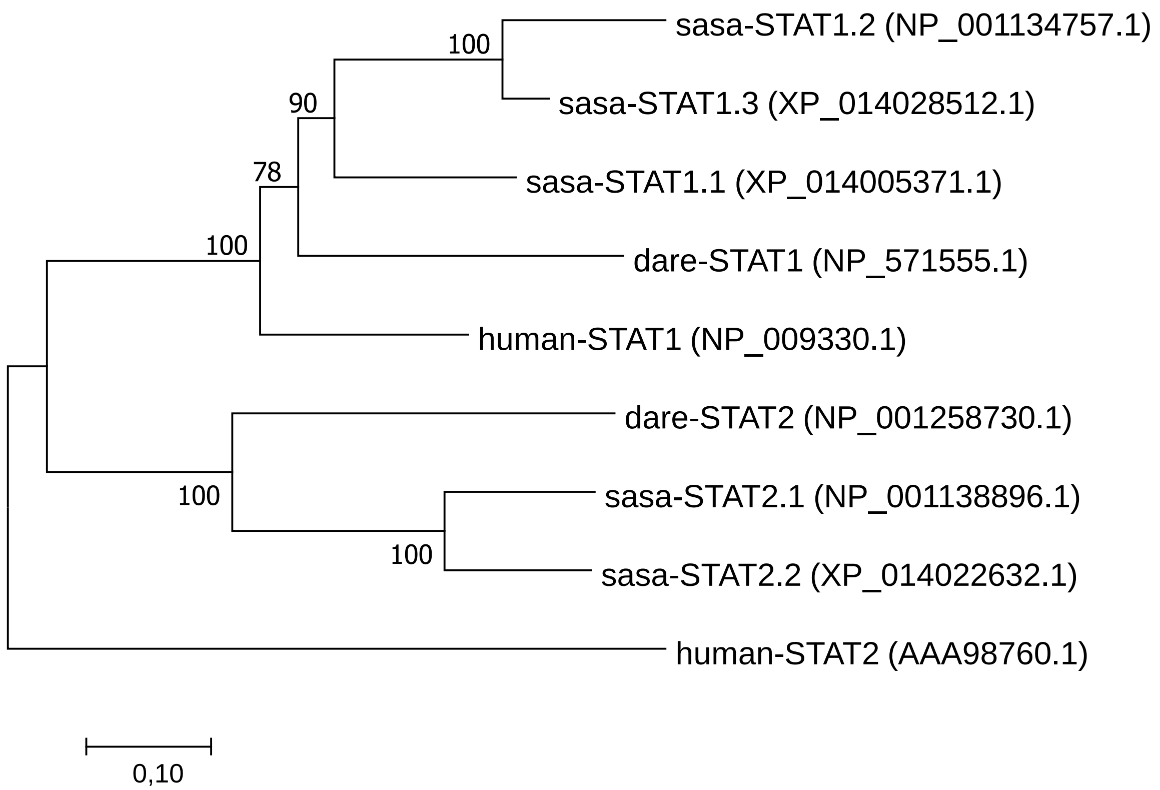

SF6.2. Evolutionary relationships of selected STAT1 and STAT2 sequences
The optimal tree with the sum of branch length = 2,52521329 is shown. The analysis involved 9 amino acid sequences. There were a total of 888 positions in the final dataset. Sasa is *Salmo salar*.

SF6.3 IRF1 and IRF9 phylogeny

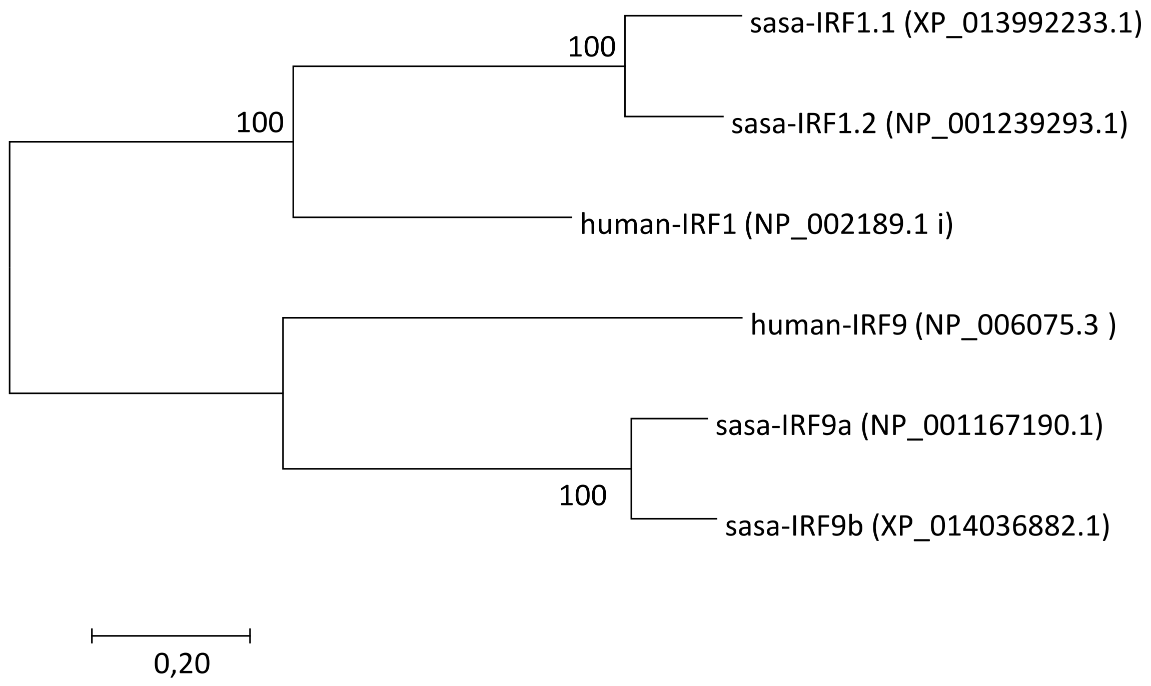

SF6.3 Evolutionary relationships of selected IRF1 and IRF9 sequences
The optimal tree with the sum of branch length = 2,97910757 is shown. The analysis involved 6 amino acid sequences. There were a total of 440 positions in the final dataset. Sasa is *Salmo salar*.

SF6.4 MX phylogeny

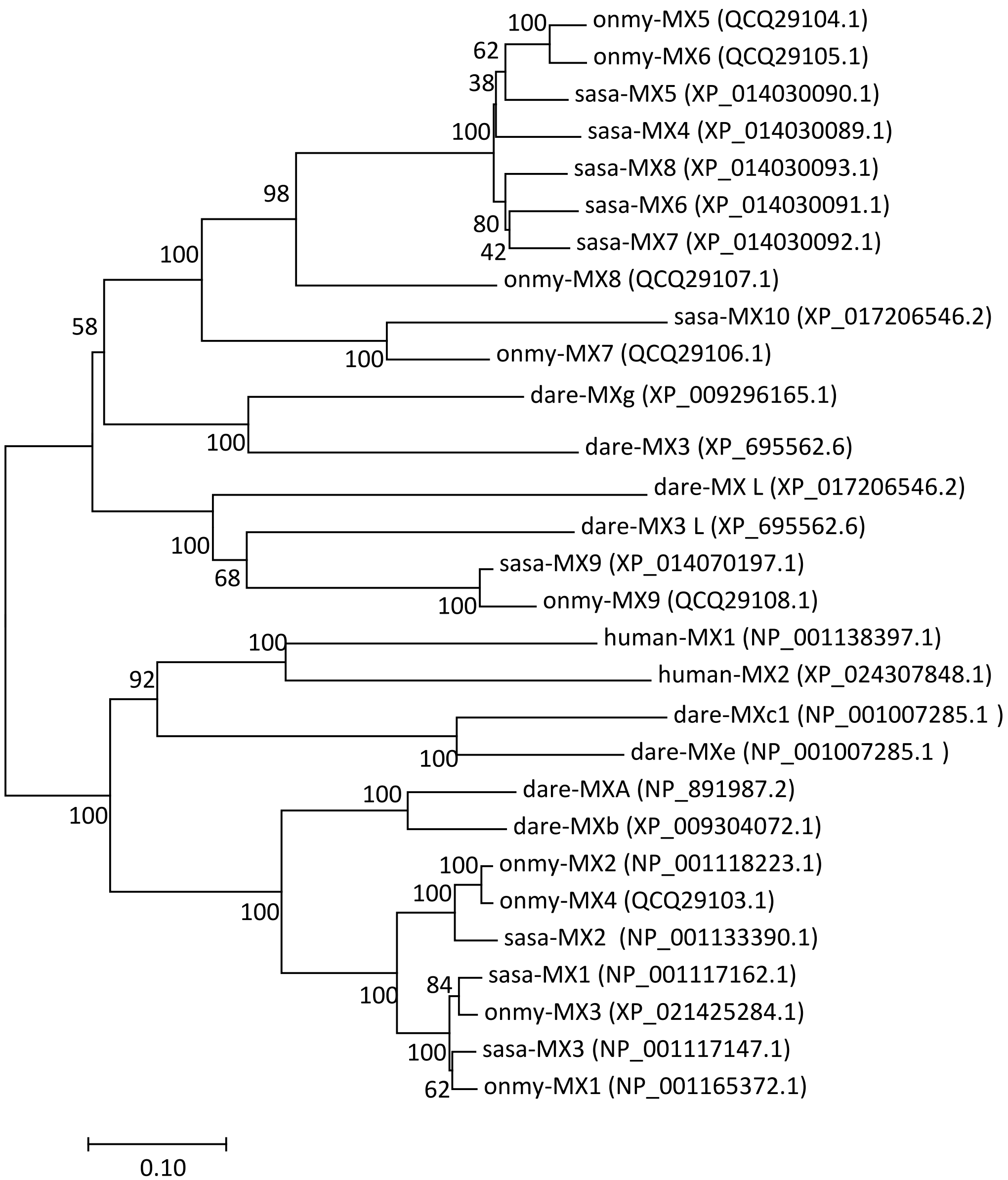

SF6.4. Evolutionary relationships of selected MX sequences
The optimal tree with the sum of branch length = 4,53302212 is shown. The analysis involved 29 amino acid sequences. There were a total of 749 positions in the final dataset. Atlantic salmon MX sequences are from main text reference Robertsen et al., 2019 while rainbow trout and zebrafish MX sequences are from main text reference Wang et al.2019. Sasa is *Salmo salar,* dare is *Danio Rerio,* onmy is *Oncorhynchus mykiss*.

SF6.5 CC chemokine phylogeny

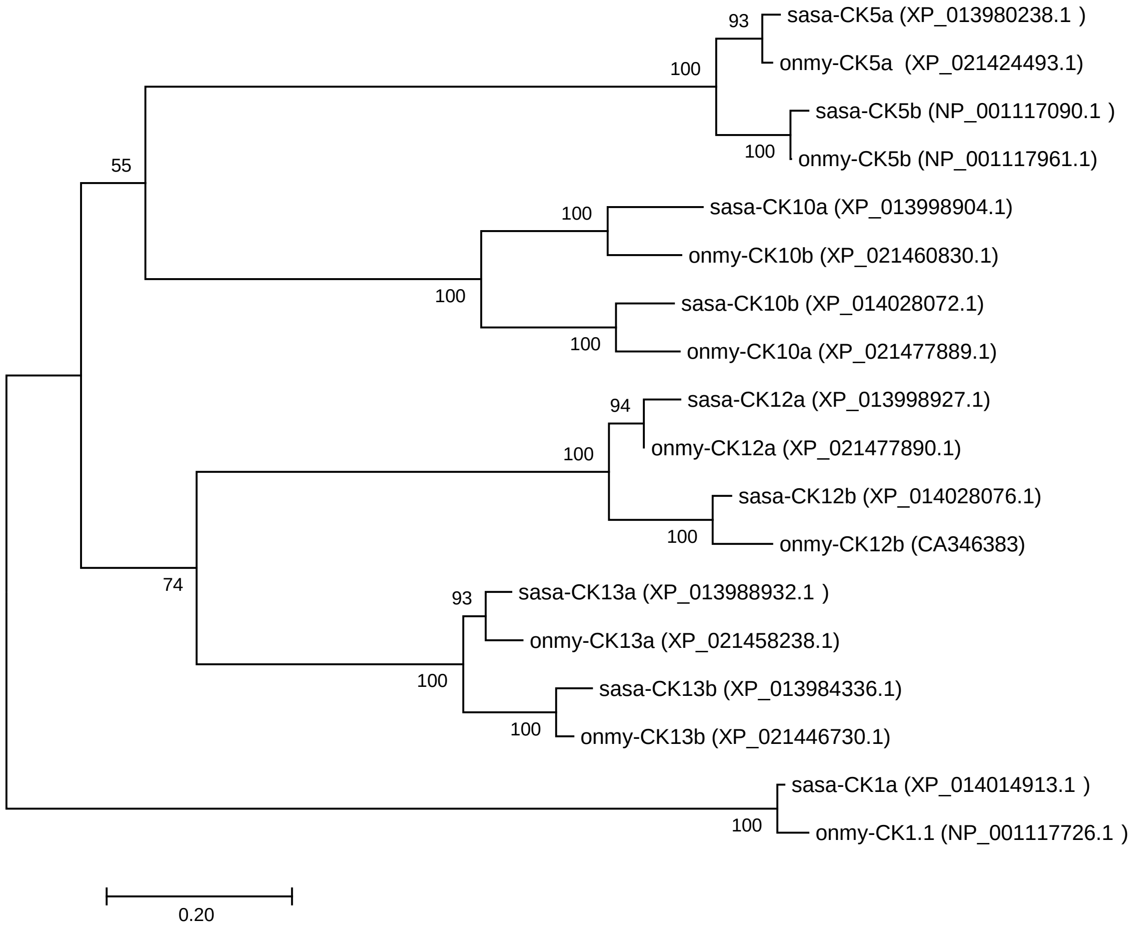

SF6.5. Evolutionary relationships of selected CC chemokine sequences
The optimal tree with the sum of branch length = 4.15661126 is shown. The analysis involved 18 amino acid sequences. There were a total of 254 positions in the final dataset. Rainbow trout chemokine sequences are from main text reference Laing and Secombes 2004. Sasa is *Salmo salar,* onmy is *Oncorhynchus mykiss*.

SF6.6 CXC chemokine phylogeny

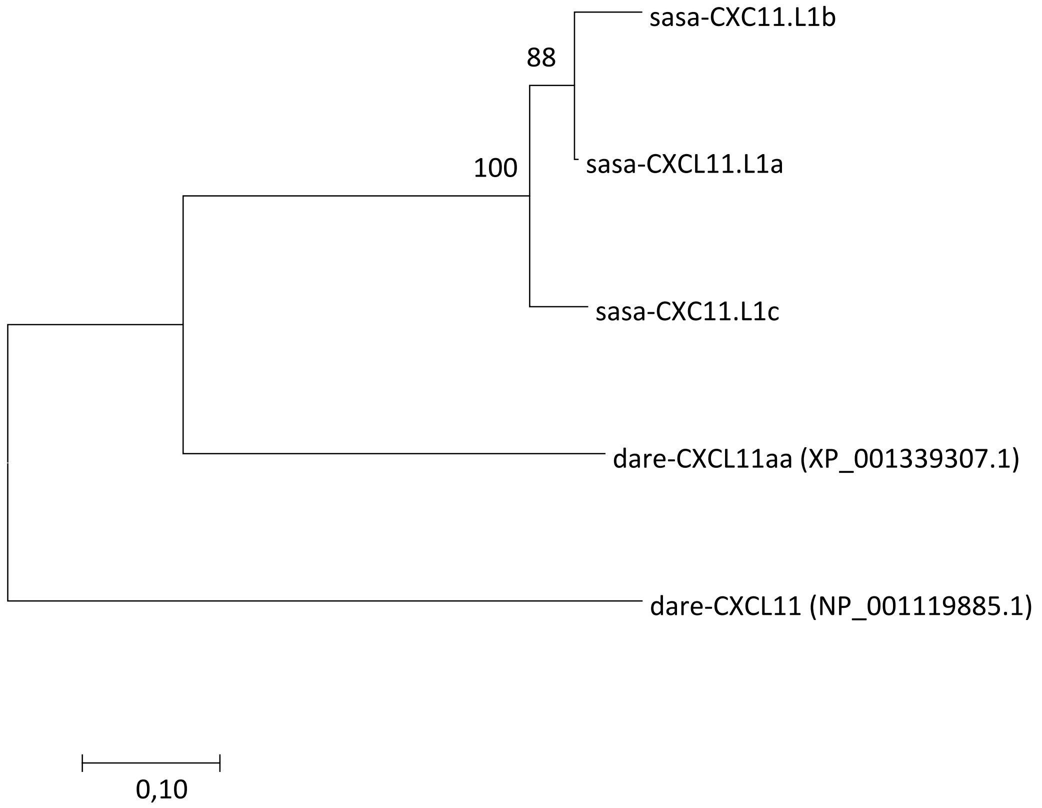

SF6.6. Evolutionary relationships of selected CXC chemokine sequences
The optimal tree with the sum of branch length = 1,27275822 is shown. The analysis involved 5 amino acid sequences. All ambiguous positions were removed for each sequence pair. There were a total of 162 positions in the final dataset. Zebrafish sequences are from main text reference Chen et al.2013. Sasa is *Salmo salar,* dare is *Danio rerio*.

SF6.7 IFI44 and IFI44.L phylogeny

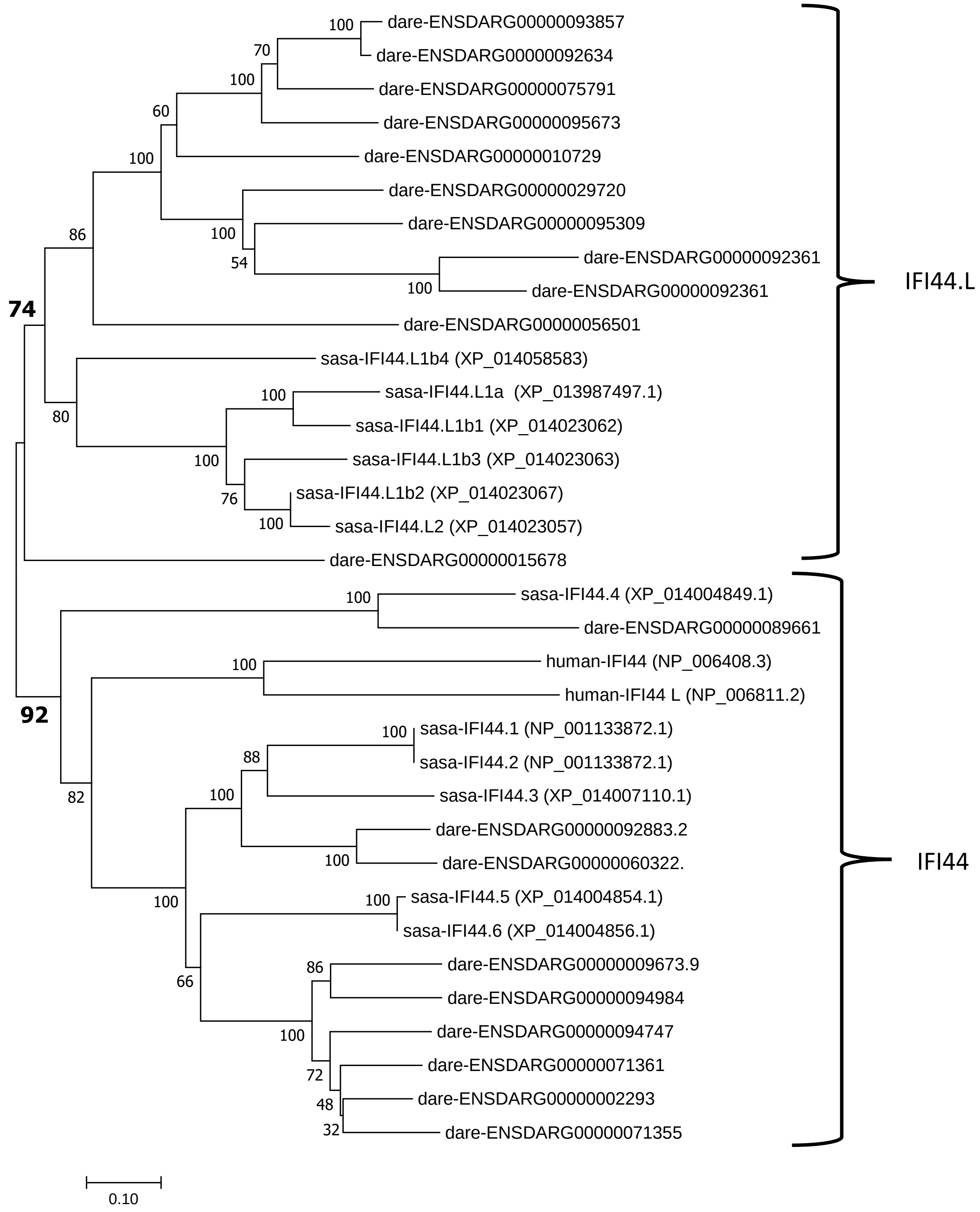

SF6.7. Evolutionary relationships of IFI44 and IFI44.L sequences
The optimal tree with the sum of branch length = 8,31579298 is shown. The analysis involved 34 amino acid sequences. There were a total of 533 positions in the final dataset. Zebrafish sequences are from main text reference Briolat et al.(2014).
Sasa is *Salmo salar,* dare is *Danio rerio*.

SF6.8. b2m phylogeny

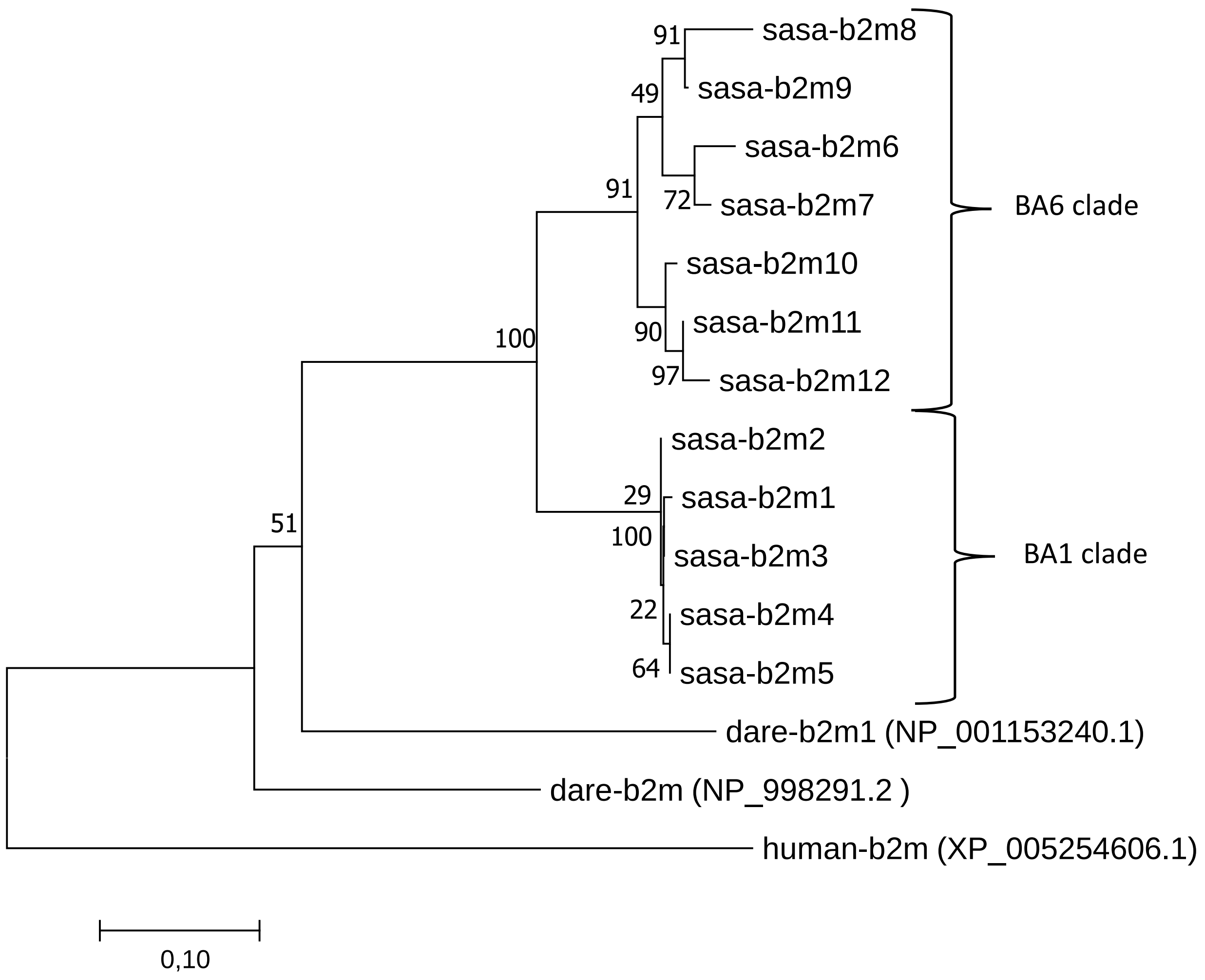

SF6.8. Evolutionary relationships of selected b2m sequences
The optimal tree with the sum of branch length = 1,57194525 is shown. The analysis involved 15 amino acid sequences. There were a total of 153 positions in the final dataset. Sasa is *Salmo salar,* dare is *Danio rerio*.

SF6.9. Atlantic salmon PSMA phylogeny

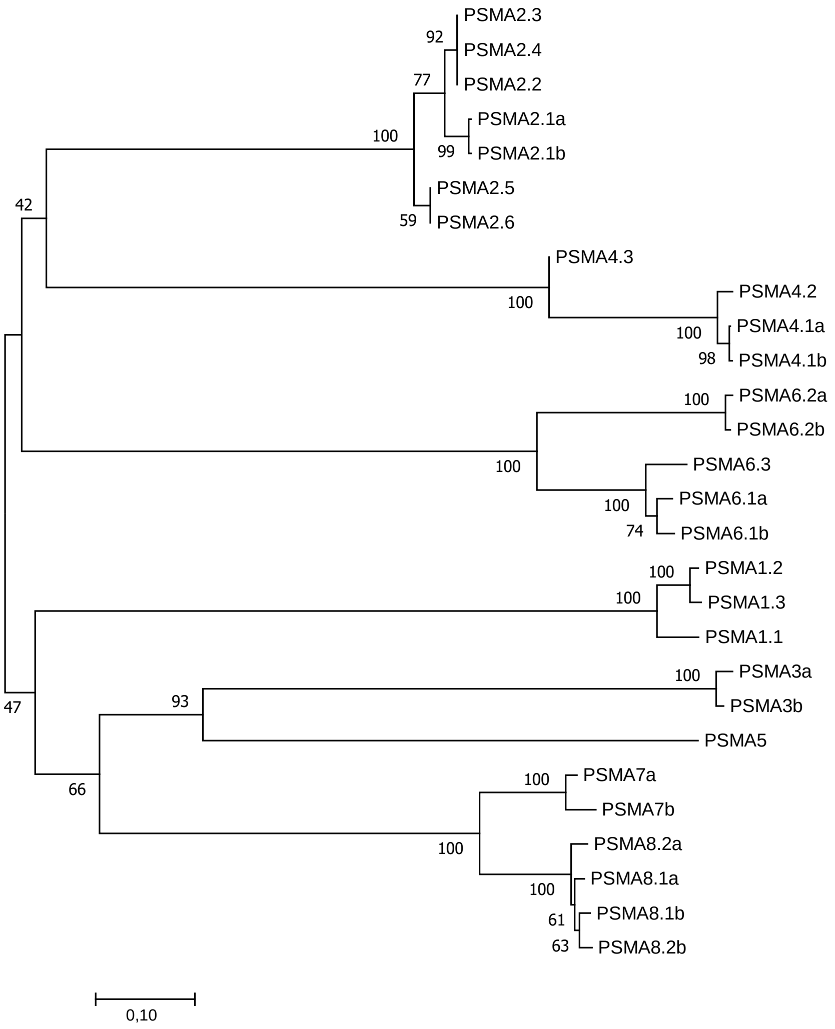

SF6.9. Evolutionary relationships of Atlantic salmon PSMA sequences
The optimal tree with the sum of branch length = 4,77123855 is shown. The analysis involved 28 amino acid sequences. There were a total of 274 positions in the final dataset.

SF6.10. Atlantic salmon PSMB phylogeny

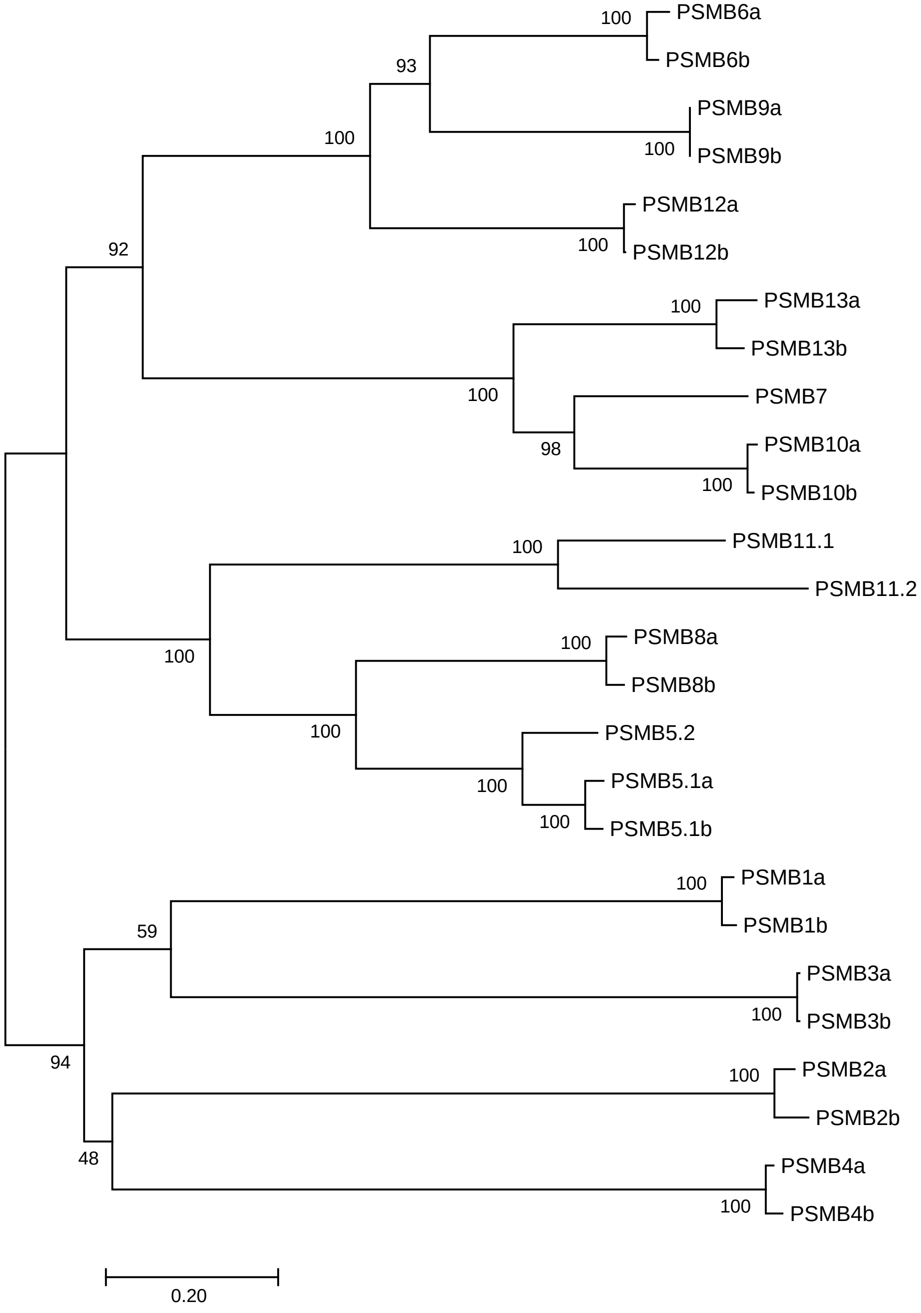

SF6.10. Evolutionary relationships of Atlantic PSMB amino acid sequences
The optimal tree with the sum of branch length = 7.81136632 is shown. The analysis involved 26 amino acid sequences. There were a total of 464 positions in the final dataset.

SF6.11. CaLreticulin phylogeny

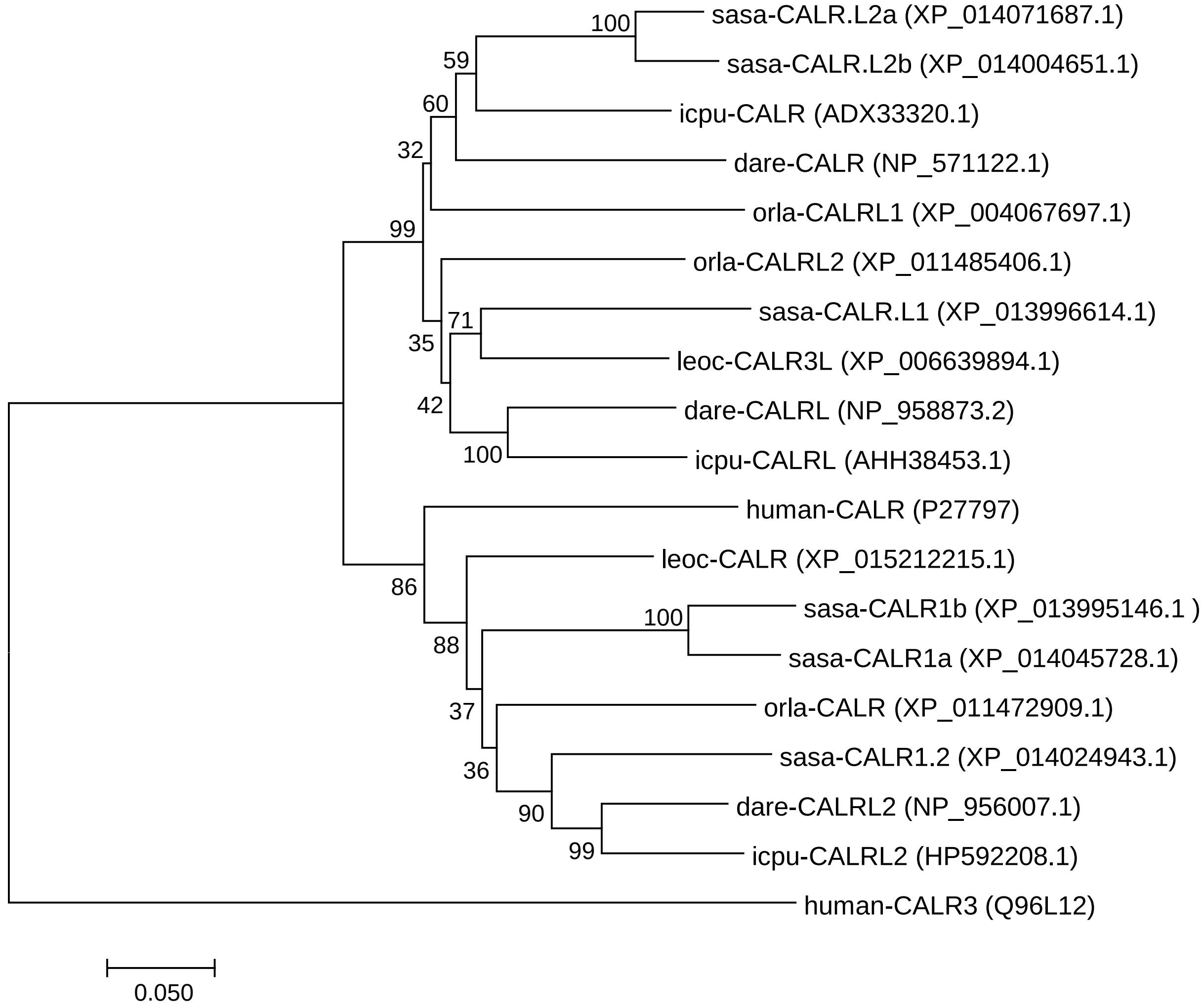

SF6.11. Evolutionary relationships of calreticulin sequences
The optimal tree with the sum of branch length = 2.51431860 is shown. The analysis involved 19 amino acid sequences. There were a total of 706 positions in the final dataset. Data originate from main text reference Grimholt et al., 2018.

SF6.12. Cathepsin phylogeny

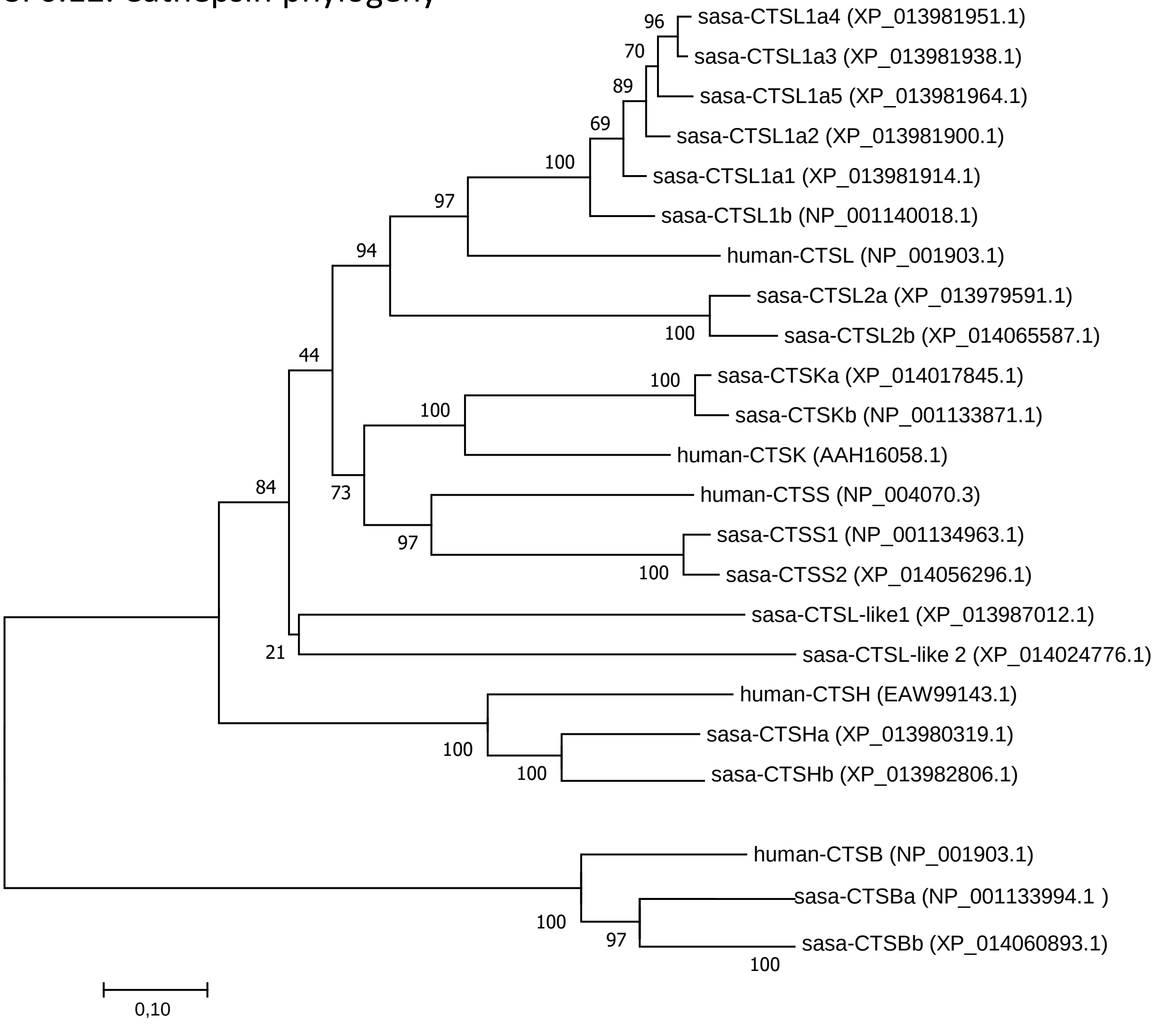

SF6.12. Evolutionary relationships of selected cathepsin amino acid sequences
The optimal tree with the sum of branch length = 5,56953869 is shown. The analysis involved 23 amino acid sequences. There were a total of 581 positions in the final dataset. sasa is *Salmo salar*.
