# Supplementary material for: Selective Stimulation of Duplicated Atlantic Salmon MHC Pathway Genes by Interferon-Gamma
Source: Front Immunol. 2020 Oct 6;11:571650. doi: 10.3389/fimmu.2020.571650 (PMC7573153; doi:10.3389/fimmu.2020.571650)
Supplement: Supplementary file 8 [file Data_Sheet_8.pdf]

Supplementary File 8 (SF8). Duplicate Atlantic salmon MHCI regions

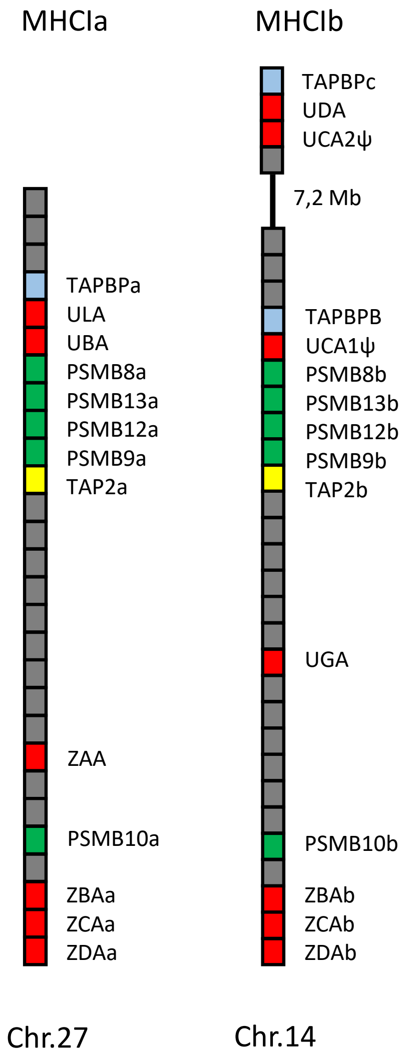

Legend to SF8.
Data are adapted from main text reference Grimholt et al.(2018).
Relevant genes residing in the duplicated MHCIa and MHCIb regions on chromosome 27 and 14 respectively are shown. Genes are shown as color coded boxes where light blue are TAPBP genes, red are MHCI genes, green are PSMB genes and yellow are TAP genes.
